# Structure, expression profile and phylogenetic inference of chalcone isomerase-like genes from the narrow-leafed lupin (*Lupinus angustifolius* L.) genome

**DOI:** 10.3389/fpls.2015.00268

**Published:** 2015-04-21

**Authors:** Łucja Przysiecka, Michał Książkiewicz, Bogdan Wolko, Barbara Naganowska

**Affiliations:** ^1^Department of Genomics, Institute of Plant Genetics of the Polish Academy of SciencesPoznań, Poland; ^2^NanoBioMedical Centre, Adam Mickiewicz UniversityPoznań, Poland

**Keywords:** chalcone isomerase, cytogenetics, duplication, evolution, gene expression, genetic mapping, *Lupinus angustifolius*, synteny

## Abstract

Lupins, like other legumes, have a unique biosynthesis scheme of 5-deoxy-type flavonoids and isoflavonoids. A key enzyme in this pathway is chalcone isomerase (CHI), a member of CHI-fold protein family, encompassing subfamilies of CHI1, CHI2, CHI-like (CHIL), and fatty acid-binding (FAP) proteins. Here, two *Lupinus angustifolius* (narrow-leafed lupin) *CHILs*, *LangCHIL*1 and *LangCHIL*2, were identified and characterized using DNA fingerprinting, cytogenetic and linkage mapping, sequencing and expression profiling. Clones carrying *CHIL* sequences were assembled into two contigs. Full gene sequences were obtained from these contigs, and mapped in two *L. angustifolius* linkage groups by gene-specific markers. Bacterial artificial chromosome fluorescence *in situ* hybridization approach confirmed the localization of two *LangCHIL* genes in distinct chromosomes. The expression profiles of both *LangCHIL* isoforms were very similar. The highest level of transcription was in the roots of the third week of plant growth; thereafter, expression declined. The expression of both *LangCHIL* genes in leaves and stems was similar and low. Comparative mapping to reference legume genome sequences revealed strong syntenic links; however, *LangCHIL*2 contig had a much more conserved structure than *LangCHIL*1. *LangCHIL*2 is assumed to be an ancestor gene, whereas *LangCHIL*1 probably appeared as a result of duplication. As both copies are transcriptionally active, questions arise concerning their hypothetical functional divergence. Screening of the narrow-leafed lupin genome and transcriptome with CHI-fold protein sequences, followed by Bayesian inference of phylogeny and cross-genera synteny survey, identified representatives of all but one (*CHI1*) main subfamilies. They are as follows: two copies of *CHI2*, *FAPa2* and *CHIL*, and single copies of *FAPb* and *FAPa1*. Duplicated genes are remnants of whole genome duplication which is assumed to have occurred after the divergence of *Lupinus*, *Arachis*, and *Glycine*.

## Introduction

Flavonoids are plant secondary metabolites that have received increased interest in recent years. They are involved in various biological processes, such as flower pigmentation, protection against ultraviolet irradiation, male fertility, cell-cycle regulation, auxin transport, protection against osmotic and thermal stresses, and pathogen attack (Woo et al., 2005; Peer and Murphy, 2007). Flavonoids also play a key role in symbiotic interactions between plants and microbes, e.g., in mycorrhiza (Harrison and Dixon, 1993), actinorhiza (Laplaze et al., 2000), and diazotrophy (Subramanian et al., 2007). The antioxidant activity of flavonoids toward free radicals and reactive oxygen species, and their potential estrogenic and anticancer activity, such as antiproliferation, promotion of differentiation, and apoptosis, highlight their health-protecting role in the human diet and animal feed. Flavonoids are used in the pharmaceutical and cosmetic industry, as well as in plant protection chemicals (Havsteen, 2002; Lin and Weng, 2006; Schmidt et al., 2006).

The biosynthetic pathway of flavonoids involves several key enzymes (Weisshaar and Jenkins, 1998). One such is chalcone isomerase (CHI, EC 5.5.1.6), which is involved in the very early phase of the flavonoid biosynthesis pathway. CHI follows the action of chalcone synthase (CHS) and catalyzes the stereo-specific isomerization of chalcones into their (2S)-flavanones, e.g., naringenin and liquiritigenin. CHIs are classified into two types with highly family-specific distributions. CHIs belong to the large CHI-fold protein family, which can be subdivided into mentioned CHIs (encompassing type I and type II CHI), CHI-like (CHILs), and fatty acid-binding proteins (FAPs) (Ngaki et al., 2012). CHI type I is typical of higher plants, but CHI type II is specific to leguminous plants and is responsible for synthesizing flavonoids interacting with rhizobia during diazotrophic symbiosis (Shimada et al., 2003; Subramanian et al., 2007). The protein structure of CHI was elucidated (Jez et al., 2000). In most plant species, a few genes encode CHI, while multiple genes encode CHS (Holton and Cornish, 1995; Chu et al., 2014). In general, legumes have more *CHI* genes than non-legume plants (Shimada et al., 2003; Ralston et al., 2005; Chu et al., 2014).

The CHI-like isoform (CHI type IV, CHI4), comparing to CHIs type I (CHI1) and II (CHI2), has substitutions of several catalytic residues. On the other hand, it has more sterically restricted pockets surrounding the ligand-binding clefts than FAP (type III CHI), and has several polar residues that in conjunction interact with the substrates through hydrogen bonds (Ngaki et al., 2012). CHILs are found only in land plants and their function is still not clear. However, their role as enhancer of flavonoid production as well as flower pigmentation is indicated (Morita et al., 2014). It is noteworthy, that CHIs of the same type from different species show 70% similarity, while the sequence of various CHI types are only about 50% identical (Shimada et al., 2003).

The legume *Lupinus angustifolius* L. (narrow-leafed lupin) belongs to the genus *Lupinus* (tribe of Genisteae, family Fabaceae, subfamily Faboideae). It is believed that lupins are paleopolyploids (Atkins et al., 1998; Gladstones, 1998), created by allo- or autopolyploidization of ancestral genomes, followed by the process of differentiation and diploidization (Wendel, 2000). Contemporary species are mostly functional diploids, but their ploidy level has not been fully determined (Wolko et al., 2011). The narrow-leafed lupin, representing cool season legume species, was chosen for cytological and molecular studies because of its relatively low chromosome number (2*n* = 40) and small genome size (2*C* = 1.89 pg), compared with other lupins (Naganowska et al., 2003). Linkage maps with microsatellite-anchored fragment length polymorphisms (Boersma et al., 2005) and gene-based sequence tagged site (STS) markers (Nelson et al., 2006) have been constructed, which were further supplemented with other STS markers and merged to form reference genetic maps of the narrow-leafed lupin genome (Nelson et al., 2010; Kroc et al., 2014). Bacterial artificial chromosome (BAC) libraries of the nuclear genomes for two *L. angustifolius* cultivars: Polish cv. Sonet (Kasprzak et al., 2006) and Australian cv. Tanjil (Gao et al., 2011) were developed. BAC analysis and cytogenetic experiments resulted in the integration of 12 linkage groups with the corresponding chromosomes, as well as the identification of several gene-rich regions (Kaczmarek et al., 2009; Lesniewska et al., 2011; Książkiewicz et al., 2013, 2015). A specific bioinformatic pipeline has been developed to aid the analysis and annotation of lupin sequence data (Zielezinski et al., 2012). A draft assembly covering approximately 50% of the lupin genome was released (Yang et al., 2013). The development of reference transcriptome data for two closely related lupin species: *L. albus* (O'Rourke et al., 2013) and *L. luteus* (Parra-González et al., 2012) enhanced the possibility of targeting a specific gene in the narrow-leafed lupin genome. Lupin genes can be identified directly using sequence information from model plant species; screening of the narrow-leafed lupin cDNA library with *Glycine max* and *Arabidopsis thaliana* gene-derived probes indicated highly conserved gene structures among these species (Francki and Mullan, 2004). Comparative genomic studies between *L. angustifolius* and *G. max* identified a high level of microsynteny in the gene-rich regions. Not only is the gene nucleotide sequence conserved, but also the order and orientation of particular genes in syntenic blocks (Książkiewicz et al., 2013).

Herein, *Lupinus* genus genomic resources were used to identify chalcone isomerase-like genes (*LangCHILs*), analyze the structures of their genome regions, and survey their expression profiles. New gene-specific markers were designed and multipoint linkage analysis localized genes in linkage groups of the genetic map. The relative positions of genes in lupin chromosomes were resolved by fluorescence *in situ* hybridization (FISH). Synteny analysis of regions carrying *LangCHILs* and other CHI-fold protein genes using model and reference legume species was conducted. Phylogenetic insight into the whole CHI-fold protein family was performed. Finally, *LangCHIL* gene expression patterns in different plant organs and expression changes during plant growth were determined.

## Materials and methods

### Germplasm resources

Seeds of *L. angustifolius* cv. Sonet were obtained from the Polish Lupin Gene Bank at the Breeding Station Wiatrowo (Poznan Plant Breeders Ltd, Poland, Tulce). Seeds of the mapping population comprised 89 F_8_ recombinant inbred lines (RILs) developed from the cross combination 83A:476 (domestic) × P27255 (wild-type) *L. angustifolius* (Boersma et al., 2005) were kindly provided by Dr. Hua'an Yang, Department of Agriculture and Food, Western Australia.

### Plant nucleic acid isolation

Isolation of total RNA was performed using a robotic workstation QIAcube (Qiagen, Hilden, Germany) and the RNeasy Plant Mini Kit (Qiagen). The DNeasy Plant Mini Kit (Qiagen) was used to isolate genomic DNA from *L. angustifolius* leaves. Agarose gel electrophoresis followed by ethidium bromide staining and spectrophotometer (NanoDrop 2000; ThermoScientific, Waltham, MA, USA) analysis were used to measure the quality and concentration of the RNA and DNA.

### Hybridization probe and BAC library screening

EST sequence from *Lupinus albus* (Acc. No. CA410672) (Uhde-Stone et al., 2003) was aligned to NCBI reference RNA sequence collection using BLAST. Based on the information from this annotation, specific primers generating product for *L. angustifolius* were designed. The primer sequences were as follows: CHIL_F: 5′-TGATCAAAGAGATCAAGGGTGCTC-3′, CHIL_R: 5′-TACCTCTGCAGTTTGAGAATCAGC-3′.

PCR with an annealing temperature of 56°C was used to label probes with P^32^ dATP (MP Biomedicals). The probe was purified using a QIAquick PCR Purification Kit (Qiagen), denaturated at 94°C for 5 min and incubated on ice.

Hybridization of the labeled probe with three macroarrays containing the whole nuclear genome BAC library of *L. angustifolius* cv. Sonet (Kasprzak et al., 2006) was conducted in 5 × SSPE buffer with 0.5% SDS at 50°C overnight. Filters were washed for 15 min at 50°C in solutions of increasing stringency (5 × SSC + 0.5% SDS; 2.5 × SSC + 0.25% SDS; 1 × SSC + 0.1% SDS; 0.5 × SSC + 0.05% SDS). Blots were exposed on BAS-MS 2340 imaging plates (Fujifilm, Tokyo, Japan) for 48 h and analyzed using the FLA-5100 phosphorimager (Fujifilm).

### Isolation of DNA from BAC clones

The DNA from BAC clones showing positive hybridization signals was isolated using the QIAprep Spin Miniprep Kit (Qiagen) and verified by PCR using CHIL_F and CHIL_R primers and insert DNA template. *Not*I was used to cut inserts from clones. Digestion was conducted for 2.5 h at 37°C and stopped by incubation at 80°C for 20 min. Pulsed field gel electrophoresis, which was performed for 16 h at 5.2 V/cm with 0.5–40 s switch time, determined the size and quality of DNA inserts. Size markers Lambda Ladder PFG Marker (New England BioLabs, Ipswich, MA) and GeneRuler™ 1 kb Plus (Fermentas Waltham, MA, USA) were used.

### BAC-end sequencing and functional annotation

BAC-ends were sequenced on an ABI PRISM 3130 XL Genetic Analyzer (Applied Biosystems, Foster City, CA, USA) using pIndigoBAC5 sequencing primers: 5′ end (BAC5) CTCGTATGTTGTGTGGAATTGTGAGC and 3′ end (BAC3) GGATGTGCTGCAAGGCGATTAAGTTGG. The BAC-end sequences (BESs) obtained using the BAC3 and BAC5 primers were given the “_3” and “_5” suffixes, respectively.

The bioinformatic analysis of BESs included a nucleotide similarity search of homologous sequences. RepeatMasker (http://www.repeatmasker.org) was first used to annotate and mask repeat sequences. To identify homologous coding sequences, BESs were compared to sequences deposited in NCBI gene databases using blast2go (Conesa et al., 2005) and BLASTN 2.2.26 +/BLASTX 2.2.26 + algorithms.

### BAC clone fingerprinting and contig assembly

Each BAC was digested by two different restriction enzymes, *Hind*III and *Eco*130I, using 2 units of enzyme for 1 μg of clone DNA. The reaction was performed at 37°C for 16 h. Electrophoresis of digested products was performed in 1% agarose gels (Sigma, USA), at 0.09 kV for 20 h. Image 3.10b (Sulston et al., 1989) and FPC V8.5.3 (Soderlund et al., 1997) software were used to construct the contigs, with a tolerance value of 3 and a cut-off of 1e-10.

### *LangCHIL* gene sequencing

Products amplified using *LangCHIL* primers from BAC insert DNA were sequenced, and obtained sequences were compared with the probe sequence. Sequencing of two selected BAC clones was performed using chromosome walking, with new primers anchored in the gene sequences, designed for both ends. Subsequent new primer design and clone sequencing were repeated to recover the entire gene sequences. Designed primers used for gene sequencing are listed in Supplementary Table 1. Fgenesh (Salamov and Solovyev, 2000) was used to determine the structure of *LangCHILs*, with a reference to the available NCBI EST and transcriptome sequence data of lupin and other legumes. FancyGene software (Rambaldi and Ciccarelli, 2009) was used to illustrate the *LangCHIL* genes structure.

### Chromosomal localization of genes

Root tip meristems were used to observe mitotic metaphase chromosomes. Chromosome squashes and the FISH procedure were performed according to the protocol (Lesniewska et al., 2011), with minor modifications. These included: probe labeling with digoxygenin-11-dUTP and tetramethyl-rhodamine-5-dUTP by incubation at 15°C for 110 min followed by inactivation at 65°C for 15 min using Sensoquest Labcycler (Göttingen, Germany) and hybridization at 37°C for 22 h.

### Developing of gene-based PCR markers

PCR primers anchored in gene sequences were designed: CHIL_1_28O01_F: 5′-GGCTGATGAAGTGGTATTGGTT-3′, CHIL_1_28O01_R: 5′-AGCCAAAGAAGCAATGGTTG-3′, CHIL_2_5L11_F: 5′-ACCAAGCCCCTATCTCTGCT-3′, CHIL_2_5L11_R: 5′-GCTCTGGAACCACCAAGGTA-3′.

PCR was performed using DNA isolated from parental lines of the *L. angustifolius* mapping population. The PCR annealing temperature was 58°C for the CHIL_1 primers and 54°C for the CHIL_2. The PCR products were purified and sequenced. Based on the PCR product sequence alignments, new primers were designed to target the DNA polymorphism between parental lines: CHIL_1_28O01_F: 5′-TCAAGGGTGCTCAGTATGGG-3′ and CHIL_1_28O01_R: 5′-TCATCCTTTCCCTCCAAAGA-3′ for CHIL1 marker, and CHIL_2_5L11_F: 5′-TTTTCATCTTTAATCAGGGATTAT-3′, CHIL_2_5L11_R: 5′-GTGGCCAATGCAGTTGCAAA-3′ for CHIL2. The polymorphic CHIL1 product was amplified at an annealing temperature of 56°C. The CHIL2 marker containing a single nucleotide polymorphism (SNP) was scored by a derived Cleaved Amplified Polymorphic Sequence (dCAPS) approach (Neff et al., 1998). Restriction sites were identified in dCAPS Finder 2.0 software (Neff et al., 2002). PCR was conducted at an annealing temperature of 52°C. PCR products were digested with *Taq*I (Fermentas) at 65°C for 3 h. The polymorphism was visualized by electrophoresis (1.5% agarose gel stained with ethidium bromide).

### Linkage mapping of *LangCHIL* genes

The segregation of markers, tested among all RILs of the mapping population, was analyzed using MapManager v. QTXb20 (Manly et al., 2001). New markers were distributed in existing linkage groups (map function Kosambi, linkage criterion 1e-4) of the reference genetic map of *L. angustifolius* (Kroc et al., 2014). MapChart software (Voorrips, 2002) was used to draw the *Lupinus* linkage groups containing new *LangCHIL* markers.

### Organ-specific expression survey of *LangCHIL* genes

Lupin seeds were sown in pots with soil in a growth chamber under controlled conditions (temperature 18°C, photoperiod 12/12 h). After 21 days of growth, plant samples were harvested in triplicate, and the roots, stems, and leaves were separated, every week for 4 weeks. The collected material was frozen in liquid nitrogen and stored at −80°C until use. RT-PCR was performed using *LangCHIL*1 and *LangCHIL*2 gene-specific primers or *18S rRNA- and* β*-TUB-*specific primers. 18S rRNA and β-tubulin were selected as reference genes to normalize *LangCHIL* gene expression values. Genomic DNA was used as a control template to rule out its possible contamination in each cDNA sample. The cDNA samples for real-time RT-PCR experiments were synthesized from 1 μ g of total RNA and anchored-oligo(dT)_18_ primers, using the Transcriptor First Strand cDNA Synthesis Kit (Roche Diagnostics, Indianapolis, IN, USA).

The real-time amplification reactions with the SYBR Green detection chemistry were run in triplicate using 96-wells plates and an iCycler CFX98 thermocycler (Bio-Rad, Hercules, CA, USA). Reactions (10 μ l) comprised 1 μ l of template, 1.5 μ l of forward primer, and 0.5 μ l of reverse amplification primer (3:1 proportion), and 5 μ l of Real Time 2 × PCR Mix SYBR B (A&A Biotechnology, Gdynia, Poland). Blank controls were run in triplicate for each master mix. In addition, for each reaction, a calibration curve was determined using each primer pair and selected cDNA as a template, in serial diminishing dilutions (100 ng/μ l, 10 ng/μ l, 1 ng/μ l, 0.1 ng/μ l, 0.01 ng/μ l). Primers used in real-time RT-PCR are shown in Supplementary Table 1.

The conditions were set as follows: initial denaturation step of 95°C for 10 min, followed by 40 cycles of denaturation at 95°C for 45 s, annealing at 60°C for 30 s, and extension at 72°C for 40 s. The amplification process was followed by a melting curve analysis, from 60°C to 95°C, with temperature increasing steps of 0.5°C every 10 s. Baseline and threshold cycles (Ct) were automatically determined using the Bio-Rad iQ Software 3.0. The reaction results were recorded and analyzed using Chromo4™ System software.

### Anchoring BESs to scaffolds of the draft narrow-leafed lupin genome assembly

BESs were used to screen the collection of *L. angustifolius* whole-genome shotgun contigs and scaffolds (Yang et al., 2013), deposited in the NCBI sequence database (Project No. PRJNA179231, assembly version GCA_000338175.1, accessions AOCW01000001–AOCW01191454). A sequence identity cut-off value of 97% was applied. The BLAST algorithm was optimized for highly similar sequences (word size, 28; match/mismatch scores, 1/-2; and gap costs, linear). If two or more BESs were localized to a single scaffold, the appropriate BAC clones were considered to physically overlap.

### Functional annotation of selected scaffolds

Transposable element-related repeats (TEs) were annotated and masked using RepeatMasker Web Server version 4.0.3 with implemented repeat libraries RMLib 20130422 and Dfam 1.2 (A.F.A. Smit, R. Hubley and P. Green, unpublished data, http://www.repeatmasker.org) before gene prediction. The following options were selected: search engine, cross_match; speed/sensitivity, slow; DNA source, *A. thaliana*. DNA sequences were then compared to a database of TE encoded proteins. The functional annotation of the genetic elements encoded in scaffolds included *de novo* detection of specific signals and comparative analyses with known sequences. *In silico* gene prediction was performed using Fgenesh (Salamov and Solovyev, 2000). Basic Local Alignment Search Tool (BLAST, http://blast.ncbi.nlm.nih.gov) was used to examine similarities with integrated, non-redundant, and annotated sequences of genomic DNA, transcripts, and proteins deposited in the RefSeq database (http://www.ncbi.nlm.nih.gov/refseq). Scaffolds were subjected to sequence homology searches against the transcriptome sequences of *L. luteus* young leaves, buds, flowers, and seeds (Parra-González et al., 2012), and *L. albus* roots and leaves (O'Rourke et al., 2013). The following sequence repositories were used: *L. luteus*, http://www.cgna.cl/lupinus (project PRJNA170318, archive SRX159101); *L. albus*, http://comparative-legumes.org (gene index LAGI 1.0). The analysis was performed using the CoGe BLAST algorithm (Lyons et al., 2008) with the following parameters: e-value cut-off, 1e-10; word size, 8; gap existence cost, 5; gap elongation cost, 2; nucleotide match/mismatch scores, 1/-2. For the potential genes, gene prediction models were visualized, manually verified, and refined using DNA Plotter (Carver et al., 2008). To assign gene names, hypothetical coding sequences were extracted from scaffolds and compared with the NCBI RefSeq database. Accessions producing alignments with the lowest *e*-values were selected as references.

### Phylogenetic survey of *L. angustifolius* CHI-fold proteins

Based on the recently published data (Ngaki et al., 2012; Chu et al., 2014; Dastmalchi and Dhaubhadel, 2015; Liu et al., 2015) the reference set of 172 CHI-fold protein sequences has been selected (Supplementary Table 2). These sequences were aligned to the sequence of the narrow-leafed lupin genome (PRJNA179231) and transcriptomes of *L. angustifolius* (PRJNA248164, GBRP00000000.1), *L. albus* (O'Rourke et al., 2013) and *L. luteus* (Parra-González et al., 2012) under 1e-10 *e*-value cutt-of. Identified *L. angustoflius* nucleotide sequences (Supplementary Table 3) were subjected to Fgenesh+ (Salamov and Solovyev, 2000) *in silico* gene prediction with homology link to the CHI-fold protein sequence with the highest alignment *e*-value assigned. The dataset used in subsequent phylogenetic analysis contained 8 *L. angustifolius*, 6 *L. luteus*, 11 *L. albus*, and 48 reference CHI-fold protein sequences. Multiple sequence alignment was done in MAFFT v7.017 (Katoh et al., 2002), whereas re-alignment in MUSCLE (Edgar, 2004). The selection of best-fit models of protein evolution for given alignment was executed in ProtTest (Abascal et al., 2005). Bayesian inference of phylogeny was performed in MrBayes 3.2.2 (Huelsenbeck and Ronquist, 2001). Phylogenetic tree was drawn in Geneious (Kearse et al., 2012). Parameters and settings used in the phylogenetic insight are given in Supplementary Tables 4–6.

### Micro- and macrosynteny analysis

*L. angustifolius* scaffolds carrying *LangCHIL* sequences were masked for repetitive contents and low-complexity regions, and then subjected to comparative mapping to the following genome sequences: *Medicago truncatula* (Young et al., 2011) (strain A17, JCVI v4.0 unmasked, http://www.jcvi.org/medicago/), *Lotus japonicus* (Sato et al., 2008) (v2.5 unmasked, http://www.kazusa.or.jp), *Cicer arietinum* (Varshney et al., 2013) (v1.0 unmasked, http://comparative-legumes.org), *Glycine max* (Schmutz et al., 2010) (JGI v1.1 unmasked, http://www.phytozome.net), *Phaseolus vulgaris* (v0.9, DOE-JGI, and USDA-NIFA, http://www.phytozome.net), and *Cajanus cajan* (Varshney et al., 2012) (project PRJNA72815, v1.0). *L. angustifolius* scaffolds carrying other CHI-fold protein sequences were subjected to the same procedure, however, genome sequences used for comparative mapping included also *Arachis duranensis* (accession V14167) and *Arachis ipaensis* (accession K30076) (Peanut Genome Project). The CoGe BLAST algorithm (Lyons et al., 2008) was used to perform sequence similarity analyses with the following parameters: *e*-value cut-off, 1e-20; word size, 8; gap existence cost, 5; gap elongation cost, 2; nucleotide match/mismatch scores, 1/-2. Microsyntenic blocks were visualized using the Web-based Genome Synteny Viewer (Revanna et al., 2011). The Synmap analysis tool was used (https://genomevolution.org/coge/SynMap.pl) to determine if identified syntenic loci were located in large conserved blocks of *M. truncatula*, *L. japonicus*, *C. arietinum*, *G. max*, *P. vulgaris*, and *C. cajan* genomes. Sequences were compared by the LAST (http://last.cbrc.jp/) algorithm. The DAGChainer algorithm was used to identify syntenic regions between genomes. The search was based on relative gene order, with the maximum distance between two matches set to 20 genes and the minimum number of aligned gene-pairs set to 5. To survey large scale macrosyntenic relationships, the sequences of molecular markers from linkage groups NLL-03 and NLL-15 from the genetic map (Kroc et al., 2014; Książkiewicz et al., 2015) were subjected to comparative mapping using the same legume genome sequences, bioinformatic tools and settings as those applied for scaffold analysis. The macrosyntenic relationships were inferred if at least four homology links from a particular *L. angustifolius* linkage group to a selected legume chromosome were identified.

## Results

### Screening of BAC library to identify clones carrying *CHIL* genes

EST sequence from *L. albus* (CA410672) was annotated as *CHIL*; alignment to *Glycine max* chalcone isomerase 4-like sequence (NM_001249853.1) revealed 86.1% nucleotide identity and 96% coverage (Supplementary Table 7). Screening of the *L. angustifolius* genomic BAC library with a probe based on the *CHIL* gene from white lupin, tagged 10 BAC clones (Supplementary Figure 1). PCR and sequencing of the positive BACs showed that PCR products of clones 5L11 and 134F01 differed from the rest of the analyzed clone products. No PCR amplification product was obtained for clones 12M14 and 41I07. The PFGE analysis determined the BAC insert sizes as 20–250 kb (Supplementary Figure 2), with an average of 88.7 kb.

### Physical mapping of BAC clones

BAC clones that produced similar band patterns when digested with *Eco*130I and *Hind*III were considered to overlap and were grouped into contigs. From the 10 BACs, two contigs were constructed (Supplementary Figure 3). The larger contig (contig 1), comprised six clones (28O01, 88J04, 106M03, 115N04, 115L05, 129C12). Contig 2 comprised two clones (5L11, 134F01). The remaining clones, 12M14 and 41I07, were singletons.

### Functional annotation of BAC-end sequences

BAC-end sequencing generated 16 BAC-end sequences (BESs) from the *CHIL*-containing BACs. BESs were deposited in the DNA Data Bank of Japan (DDBJ; AB749285-AB749300).

Nine BES sequences had similarities to known genetic elements. The 5′ ends of clones 28O01 and 115N04 contained a retrotransposon, whereas the other BESs revealed statistically significant alignments to various genes and proteins of *Lupinus*, *Glycine*, *Phaseolus*, and *Arabidopsis* genera representatives. At the 3′ end of the clone 134F01, the sequence homologous to *G. max CHI* gene was identified. A list of the identified genes and proteins in the BACs is shown in Table 1.

**Table 1:** **BAC-end sequence (BES) annotation**.

**BES name and accession**	**Database (name, accession number)**	**Name of sequence, source organism**	**Length**	**Identity (%)**	***e*-value**
5L11_5 [DDBJ:AB749286]	RepeatMasker	LTR	618 bp	98	0.0
		Ty1/Copia			
28O01_3 [DDBJ:AB749287]	Swissprot, Q9C9Z8.1	V-type proton ATPase subunit E2, *A. thaliana*	34 AA	73	7e-04
	Nr, XM_003533031.1	V-type proton ATPase subunit E-like, *G. max*	103 bp	77	5e-12
28O01_5 [DDBJ:AB749288]	Swissprot, Q9LRT1.1	Probably inactive leucine-rich repeat receptor-like protein kinase, *A. thaliana*	226 AA	69	3e-66
	Nr, XM_003522462.1	Probably inactive leucine-rich repeat receptor-like protein kinase, *G. max*	680 bp	89	0.0
88J04_3 [DDBJ:AB749289]	Nr, AF119410.1	1-aminocyclopropane-1-carboxylate synthase 4 (ACS4) gene, *L. albus*	157 bp	76	8e-22
106M03_5 [DDBJ:AB749292]	Nr, AF002277.1	LlPR10.1A (Ypr10.1a) gene*, L. luteus*	98 bp	76	1e-7
115N04_3 [DDBJ:AB749295]	Swissprot, Q9LRT1.1	Probably inactive leucine-rich repeat receptor-like protein kinase, *A. thaliana*	277 AA	64	1e-72
	Nr, XM_003522462.1	Probably inactive leucine-rich repeat receptor-like protein kinase, *G. max*	828 bp	87	0.0
115N04_5 [DDBJ:AB749296]	RepeatMasker	LTR/Gypsy Gypsy3_MT_pol	680 bp	71	9e-76
129C12_3 [DDBJ:AB749297]	Nr, HQ632856.1	Rpp4 Asian soybean rust resistance gene locus, *P. vulgaris*	257 bp	77	2e-46
134F04_3 [DDBJ:AB749299]	Nr, NM_001249853	Chalcone isomerase 4-like, *G. max*	333 bp	77	3e-64

### The sequence and structure of genes encoding *CHIL*s

**Figure 1:**
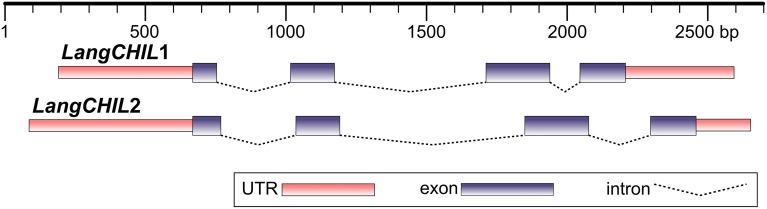
**Structure of two *L. angustifolius* chalcone isomerase-like genes**. Distances are shown in base pairs.

Two BAC clones from different contigs, 28O01 from contig 1 and 5L11 from contig 2, were partially sequenced using chromosome walking method. This established the whole sequence of the *LangCHIL* genes. Both gene copies comprised four exons and three introns. The exon lengths for both genes are identical, except for the first exon, which is 15 bp shorter in *LangCHIL*1 compared with *LangCHIL*2. All *LangCHIL*2 intron sequences are longer than those of *LangCHIL*1. The *LangCHIL*1 cDNA sequence is 630 bp, encoding a putative protein of 209 AA, whereas the *LangCHIL*2 cDNA is 645 bp, and expected protein is 214 AA (Figure 1). The amino acid sequences of these two proteins were approximately 92% identical. The two *LangCHIL* sequences were deposited in the EMBL Nucleotide Sequence Databank under accession numbers HE999614 (*LangCHIL*1) and HE999615 (*LangCHIL*2).

### Genetic mapping of *LangCHIL* genes

PCR primers flanking the almost full-length coding sequences of the 28O01 and 5L11 *LangCHIL* sequences were designed. PCR products, amplified from the DNA of parental lines of the *L. angustifolius* mapping population, were sequenced. In both genes, sequence differences between the parental lines were detected in the 3rd exon: a 9-bp length polymorphism in *LangCHIL*1 and a SNP in *LangCHIL*2. Segregation of the length polymorphism was visualized by PCR, and the SNP by dCAPS. Based on the segregation patterns (Supplementary Table 8), CHIL1 marker was mapped in linkage group NLL-03 of the *L. angustifolius* genetic map, whereas CHIL2 was localized in NLL-15 (Table 2).

**Table 2:** ***LangCHIL* gene-based molecular marker characteristics**.

**Marker name**	**Marker type**	**Enzyme**	**83A:476 product length (bp)**	**P27255 product length (bp)**	**LOD scores**	Linkage group^*^	**GenBank accession**
CHIL1	Co-dominant (PCR)	–	320	329	15.4, 18.6	3	GF112056
CHIL2	Co-dominant (dCAPS)	*Taq*I	340	310, 30	22.6, 22.9	15	GF112057

LOD, logarithm of odds.

* Linkage group assignment according to cicki, Kroc et al. (2014).

### Cytogenetic mapping of *LangCHIL* genes

**Figure 2:**
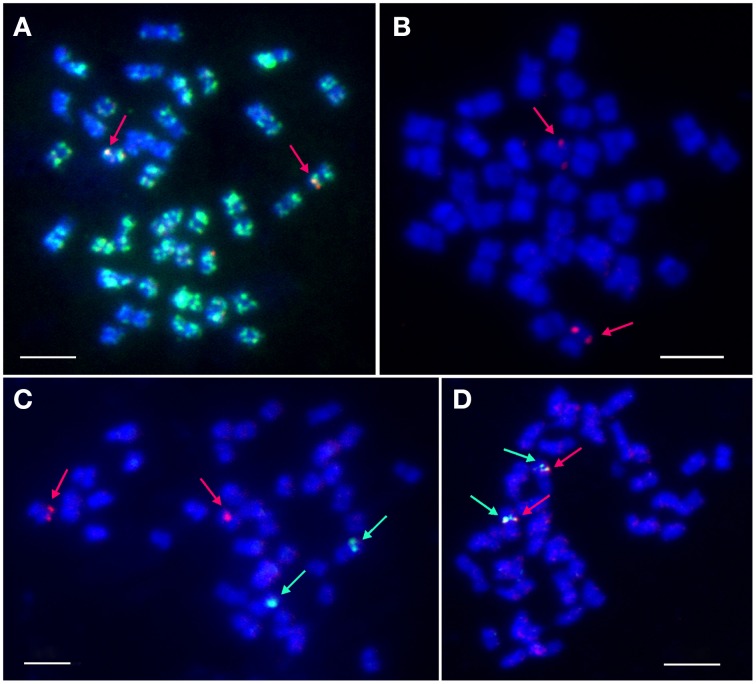
**Localization of selected BAC clones in *L. angustifolius* mitotic chromosomes using FISH**. **(A)** 5L11 (red signals) and 106M03 (green signals); **(B)** 88J04 (red); **(C)** 28O04 (green) and 134F01 (red); **(D)** 115L05 (green), and 115N04 (red). Arrows indicate single-locus signals. DNA of the BAC clones was labeled with tetramethylrhodamine-5-dUTP (red) or digoxigenin-11-dUPT (green). Overlapping BACs produced yellow signals. Chromosomes were counterstained with DAPI (blue). Bars: 5 μm.

BAC-FISH was performed to localize and identify the number of *LangCHIL* loci in the mitotic chromosomes of *L. angustifolius*. Various combinations of eight clones belonging to both contigs were used as molecular probes. BAC clones originating from the same contig gave overlapping single-locus signals on one pair of homologous chromosomes, whereas those from different contigs showed single locus signals on two different chromosome pairs (Figures 2A,B). Two clones from contig 1 (106M03, 129C12) and one singleton (41I07) produced dispersed signals (Supplementary Table 9). The cytogenetic locations of selected BAC clones are shown on Figures 2C,D.

BAC clones hybridizing to a single locus on mitotic chromosomes were defined as cytogenetic markers. They constituted, together with CHIL gene-based markers, the cornerstones for further integration of genetic and cytogenetic maps of the narrow-leafed lupin genome. Based on the results of cytogenetic mapping, two new linkage groups were assigned to corresponding chromosomes: NLL-03 (clones 28O01, 88J04, 115L05, 115N04) and NLL-15 (clones 5L11, 134F01).

### Reference to the genome sequence of *L. angustifolius*

BESs of clones originating from both contigs were aligned to the scaffolds of the narrow-leafed lupin draft genome sequence (Yang et al., 2013), where they tagged 12 sequences: nine for contig 1 and three for contig 2 (Supplementary Table 10). The total length of selected scaffolds was 187084 nt. The orientations of seven scaffolds were determined by locating two or more paired BESs. The sequences of the scaffolds were functionally annotated to supplement the information obtained from the *in silico* analysis of the BESs and *LangCHIL* genes. Repeatmasker survey (Supplementary Table 11) revealed that the sequences of scaffolds anchored to contig 1 had a higher percentage of coverage by repetitive elements than those mapped to contig 2 (24.5 vs. 14.9%). Such a difference in repeat content reflected mainly an accumulation of Copia retrotranspozons in contig 1. However, representatives of transpozon classes Helitron and hAT elements were identified only in contig 2 (Supplementary Table 12). BESs of the clones overlapping with BACs 28O01 and 5L11 were mapped to the main scaffolds from both contigs (contig 1: KB421708.1 and KB412128.1, contig 2: KB425167.1 and KB430490.1). All clones but 115N04 were localized. Obtained results supported, in general, the postulated arrangement of BACs in the contigs, as inferred from the restriction analyses (Supplementary Figure 3).

### Phylogenetic survey of *L. angustifolius* CHI-fold proteins

**Figure 3:**
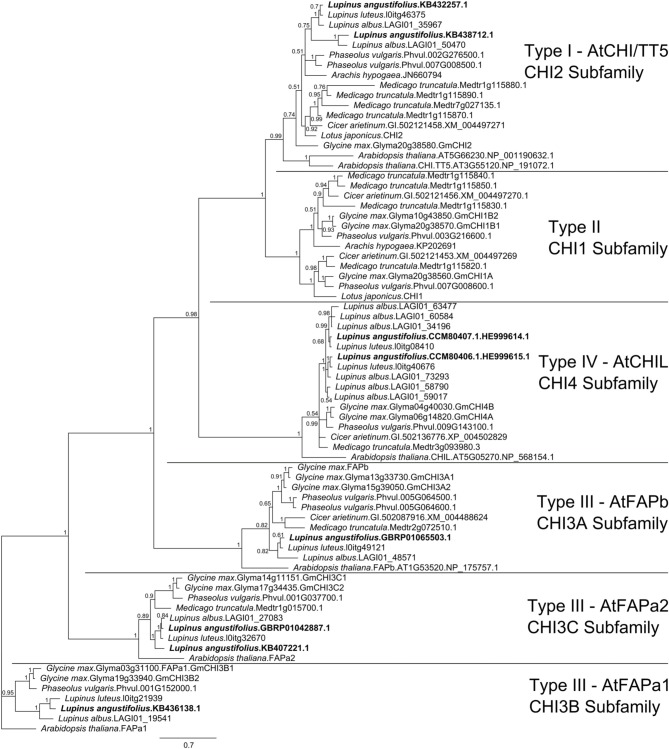
**Majority rule consensus of 4002 trees found in a Bayesian analysis of 8 *L. angustifolius* and 66 reference CHI-fold protein sequences. Numbers are posterior probabilities**. 480 amino acid positions were included in the final alignment used for calculating the tree.

The narrow-leafed lupin genome and transcriptome were screened with 172 CHI-fold reference protein sequences (Supplementary Table 2) belonging to six main subfamilies (CHI1, AtCHI/TT5/CHI2, AtFAPb/CHI3A, ATFAPa1/CHI3B, AtFAPa2/CHI3C, AtCHIL/CHI4). Mining of the *L. albus* transcriptome resulted in identification of 20 CHI-fold like sequences but multiple alignment survey revealed that 5 were redundant and 4 truncated. In the *L. luteus* trancriptome 7 sequences were selected, however one was truncated. Together with sequences obtained in this study, eight copies of *L. angustifolius*, 11 *L. albus* and six *L. luteus* CHI-fold genes were identified (Supplementary Table 3). The Bayesian phylogenetic inference of these sequences and selected 48 reference CHI-fold proteins revealed that in the *L. angustifolius* genome representatives of all but one main CHI subfamilies are present (Figure 3). They are as follows: CHI2, KB438712.1 and KB432257.1; CHI3A, GBRP01065503.1; CHI3B, KB436138.1; CHI3C, GBRP01042887.1 and KB407221.1 as well as CHI4, HE999615.1 and HE999614.1; sequences revealed in this study (Table 3).

**Table 3:** ***L. angustifolius* genome and transcriptome sequences mapped to the CHI-fold protein family genes**.

**CHI-fold subfamily genes**	***L. angustifolius* genome scaffold**	***L. angustifolius* transcriptome**	**% identity**	**Total alignment length**	**Total score**	***E*-value**
CHI2 copy 1	KB438712.1	GBRP01036913.1	100.00	679	1255	0.0
CHI2 copy 2	KB432257.1	GBRP01007050.1	100.00	1147	2119	0.0
LangCHIL1	KB421708.1	GBRP01001161.1	99.90	1027	1893	0.0
	HE999614.1	GBRP01001161.1	99.71	1028	2518	0.0
LangCHIL2	KB430490.1	–	–	–	–	–
	HE999615.1	–	–	–	–	–
CHI3A copy	–	GBRP01065503.1	–	–	–	–
CHI3B copy	KB436138.1	GBRP01030694.1	100.00	942	1742	0.0
CHI3C copy 1	KB407221.1	–	–	–	–	–
CHI3C copy 2	–	GBRP01042887.1	–	–	–	–

### Insight into cross-genera synteny of genomic regions neighboring CHI-fold protein genes

*L. angustifolius LangCHIL* scaffold sequences were aligned to the genome sequences of six legume species to identify blocks of conserved collinearity among diverse lineages of Papilionoid clade. Conserved syntenic patterns were observed for four scaffolds: 39988 (KB421708.1) and 20923 (KB412128.1) from contig 1, and 48639 (KB425167.1) and 65565 (KB430490.1) from contig 2. To enrich the information resulting from cross-genera genome alignments, sequences of those four scaffolds were comparatively mapped against the transcriptome sequences of yellow lupin (*L. luteus*) (Parra-González et al., 2012) and white lupin (*L. albus*) (O'Rourke et al., 2013). Numerous alignments to ESTs originating from both species were identified in all analyzed scaffolds (Supplementary Table 13). Based on the distribution of mapped ESTs over the scaffold sequences, six hypothetical genes in contig 1 and four in contig 2 were identified. Taking into consideration the physical lengths of the scaffolds, the gene densities were calculated as follows: 10.6 genes per 100 kb in contig 1 and 9.9 genes per 100 kb in contig 2. Thus, both contigs represent gene-rich regions. These regions differ considerably in the functions of their encoded genes: the only link is the presence of a *LangCHIL* gene copy, as shown in Table 4.

**Table 4:** **Genes identified in scaffolds showing conserved synteny to model legumes**.

***LangCHIL* contig No.**	**Gene No**.	**Scaffold accession**	**Scaffold sequence start**	**Scaffold sequence end**	**Gene name**	**Reference sequence accession**	**Sequence identity**
1	1	KB421708.1	1940	3633	Chalcone isomerase-like	NM_001249853.1	663/815 (81%)
1	2	KB421708.1	5599	7758	–	–	–
1	3	KB421708.1	15760	24106	Peroxisomal fatty acid beta-oxidation multifunctional protein	XM_004512927.1	1815/2157 (84%)
1	4	KB412128.1	13083	17912	Transcription factor RF2b	XM_004502774.1	1207/1609 (75%)
1	5	KB412128.1	24944	26655	Expansin-A	XM_003523228.1	934/1156 (81%)
1	6	KB412128.1	29902	29565	3-ketoacyl-CoA synthase	XM_003555308.1	269/349 (77%)
2	7	KB425167.1	2127	7552	Methionyl-tRNA synthetase	XM_003526754.1	1073/1245 (86%)
2	8	KB430490.1	1721	5297	U-box domain-containing protein	XM_003522461.1	1094/1357 (81%)
2	9	KB430490.1	7703	9623	Chalcone isomerase-like	NM_001249853.1	658/803 (82%)
2	10	KB430490.1	14686	15974	RING-H2 finger protein	XM_003523230.1	839/1113 (75%)

**Figure 4:**
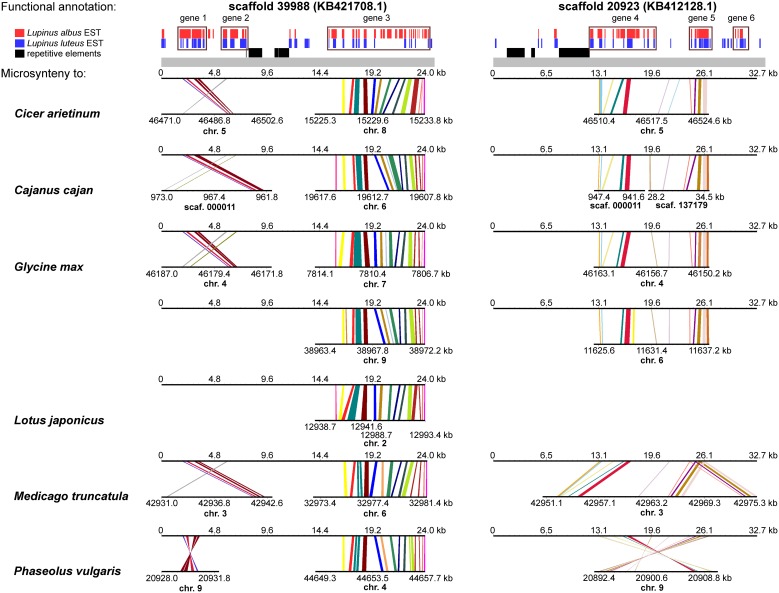
**Functional annotation and microsyntenic links of two *L. angustifolius* scaffolds from contig 1 carrying the *LangCHIL*1 gene**. Functional annotations of two scaffolds are presented on upper plot above the gray bar. Black bars indicate repetitive elements, whereas blue and red correspond to *L. luteus* and *L. albus* transcriptome reads, respectively (Parra-González et al., 2012; O'Rourke et al., 2013). Rectangles with numbers 1–6, which refer to annotation data presented in the Table 4, mark predicted genes. Microsyntenic links between the scaffolds and genomic sequences of six legume species are shown on Genome Synteny Viewer diagrams (Revanna et al., 2011), below the gray bar. Each diagram comprises two horizontal lines. The upper line shows the sequence of *L. angustifolius* scaffold: 39988 (KB421708.1) on the left column and 20923 (KB412128.1) on the right. The lower line shows the corresponding region of a model legume genome. The scales of the bottom bars vary by species, thus the chromosome localizations (kb) are given. Homologous links are colored consecutively to portray the order and orientation of syntenic blocks. *LangCHIL*1 gene is located in KB421708.1 from 1940 to 3633 bp.

**Figure 5:**
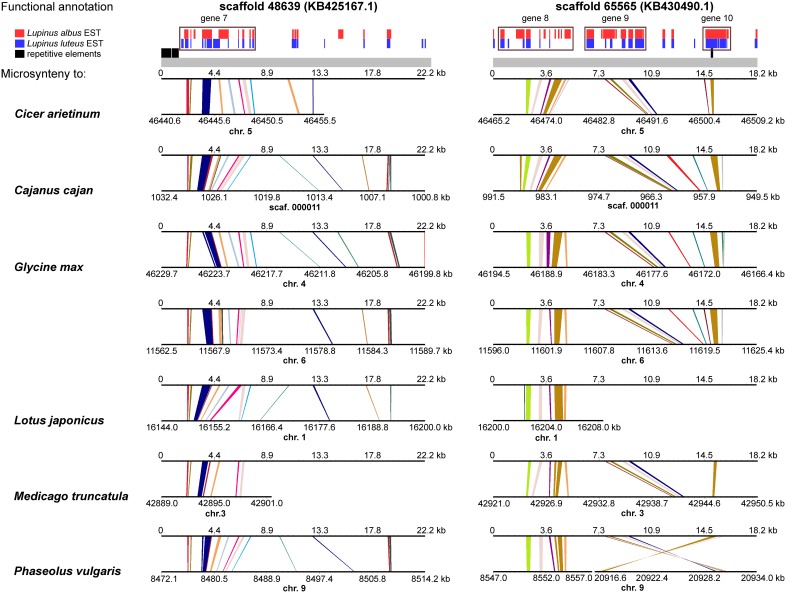
**Functional annotation and microsyntenic links of two *L. angustifolius* scaffolds from contig 2 carrying the *LangCHIL*2 gene**. The functional annotations of two scaffolds are presented on upper plot above the gray bar. Black bars indicate repetitive elements, whereas blue and red correspond to *L. luteus* and *L. albus* transcriptome reads, respectively (Parra-González et al., 2012; O'Rourke et al., 2013). Rectangles with numbers 7–10, which refer to annotation data presented in the Table 4, mark predicted genes. Microsyntenic links between the scaffolds and genomic sequences of six legume species are shown on Genome Synteny Viewer diagrams (Revanna et al., 2011), below the gray bar. Each diagram comprises two horizontal lines. The upper line shows the sequence of *L. angustifolius* scaffold: 48639 (KB425167.1) on the left column and 65565 (KB430490.1) on the right. The lower line shows the corresponding region of a model legume genome. The scales of the bottom bars vary by species, thus the chromosome localizations (kb) are given. Homologs links are colored consecutively to portray the order and orientation of the syntenic blocks. To simplify the illustration, scaffold 48639 is presented as the reverse-complement sequence. *LangCHIL*2 gene is located in KB430490.1 from 7703 to 9623 bp.

A comprehensive survey of syntenic relationships, with reference to the sequence annotation data, revealed that studied *L. angustifolius* scaffolds exhibited lower numbers of sequence collinearity links to *M. truncatula* and *L. japonicus* than to *C. arietinum*, *C. cajan*, *G. max*, and *P. vulgaris*. Importantly, the structure of contig 2 was much more conserved among the analyzed species than that of contig 1 (Figures 4, 5). For this contig, carbon copies of quasi-ancestral sequence of *L. angustifolius* scaffolds 48639 and 65565 were distinguished in *C. cajan* scaffold 000011, *G. max* chromosomes 4 and 6 (whole region duplication), and *P. vulgaris* chromosome 9. The partially retracted arrangement was observed in the corresponding sections of *C. arietinum* chromosome 5, *L. japonicus* chromosome 1, and *M. truncatula* chromosome 3. The collinearity of the sequence alignments of the region encoding *LangCHIL*2 was almost perfectly preserved among four species: C. *arietinum*, *C. cajan*, *G. max*, and *M. truncatula*. In *P. vulgaris*, a sequence inversion and a large insertion or deletion (12.36 Mbp) were detected; however, the *LangCHIL* gene structure remained unaffected. The *L. japonicus* genomic sequence, however, revealed no synteny to the region of *LangCHIL*2.

Contig 1, represented by scaffolds 39988 and 20923, has accumulated more structural modifications during evolution than contig 2. In general, three regions in this contig, having distinct homology links, were identified: the 5′ part of scaffold 39988, the middle section of scaffold 20923, and the 3′ part of scaffold 39988 (listed from the most to the least conserved). The 5′ part of scaffold 39988, carrying *LangCHIL*1, was mapped to the same loci in *C. arietinum*, *C. cajan*, *G. max*, *M. truncatula*, and *P. vulgaris* genomes as the *LangCHIL*2 region. This implied that both copies of the *LangCHIL* genes present in the narrow-leafed lupin genome correspond to one sequence of this gene in the other species. Moreover, when the coding sequences of both *LangCHIL* genes where BLAST searched against the annotated soybean genome (Supplementary Table 14), they revealed the highest sequence similarity to *CHIL* transcript Glyma06g14820 on chromosome 6 (at 11 616 kb). The second strongest match obtained was *CHIL* transcript Glyma04g40030 located at chromosome 4 (at 46 176 kb).

Scaffolds carrying the remaining CHI-fold protein genes were also comparatively mapped to selected legume genomes (Supplementary Figure 4). Results of this analysis perfectly converged with those of Bayesian assay. Scaffold KB436138.1, carrying hypothetical FAPa1 gene, revealed, for all analyzed legume species, microsynteny links to genome regions containing FAPa1 subfamily sequences, including those already annotated: *C. arietinum* locus Ca3: 30652402-30657185, *G. max* Glyma03g31100 and Glyma19g33940, *M. truncatula* Medtr7g094980.1, and *P. vulgaris* Phvul.001G152000. Similarly, scaffold KB407221.1, encoding hypothetical FAPa2 sequence, showed homology links to legume genome loci carrying FAPa2 subfamily genes, like: *C. arietinum* locus Ca4: 44847754-44853436, *G. max* Glyma14g11151 and Glyma17g34435, *M. truncatula* Medtr1g015700, and *P. vulgaris* Phvul.001G037700. Analogously, scaffolds KB432257.1 and KB438712.1, containing hypothetical *CHI*2 subfamily sequence, revealed microsyntenic pattern to regions surrounding or adjacent to *CHI*2 loci. These included *C. arietinum* XM_004497271, *G. max* Glyma20g38580, *M. truncatula* Medtr1g115870 and Medtr1g115880, and *P. vulgaris* Phvul.007G008500.

### Localization of *LangCHIL* genes in large macrosyntenic blocks

To determine whether the identified microsyntenic links were representatives of longer regions of preserved gene collinearity (i.e., macrosynteny), comparative mapping of the *M. truncatula*, *L. japonicus*, *C. arietinum*, *P. vulgaris*, and *C. cajan* genomes to the *G. max* genome was performed. All microsyntenic loci assigned in these genomes for the analyzed *LangCHIL* scaffolds were anchored in large conserved blocks, spanning several megabase pairs and containing many genes. The mean lengths of the syntenic blocks shared between *G. max* and the analyzed species were: *M. truncatula*, 4.2 Mb; *P. vulgaris*, 4.0 Mb; *C. arietinum* and *L. japonicus*, 2.5 Mb; *C. cajan*, 0.3 Mb. The outlier calculated for *C. cajan* was mainly caused by fragmentation of the genome sequence (incompleteness of the sequencing project), resulting in the localization of syntenic loci in short unlinked scaffolds. The coordinates and lengths of genome sequence blocks conserved between *G. max* and the analyzed legume species, in regions with syntenic links anchored to the studied *L. angustifolius* contigs, are shown in Supplementary Table 15.

To determine if the identified conserved blocks are landmarks of chromosome-scale macrosyntenic relationships, marker sequences from *L. angustifolius* linkage group NLL-03 and NLL-15 were BLAST searched against sequences of six legume genomes. Genomes of *L. japonicus* and *C. cajan* revealed homology links dispersed on numerous chromosomes and scaffolds, apparently caused by the high ratio of genome data fragmentation. For the remaining four genomes, significant arrays of macrosynteny were observed (Supplementary Figure 5). Molecular markers from NLL-15 formed macrosyntenic blocks in two chromosomes of *G. max* (chr. 4 and 6) and in single chromosomes of: *C. arietinum* (chr. 5), *M. truncatula* (chr. 3), and *P. vulgaris* (chr. 9). In all syntenic chromosome regions but *G. max* chr. 6, large inversions spanning several Mbp were observed. *LangCHIL*2 locus was located in the region showing conserved macrosynteny, visualized by the preserved order of adjacent markers. However, in the vicinity of the *LangCHIL*2 locus in *P. vulgaris* chr. 9 and *C. arietinum* chr. 5, inversion of a block containing a few markers was observed.

Patterns of macrosyntenic relationships detected for linkage group NLL-03 were more complex than those of NLL-15. Sequence-defined markers from NLL-03 revealed homology links to fragments of three chromosomes of *C. arietinum* and *P. vulgaris*, five of *M. truncatula* and seven of *G. max*. Those included chromosomes having syntenic links to markers from NLL-15. Besides the fragmentation of syntenic blocks, numerous local inversions of NLL-03 marker orders were observed within regions of shared collinearity. Duplications of syntenic regions harboring several markers were identified in the genomes of *M. truncatula* (chr. 3 and 5, chr. 2 and 8), *P. vulgaris* (chr. 1 and 9), and *G. max* (chr. 12 and 13). Moreover, in the genome of *G. max*, one quadruplication of the syntenic region was observed (chr. 4, 6, 14, and 17). Note that despite many rearrangements and duplications of syntenic regions in the analyzed legume species, the *LangCHIL*1 locus, together with few surrounding markers, revealed sequence homology links to the same chromosomes as those matching *LangCHIL*2. Syntenic relationships to the other chromosomes mentioned above were detected mostly for markers mapped in NLL-03 quite distant from *LangCHIL*1. The sequence of *G. max* chr. 9 which showed well preserved local sequence collinearity (Supplementary Table 15) did not reveal the minimum number of homology links to be considered as a macrosyntenic block. It may indicate that the conserved region observed at this chromosome is relatively short.

### Organ-specific *CHI-like* gene expression analysis

**Figure 6:**
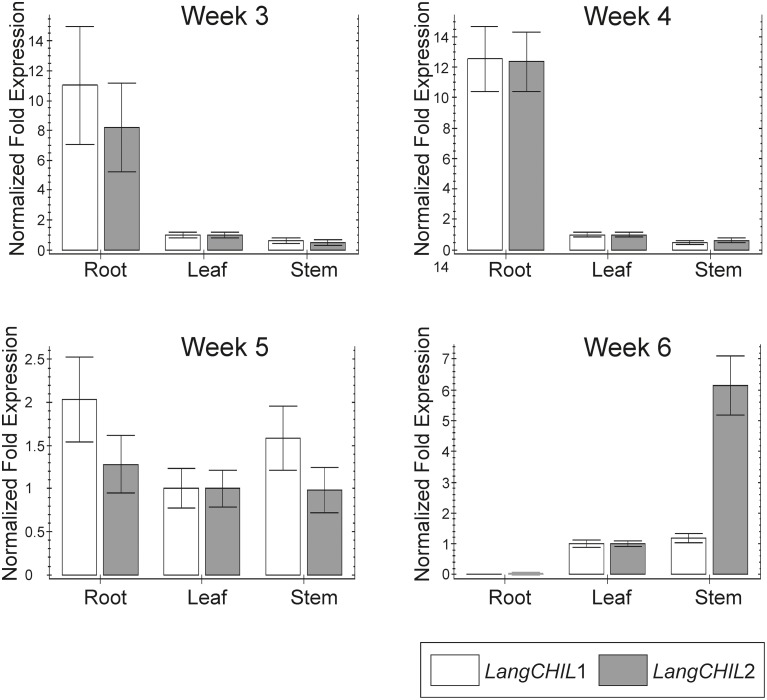
**Organ-specific expression of *L. angustifolius LangCHIL* genes**. The samples were collected from 3-, 4-, 5-, and 6-week-old leaves (L), stems (S), and roots (R). Transcript levels were analyzed by qRT-PCR. Transcript levels are given as relative values to L (the value of 1), after being normalized to the 18S ribosomal RNA and β-tubulin levels. Mean values are shown and the bars represent ± SD from three independent experiments.

Expression profiles of *LangCHIL*1 and *LangCHIL*2 genes in roots leaves and stems were determined by real-time PCR at four time points spanning the growing period from 3 to 6 weeks after sowing. The presence of both *LangCHIL* gene transcripts was revealed in all examined lupin samples; however, their expression patterns were different in distinct organs (Figure 6). In 3-week-old plants, the expression levels of both genes in stems were somewhat lower than in leaves, whereas expression levels in the roots were 8 (*LangCHIL*2) to 11 (*LangCHIL*1) fold higher than in leaves. The expression levels of transcripts in the 4-week old roots leveled off and were 12-fold higher than in the leaves, while expression in stems remained at a constant low level. In 5-week old plants, the expression levels of *LangCHIL*1 and *LangCHIL*2 were moderately reduced in the roots, and therefore, tended to equalize in all organs. We observed an increase of *LangCHIL*1 gene expression in stems; however, the highest level of *LangCHIL*1 gene expression was still observed in the roots. In 6-week-old plants, the expression patterns of these two *LangCHIL* genes were distinct: the level of *LangCHIL*2 transcripts dramatically decreased in roots and strongly increased in stems, whereas the *LangCHIL*1 gene expression level in stems decreased to the level of the control.

## Discussion

### Chalcone isomerase-like gene number and structure

In the present study, we used a white lupin *CHIL* EST sequence as a probe to identify *CHIL* genes in BAC genomic clones of the narrow leaf lupin. Genomic BAC libraries have been used for phenylpropanoid biosynthesis gene detection in several species: *G. max* (Tuteja and Vodkin, 2008) *Vitis vinifera* (Tomkins et al., 2001), *Sorghum bicolor* (Lo et al., 2002), and *Eucalyptus grandis* (Paiva et al., 2011). EST probes are specific and produce reliable results, generating a small number of positive hybridization signals that directly reflects the gene copy number and genome coverage in the library (Shopinski et al., 2006). They were probes of choice for *G. max* (Shopinski et al., 2006), *Brachypodium distachyon* (Huo et al., 2006), *Salmo salar* (Thorsen et al., 2005), and *Phytophthora nicotianae* (Shah and Hassan, 2005) analysis. However, this method has some limitations, revealed in our study. The *CHIL* probe from *L. albus* did not hybridize to *CHI1* and *CHI2* genes or the more distantly related *FAP* paralogs present in the genome of *L. angustifolius*. It was due to the rather low sequence identity between these genes. The *CHIL* probe from *L. albus* (CA410672) appeared to be more than 95% identical to the corresponding sequence in exon 2 of *LangCHIL*1. The sequence of the closely related *G. max CHIL* gene *CHI4A* (Glyma06g14820) is ~89% identical to the *L. albus* probe. In the region corresponding to the 200 nt probe from *L. albus*, the overall identity between soybean *CHI4A* and *CHI2* (Glyma20g38580) genes is only ~42%.

The EST probe identified 10 BAC clones. The genome coverage of the library was estimated as six-fold (Kasprzak et al., 2006); therefore, number of clones indicated the presence of two copies of *LangCHIL* in the genome. To identify clones carrying overlapping sequences, the physical mapping based on restriction fingerprinting was performed; such an approach was helpful in selecting clones for sequencing (Książkiewicz et al., 2013) as well as in filling in missing portions of the already sequenced genomes (Marra et al., 1997; Meyers et al., 2004). The physical mapping of the BACs identified two singletons and allowed the construction of two contigs: one consisting of six clones and the second of two clones. One clone from every contig, characterized by a single locus BAC-FISH signal, was selected for sequencing. Our results demonstrated that there are two distinct *LangCHIL* gene copies in the narrow-leafed lupin genome. One of the two BACs which showed false-positive hybridization signals was 12M14. Putatively, these signals resulted from high occupancy of simple repeats because this clone revealed positive signals in BAC library screening approach with SSR-based probes. Moreover, 12M14 clone was mapped in linkage group NLL-11, other than any of *LangCHIL* markers (Książkiewicz et al., 2015).

The number of *CHI* genes depends on the species. It has been generally believed that in non-legume species, a single gene encodes CHI (Grotewold and Peterson, 1994; Durbin et al., 2003; Li et al., 2006; Kim et al., 2007; Cheng et al., 2011). Earlier papers reported that in legumes, the number of *CHI* varies from one in *M. truncatula* by two in *M. sativa*, and *Glycyrrhiza echinata*, up to four in *L. japonicus*, and five in *G. max* (McKhann and Hirsch, 1994; Kimura et al., 2001; Shimada et al., 2003; Ralston et al., 2005). However, whole genome sequencing followed by annotation and phylogenetic analyses, revealed increasing numbers of predicted *CHI* genes and led to subdivision of CHI-fold protein family into subfamilies: CHI (encompassing CHI1 and CHI2), CHIL, and FAP (comprising FAPa1, FAPa2 and FAPa3) (Ngaki et al., 2012; Chu et al., 2014). Recent studies revealed that the numbers of CHI-fold protein gene copies in legumes are as follows: five in *C. arietinum*, seven in *P. vulgaris*, nine in *M. truncatula*, and 12 in *G. max* (Chu et al., 2014; Dastmalchi and Dhaubhadel, 2015). The identification of two *LangCHIL* genes in the narrow-leafed lupin genome is compatible with two GmCHI4 (A and B) from *Glycine max* (Dastmalchi and Dhaubhadel, 2015) as well as so called *EFP* (*enhancer of flavonoids production*) genes identified in Japanese morning glory (Morita et al., 2014).

Most plant *CHIs* have four exons and three introns located in conserved positions (Van Tunen et al., 1988; Kuittinen and Aguadé, 2000; Druka et al., 2003; Shimada et al., 2003; Kim et al., 2004). Both *L. angustifolius LangCHIL* genes also comprise four exons and three introns. However, some species have fewer exons and introns in their *CHIs*: three in *Hordeum vulgare* (Druka et al., 2003) and *Ginkgo biloba* (Cheng et al., 2011) or even one in *CHIB* in *Petunia hybrida* (Van Tunen et al., 1988). It was suggested that the four-intron structure represented the ancestral *CHI*, and further independent intron loss events during its evolution had occurred in distinct plant species (Shoeva et al., 2014). The lengths of coding and non-coding sequences of the narrow-leafed lupin *LangCHIL* genes are similar to those from other species. In *L. japonicus*, lengths of all exons differ by only a few base pairs as compared with *L. angustifolius*, and all four-exon isoenzymes have the same lengths of individual coding sequences (Shimada et al., 2003). The lupin *LangCHIL* exons are similar in length to those of maize and rice (Druka et al., 2003). However, differences were found in the noncoding sequences: the first two introns of lupin *LangCHIL* are several times longer than those in the above-mentioned species. The coding sequences of the two *LangCHIL* homologs are about 93% identical to each another.

Moreover, both analyzed narrow-leafed lupin *CHIL* genes have the highest similarity to *CHI4* from *G. max*, with nucleotide identities of 91 and 88% for *LangCHIL*2 and *LangCHIL*1 genes, respectively. Such values are similar to those presented in recent sequence homology studies of other legumes. An evolutionary survey of the isoflavonoid pathway genes, including the *G. max CHIL* homologs from chromosome segments highly syntenic to *LangCHIL* coding regions, revealed a low rate of sequence polymorphism among studied legume gene copies (Chu et al., 2014). Reported percentage substitution values varied from 3 to 12% (~3% between two soybean copies Glyma04g40030 and Glyma06g14820, ~7% between those copies and *P. vulgaris* transcript Phvul.009G143100, and ~12% between the latter sequences and *M. truncatula* homolog Medtr3g093980). Furthermore, a phylogenetic analysis of 19 *CHI* homologs from 16 species showed high sequence similarity between representatives of legumes (Zhang et al., 2012). The values of substitution per site between *CHI*1 gene copies from different species (calculated from the phylogenetic tree) were: ca. 6% between *P. vulgaris* and *G. max*, ca. 14% between *Pisum sativum*, *M. sativa* and the *Phaseolus-Glycine* clade, and ca. 15% between the branches mentioned and the *L. japonicus* lineage. Such a comparison shows that levels of amino-acid sequence conservation in CHIL and CHI1 subfamilies are very similar.

### *LangCHIL* location in linkage groups of the *L. angustifolius* genetic map

Genetic mapping of phenylpropanoid pathway genes was successful for 19 different genes in *P. vulgaris* (Yadegari and Pauls, 2008), two genes in *Cynara cardunculus* (Comino et al., 2007), the *PAL* gene in *Coffea canephora* (Mahesh et al., 2006), and two *CHS* genes in *Lycopersicon lycopersicum* (O'Neill et al., 1990).

The CHIL1 and CHIL2 markers were located in two (NLL-03 and NLL-15) linkage groups of the recently published, narrow-leafed lupin genetic map (Kroc et al., 2014). The *leucospermus* locus is located at NLL-03, close to CHIL1. The hypothetical *leucospermus* gene controls pigment production in seeds, cotyledons, and flowers. Its recessive form manifests in white flowers, green cotyledons, and almost white seeds of cultivated forms. It was found that the *Arabidopsis CHI* is responsible for the seed coat color (Kim et al., 2007). That observation, together with close linkage of CHIL1 and *leucospermus*, suggests that *LangCHIL* might confer the *leucospermus* trait.

In contrast to *L. angustifolius*, in *L. japonicus*, four *CHI* genes are arranged as a cluster of tandemly aligned sequences (Shimada et al., 2003). Such differences suggest different origins of the gene copies. Instead of ancestral gene duplication, typical for secondary metabolism pathways (Ober, 2010), a copy of *LangCHIL* could arise by duplication of the entire genome during the paleopoliploidization process, which is probably responsible for the formation of contemporary lupin species (Atkins et al., 1998; Cannon et al., 2010).

### Cytogenetic localization of *LangCHIL* in the *L. angustifolius* genome: a step toward integration of chromosome and linkage maps

BAC-FISH was used to identify the number of *LangCHIL* gene *loci* in the mitotic chromosomes of *L. angustifolius*. Such an approach has been used for gene localization in numerous plant species, including *O. sativa* and *Triticum aestivum* (Qi et al., 2009), *Brassica napus* (Feng et al., 2009), *S. bicolor* (Kim et al., 2002), *A. thaliana* (Fransz et al., 2000), and *Raphanus sativus* (Niemelä et al., 2012). Cytogenetic mapping showed that the two *L. angustifolius LangCHIL* genes are located in different chromosomes. By comparison, in maize, *CHIs* were located at three different loci, while in barley and rice, only one *CHI* locus was detected (Druka et al., 2003). Research concerning the location of other genes of phenylpropanoid pathway, such as phenylalanine ammonia lyase, chalcone synthase, and isoflavone reductase (Narożna et al. unpublished data), is underway. The cytogenetic location of all phenylpropanoid genes would be an important step toward the complete characterization of the flavonoid biosynthetic pathway gene organization in the *L. angustifolius* genome. Moreover, chromosome-specific gene markers are suitable for genetic and cytogenetic map integration. Such an approach has been already used in *M. truncatula* (Kulikova et al., 2001) or *Gossypium hirsutum* (Wang et al., 2007) genome analyses. The integration of the *L. angustifolius* genetic and chromosomal maps is still in progress. To date, 12 chromosomes were assigned by BAC-derived markers to the main linkage groups of the *L. angustifolius* genetic map: NLL-01, NLL-03, NLL-05, NLL-06, NLL-07, NLL-08, NLL-09, NLL-10, NLL-14, NLL-16, NLL-17, and NLL-20, as well as small unlinked cluster-2 (Lesniewska et al., 2011; Książkiewicz et al., 2013, 2015). One of the markers developed in the present work allowed us to assign another linkage group, NLL-15, to a corresponding chromosome. The second marker has been added to the unique chromosomal marker for the linkage group NLL-03, recently published (Książkiewicz et al., 2013).

### The expression pattern of chalcone isomerase-like genes

In the present study, RT-PCR was used to analyze *LangCHIL* genes expression in *L. angustifolius*. Transcripts of both *LangCHIL*1 and *LangCHIL*2 genes were present in all tested samples. The highest level of expression was observed in roots, suggesting that phenylpropanoid metabolism is most active in these organs, which is probably linked with accumulation of flavonoid compounds in the legume roots (Larose et al., 2002). The expression profile of both *LangCHIL* genes in leaves and stems remained at similar low levels. These results are consistent with those of other expression studies performed in legumes on other genes from phenylpropanoid pathway. A relatively high level of *CHI* transcripts in the roots of beans was detected, suggesting that it could be typical for legume plants (Lambais and Mehdy, 1993). In *M. truncatula*, an additional increase in the level of *CHI* transcripts after pathogen attack was observed (Uppalapati et al., 2009). A similar profile of phenylpropanoid pathway gene expression was observed in *M. sativa*: the levels of *CHS* and *CHI* transcripts were highest in young roots and their meristems, lower in developing root nodules inoculated with *Rhizobium meliloti* strains, and very low in the upper parts of the plant (stems, leaves, flowers) (McKhann and Hirsch, 1994). Correspondingly, study on organ-specific expression of the *CHS* gene family in *P. vulgaris* revealed the highest expression of the two isoforms of *CHS* genes in the roots (Dubery and Mienie, 2001). As the authors suggested, it could be related to the role of flavonoids in nodule development, as signal molecules during interactions with rhizobia. Recently, it was concluded that *CHILs* are enhancers of flavonoid production and flower pigmentation, and their function is conserved among diverse land plant species (Fujino et al., 2014; Morita et al., 2014).

Gene expression analysis was also conducted during plant growth. After 4 weeks of growth, *LangCHIL* gene expression reached its highest level, decreased, and then remained at a constant level for the next 2 weeks. Similarly, the highest level of *CHI* transcription was observed in young roots, after which the level fell dramatically and remained relatively stable until the end of the experiment (McKhann and Hirsch, 1994). The level of narrow-leafed lupin *LangCHIL* transcripts isolated from stems and leaves increased during plant development. Similarly, when the expression of another phenylpropanoid biosynthesis key enzyme, anthocyanidin synthase was analyzed in *Ipomea batatas*, the highest initial expression was in the roots, after which expression increased in stems, leaves, and flowers (Liu et al., 2010). The authors indicated that *CHI* is a tissue-specific regulated gene and the accumulation of anthocyanins correlated with the level of its expression. The highest level of transcript in the roots corresponds to the initial synthesis and accumulation of anthocyanins. After synthesis in the roots, they are transported to the stems, leaves, and other tissues and organs, which correlated with a decrease in expression in the roots and a simultaneous increase in the other plant tissues. The expression of *CHIs* in plants changes depending on organ/tissue and/or development stage. In various species, it affects the flavonoid content, influencing the color of specific organs. Thus, in tomato, *CHI* affects the pericarp and pulp color in fruits (Muir et al., 2001). In onion, it influences bulb color (Kim et al., 2004), while in tobacco it controls flower color (Nishihara et al., 2005). In *Arabidopsis*, *CHI* gene influences the seed coat color (Kim et al., 2007).

The transcript profiles of both *LangCHIL* isoforms in narrow-leafed lupin were very similar; the only differences were a higher level of *LangCHIL*1 in 3- and 5-week-old roots and a significantly higher *LangCHIL*2 level in 6-week-old stems comparing to control. Similarly, it was revealed that two of the three isoforms of phenylalanine ammonia-lyase (PAL) genes in coffee had the same expression pattern (Lepelley et al., 2012). The authors suggested that these two copies arose by intergenomic duplication, whereas the third *PAL* isoform, characterized by increased expression in other organs, was formed by small translocations, not detected during the global mapping of the genome (Lepelley et al., 2012). Therefore, the similarity of the expression patterns of both *LangCHIL* genes in *L. angustifolius* suggested that they also arose by ancestral gene duplication. Moreover, the high similarity of amino acid sequences and the same exon and intron numbers, indicated that the gene duplication process occurred relatively recently. Considering the location of *LangCHIL* genes in the *L. angustifolius* genome in different chromosomes, it was concluded that these two copies of the gene had appeared as a result of partial or whole genome duplication during the paleoploidization process. Despite the well documented transcriptional activity, the functional role of CHIL protein remains not clear. The CHIL (CHI4) protein from *Glycine max* encoded by *Gma4A* metabolized chalcones neither *in vitro* nor in *E. coli*, suggesting that it is not a functional chalcone isomerase (Ralston et al., 2005). However, studies on fungal and bacterial species revealed that CHIL proteins putatively retain enzymatic activity even in the absence of conserved tyrosine, but the substrate is unknown, and is unlikely to be chalcone (Gensheimer and Mushegian, 2004). Moreover, CHIL from *A. thaliana* (AT5G05270) has been shown to be functional in *E. coli* (Koopman et al., 2012).

### Evolutionary characteristics of *L. angustifolius* chalcone isomerase-fold gene copies

The numbers of CHI-fold protein ancestral genes in the most recent common ancestor (MRCA) of dicots and monocots, and of dicots and that of legumes were estimated as three, three, and five, respectively (Chu et al., 2014). Ancestral CHI-fold protein gene copies were retained during the evolution of particular plant species and, indeed, were subjected to ancient tandem and recent segmental duplications, which increased the number of extant copies to five in *A. thaliana*, eight in *G. max*, or even nine in *M. truncatula* (Chu et al., 2014). The present study revealed that in the *L. angustifolius* genome, two copies of the *CHI2*, *CHIL*, and *FAPa2* gene are present, but only one *FAPa1* and *FAPb*, and no single *CHI1*. The question arises as to which legume ancestral *CHI*-fold copy(-ies) has(-ve) been lost during the evolution of the *Lupinus* genus. Are the two extant copies of *CHI2*, *CHIL*, and *FAPa2* direct descendants of their ancestor genes or remnants of ancient duplications? Taking into consideration that as many as four *CHI1* copies exist in *M. truncatula* genome and at least three in *G. max*, why no single *CHI1* sequence was detected in the narrow-leafed lupin? The last question will remain unanswered, however, the two preceding ones can be addressed.

Briefly, micro- and macrosyntenic analyses of *LangCHIL* harboring scaffolds and adjacent molecular markers from the linkage map showed that the most conserved relationships of collinearity refers to the regions located at *G. max* chromosomes 4 and 6, *C. arietinum* chr. 5, *M. truncatula* chr. 3, and *P. vulgaris* chr. 9. All those regions contain a copy of *CHIL* gene, as follows: Glyma04g40030, Glyma06g14820, GI:502136776 (XM_004502772), Medtr3g093980, Phvul.009G143100. Those copies fall into one monophyletic group, as demonstrated by a comprehensive phylogenetic survey of 47 CHI-fold protein sequences derived from seven species (*A. thaliana*, *O. sativa*, *V. vinifera*, *M. truncatula*, *G. max*, *P. vulgaris*, and *C. arietinum*) (Chu et al., 2014). This phylogenetic branch includes, besides those five sequences, AT5G05270 from *A. thaliana*, Os12g02370 and Os11g02440 from *O. sativa*, and GSVIVT01032685001 from *V. vinifera*; thus, the branch has at least one copy of *CHIL* from every analyzed species (Chu et al., 2014). Such a pattern of microsyntenic and phylogenetic relationships indicates that a single copy of *CHIL* was present at the early stage of the *Lupinus* lineage evolution. Then, after divergence of the branch leading to the *Lupinus* genus, a copy of *CHIL* gene present in contig 1 (*LangCHIL*1) might have appeared as a result of duplication of the chromosomal segment harboring several genes, including *CHI2*. Subsequently, this event might have been followed by extensive rearrangements of the target region. Thus, distinct macrosyntenic links to various legume chromosomes, observed at distal parts of linkage group NLL-03, are considered remnants of ancient chromosome shuffling. The hypothesis of the formation of *LangCHIL*1 by duplication of ancestral *LangCHIL*2 is supported by the results of our sequence similarity analysis, as the total score of Glyma06g14820 sequence alignment to *LangCHIL*2 was 6% higher than that to *LangCHIL*1.

As the second copy of *L. angustifolius* FAPa2 was present only in the transcriptome assembly, we were not able to perform microsyntenic insight. However, such an analysis was done for two *L. angustifolius CHI2* copies, derived from scaffolds KB438712.1 and KB432257.1. Sequence homology links of both copies matched the same regions in all analyzed legume species. Such an output converges with the hypothesis of gene duplications in a *Lupinus* ancient lineage. Furthermore, duplicated sequences were identified for *L. albus* CHI2 as well as for *L. albus* and *L. luteus* CHIL genes. Moreover, the presence of the closely related duplicated *CHI2*, *CHIL*, and *FAPa2* homologs in the sister branches of phylogenetic tree, supported with great posterior probability, is consistent with the most recent comprehensive legume phylogeny reconstructions. In these studies strong phylogenetic evidences were provided not only for the whole genome duplication occurrence in a common ancestor of papilionoid species, but also for the additional independent WGD events within the Papilionoideae clades, including *Glycine* and *Lupinus* lineages (Schmutz et al., 2010; Cannon et al., 2015). It was implied that in the *Lupinus* genus duplication event occurred after the divergence of *Lupinus* and *Glycine* (but before the divergence of New World and Old World lupin clades) (Cannon et al., 2015).

The assumption that *LangCHIL*1 appeared by duplication has implications for the functional specialization of that recently arisen gene copy. When multiple copies of the same gene appear in the genome, there are different evolutionary patterns possible. Homologous copies of a particular gene could acquire different functions after gene duplication, in the processes of pseudogenization, subfunctionalization, or neo-functionalization, which might result in evolutionary divergence (Blanc and Wolfe, 2004; Moore and Purugganan, 2005; Cusack and Wolfe, 2007). Our expression survey proved that both copies are actively transcribed in different organs during plant development, which negates the possibility of pseudogenization of the second copy. Substrate specificity of CHIL is unknown, however, it is well recognized that sister subfamily protein, CHI, is a branch-point enzyme, participating in several branching pathways. Those pathways include the biosynthesis of flavone, flavonol, anthocyanin, and isoflavonoid. In general, if several gene copies of a branch-point enzyme exist in the genome, they exhibit functional differentiation. Such a phenomenon was observed for two central, isoflavonoid biosynthetic pathway enzymes in soybean: 4-coumarate:CoA ligase and chalcone isomerase (Lindermayr et al., 2002; Ralston et al., 2005). Functional divergence in the CHI enzyme family was demonstrated by differences in gene regulation, expression level, kinetics, and substrate specificity. To address whether *LangCHIL* genes underwent subfunctionalization or neo-functionalization, further studies on the mysterious *CHIL* regulatory network and enzyme kinetics involving various substrates are required.

Comparative mapping of reference CHI-fold protein sequences to *L. angustoflius* genome and transcriptome assemblies enabled us to identify the representatives of all chalcone isomerase subfamilies but one, *CHI1*. The absence of any *CHI1* sequence in the collection of identified *L. angustifolius* CHI-fold sequences might reflect the loss of ancestral copy. The fact that transcriptome data from *L. albus* and *L. luteus* do not contain any CHI1- related sequence supports the hypothesis of the ancestral loss of CHI1 in the genus *Lupinus*. However, such a finding could result both from incompleteness of *L. angustifolius* genome sequence (~50% coverage) (Yang et al., 2013) and the lack of detectable expression of this gene during sampling plant material for transcriptome sequencing. Identification of gene copy number using transcriptome data is not as effective as using genome sequences. Not only are some copies not expressed, the assemblers have a tendency to illegitimately combine two similar homologs into one copy. This is much less common in genome sequences where introns help to distinguish homologs. The appearance of the recently announced upgraded reference narrow-leafed lupin genome sequence assembly (Kamphuis et al., 2015) may help to answer the question about the presence of *CHI1* gene sequence in the narrow-leafed lupin genome.

## Conclusions

1. Both chalcone isomerase-like genes present in the narrow-leafed lupin genome, *LangCHIL*1 and *LangCHIL*2, are transcriptionally active and reveal similar expression patterns.
2. Two copies of *CHI2*, *CHIL*, and *FAPa2* genes, currently existing in the *L. angustifolius* genome, are remnants of whole genome duplication which is assumed to have occurred after the divergence of *Lupinus*, *Arachis*, and a lineage leading to *Glycine*, but before the appearance of the New World and Old World lupin clades.
3. *LangCHIL*2 is assumed to be an ancestor gene, whereas *LangCHIL*1 probably appeared as a result of duplication.

### Conflict of interest statement

The authors declare that the research was conducted in the absence of any commercial or financial relationships that could be construed as a potential conflict of interest.
