# Supplementary material for: Structure, expression profile and phylogenetic inference of chalcone isomerase-like genes from the narrow-leafed lupin (Lupinus angustifolius L.) genome
Source: Front Plant Sci. 2015 Apr 21;6:268. doi: 10.3389/fpls.2015.00268 (PMC4404975; doi:10.3389/fpls.2015.00268)
Supplement: Supplementary file 1 [file Table1.PDF]

Supplementary Material 1

Structure, expression profile and phylogenetic inference of chalcone isomerase-like genes from the narrow-leafed lupin (*Lupinus angustifolius* L.) genome

Łucja Przysiecka [1,2], Michał Książkiewicz[1*], Bogdan Wolko[1], Barbara Naganowska[1]

[1] Department of Genomics, Institute of Plant Genetics of the Polish Academy of Sciences, Poznań, Poland
[2] NanoBioMedical Centre, Adam Mickiewicz University, Poznań, Poland

*** Correspondence:** Dr. Michał Książkiewicz, Department of Genomics, Institute of Plant Genetics of the Polish Academy of Sciences, Strzeszyńska 34, Poznań, 60-479, Poland.
mksi@igr.poznan.pl

1. Supplementary Tables

Supplementary Table 1. List of primers used for chalcone isomerase-like gene sequencing and gene expression assays... 2

Supplementary Table 2. List of reference and *Lupinus* spp. CHI-fold sequences 2

Supplementary Table 3. List of sequences identified in the *Lupinus angustifolius* genome and transcriptome by BLAST analysis of 172 CHI-fold sequences........ 19

Supplementary Table 4. Settings applied to construct MAFFT alignment. 23

Supplementary Table 5. Settings applied to construct MUSCLE re-alignment........ 24

Supplementary Table 6. Settings applied to perform MrBayes inference of phylogeny........ 24

Supplementary Table 7. Results of *L. albus* CHIL sequence (CA410672) alignment to NCBI Reference RNA database. 24

Supplementary Table 8. Segregation data of molecular markers tagging *L. angustifolius* CHIL genes........ 26

Supplementary Table 9. Bacterial artificial chromosome-fluorescence in situ hybridization (BAC-FISH) on *L. angustifolius* metaphase chromosomes........ 26

Supplementary Table 10. List of anchored scaffolds and contigs with alignment data. 27

Supplementary Table 11. List of repeats annotated in scaffolds with sequence coordinates. 27

Supplementary Table 12. Summary of the scaffold repeat annotation. 29

Supplementary Table 13. List of *Lupinus luteus* and *L. albus* transcriptome sequences aligned to scaffolds with alignment data. 29

Supplementary Table 14. List of transcripts identified in the annotated soybean genome by BLAST analysis of *L. angustifolius CHIL* sequences........ 46

Supplementary Table 15. Conserved sequence blocks shared between *G. max* and *M. truncatula*, *L. japonicus*, *C. arietinum*, *P. vulgaris*, and *C. cajan* genomes in regions having syntenic links to the *L. angustifolius* contigs........ 46

Supplementary Tables

Supplementary Table 1. List of primers used for chalcone isomerase-like gene sequencing and gene expression assays.

**Primers used for chalcone isomerase-like gene sequencing**			
**Primer name**	**Sequence**		
5L11_CHIL_F2	5' TTTTTCCATCGGATTTCTGC 3'		
5L11_CHIL_R2	5' GTGGCCAATGCAGTTGCAAA 3'		
5L11_F4	5' CATTGGTTTTGTTTTCACGTC 3'		
5L11_R4	5' TGATTGAGAAGATGGAGCTCTT 3'		
5L11_R5	5' TGGTTTCTCAACCCTTCAATG 3'		
5L11_F5	5' GCTCCTCCGTGAGACCTACA 3'		
28O01_CHIL_F3	5' TCTTGAGTGTTCTTTGAATGG 3'		
28O01_CHIL_R3	5' CCGCAATGTAAAACCCCTTT 3'		
28O01_F5	5' TCTCCCTTGGGCATTTATGA 3'		
28O01_R5	5' CAGTGGAGGAAAAATGGAGAA 3'		
28O01_R6	5' CGAGCACCACCCTTCATTAT 3'		
28O01_F6	5' CTCCTCTTATTGGGGCTGC 3'		
28O01_R7	5' ATCCAAGCTTCGCAAAAGAA 3'		
28O01_F7	5' TCCACATTTTGATCCACTTAGC 3'		
**Primers used for gene expression assay**			
**Primer name**	**Sequence**	**Annealing temp. (°C)**	**Product lenght (pz)**
CHIL_1_2_F	5' ACTTGCAGCAGTGGAAAGGT 3'	60	DNA: 720
CHIL_1_3_R	3' TCATCAGCTGCCAATCTGTC 5'		cDNA: 220
CHIL_2_2_F	5' TGGAGTTTACTTGGACCCTGA 3'	60	DNA: 859
CHIL_2_3_R	3' AACCTGTCCCTCACAGCACT 5'		cDNA: 260
18S_RNA_F	5' AAACGGCTACCACATCCAAG 3'	60	460
18S_RNA_R	3' TCATTACTCCGATCCCGAAG 5'		
TUB_F	5' TCGGTTCCAAATTCTGGGAAGTT 3'	54	387
TUB_R *	3' GAACCAGTGCCTCCTCCAAG 5'		
* Primers selected according to: Foley R, Gao L-L, Spriggs A, Soo L, Goggin D, Smith P, Atkins C, Singh K. 2011. Identification and characterisation of seed storage protein transcripts from *Lupinus angustifolius*. BMC Plant Biology 11(1): 59.			

Supplementary Table 2. List of reference and *Lupinus* spp. CHI-fold sequences

(Ngaki et al., 2012; Chu et al., 2014; Liu et al., 2015; Dastmalchi and Dhaubhadel, 2015)

>Phaseolus.vulgaris.Phvul.003G216600.1
MMATPPSITSVTVEFLQFPAVVTPPSSTKSYFLGGAGVRGLNIEGEFVKFTGIGVYLEEKAVAWLCAKWKGKNAAELLESL EFYRDIIKGPFEKYIRGSKLRTLDGPEYVRKVSENCVIFMKSVGSYGEAEEKAIEEFRYAFKDQNFPPGSTVFYRQSPTGTLG LSFSKDDTIPENEYVVIENKALSEAVLETMIGEIPVSPALKESLATRFYEFLKEDNSNTE
>Phaseolus.vulgaris.Phvul.007G008600.1
MATAPTITDVQVEFLHFPAVVTSPATAKTYFLGGAGERGLTIEGKFIKFTAIGVYLEDKAVASLATKWKGKPSEELINTLDF

YRDIISGPFEKLIRGSKILQLSGTEYSRKVMENCVAHLKSVGTYGDAEAKGIEEFAEAFKKVNFPPGASVFYRQSPDGILGLS<br>FSEDATIPGEEAVVIENKAVSAAVLETMIGEHAVSPDLKRSLASRLPAVLNGGIIV
>Phaseolus.vulgaris.Phvul.007G008500.1
MSLPSVTAVDVDTVTFPPAVNPPSSSVAFFLAGAGVRGLQIQDKFVKFTAIGIYLQPDAVPLLSVKWNAKSAPELTDSVEFF<br>RDIVTGPFEKFMQVTMILPLTGQQYSEKVSENCVAIWKSLGIYTDAEAEAIHKFVSVFKDQTFPPGSSILFTVLPKGSLLISFS<br>KDGSIPKEVIAVIENKLLSEAVLESMIGKHGVSPAAKQSLASRLSELFKQG
>Phaseolus.vulgaris.Phvul.002G276500.1
MLDGATFHFPFLYLPFINPPLFIFNFSFHFTSTHQIHSLLPMSLPSVTALDVDNVTFPPTVNPPSSATAFFLAGAGVRGLQIQD<br>NFVKFTAIGVYLQPNAVPLLSVKWNGKSAPELTDSVEFFRDIITGPFEKFMQVTMILPLTGQQYSEKVSENCVAIWKSLGIY<br>TEAEAEAIDKFVSIFKDETFPPGSSILFTVLPKGSLLISFSKDGAIPEEASTVIENKLLSEAVLESMIGKHGVSPAAKQSLASRLS<br>ELFKEGGVPESHN
>Phaseolus.vulgaris.Phvul.009G143100.1
MATEQVLVDEIPYPSKITTTKPLSLLGHGITDMEIHFIHVKFYSIGVYFEPEVVSHLQQFKGKPAKELEQNDEFFDALISAPVE<br>KFIRLVVIKEIKGAQYGVQIESAVRDRLAAEDKYEEEEEEALEKVIEFFQSKYFKKHSVITYHFPADSATAEIVVSLEGKEDS<br>KFVLENANVVEAIKKWYIGGSTAVSTTTIQSLASTFSEELSK
>Phaseolus.vulgaris.Phvul.005G064600.1
MAGAVAASTALGFSPLTHPTRILVHKGVSNSTLPLSSHGQSFSLLSSTPMHFSSHKSSRRQPLFLAQVASSSAANAEYVEEPE<br>TNVKLQTCLNFPGCSNSLTLFGTGYRENVFAIVSVKVYTAGLYLDQHITRELNAWKGQSKDAIQGNSSLFQTIFQSSFGKSL<br>QIILARNIHGKTFWEALSDAISPRIPASTTADEIALSIFRSVFLDRPLKKGTFIILTWLKPSKLLVSVSSNGLPSTVDAAIESENV<br>SCALFNVFLGDSPVSPSLKASVAEGLSKVLK
>Phaseolus.vulgaris.Phvul.005G064500.1
MTKNVIVVLTVAASTALGFSPLTHPTRILVHKGVSNSTLPLSSHGQSFSLLSSTPMHFSSHKSSRNQPLFLAQVASSSDANAE<br>YVEEPETNVKFQTCLNFPGCSNSLTLLGTGYREKIFAIVSVKVYAAGLYLDQHITRELNAWKGQSKDAIQGNSSLFQTIFQS<br>SFEKSLQIILARNIDGKTFWEALSDAISPRIPSSTTVDEIALSIFRSVFLDRPLKKGTFIILTWLNPSKLLVSVSSNGLPSTIDAAI<br>ESENVSCALFNVFLGDSPVSPSLKASVAEGLSKVLK
>Phaseolus.vulgaris.Phvul.001G152000.1
MASMRFPFSFPQPQPPQFPRPFTAFAAVAAVAGASAAVVAVSSSDRPFLRNALNSFFSSGHSLPLWGSLSLADSGVSVLESK<br>TGTSFPSVLASSHKLCGIGLRKKSVLGLKNIDVYAFGVYADDDDIKTHLSEKYGKFSASELQGNKEFIGDLMDNDISMTVR<br>LQIVYGRLSIRSVRSAFEESVGSRLQKFGGSDNKELLQRFTSQFRDEIKIPRGSVIHLSRDKGHVLRTSIDGQEVGSIKSKLLC<br>KSILDLYFGEEAFDKQAKEEIEHNVASYL
>Phaseolus.vulgaris.Phvul.001G037700.1
MNNDFVWLPSLYSDPEVFTYLEPFLLRNSLSSRFVHSLANNPGSFAIGEAFGHVSRFAGAFLVWLSRASTFNVARSLRGSPP<br>PPPRFGGAQVKAVATNVRLFGFPFRSKRKSFASVKLGRVSSLAMKMIWSEAKRLRSLPLLSLAAAFVPPFHNLSSNVLACP<br>LHSPDMQVYGTIDQVPKEVECQGCPFLSYLELNEAKPAVEPKTGIEFPLVLDNIFVGEKDFGFNSEVLVGTGSRTMTIVKIK<br>SLKVYAFGVYIHPYSLCEKLGPKYASISADELNNHHDLYRDLLREDINMTVRLVVNCRGMKINSVRDAFEKSLRARLVKT<br>NPSTDFHCLETFGSYFEENISIPLGTVIEFKQTIDGRLITKISGNQIGSVHSKDLCRAFFDMYIGDVPVSEETKKEIGTNIANIIR<br>RC
>Medicago.truncatula.Medtr1g115850.1
MAKAAFMITGVKVEFIEFPAVVTPPSSTKSYFLGGAGVRGLDVDGEFVKFTGLGVYLEEKAVASLTLKWKGKTPSQLFESL<br>DFYRDIIKGPFEKFIRCTKVRTLEGSEYVRKVSENCIAHMKFEGTYGDAEEKAIQELREAFKVQFFPPGAGVFFRQSPNGAL<br>GLRFSKDETIPEHEYVVINNKPLSEAVLETMIGEIPVSPALKESLATRFYEFMKIDNFNI
>Medicago.truncatula.Medtr1g115840.1
MATAAPTITGVKVENIEFPAVVTPPASPKSYFLGGAGVRGLDIDGEFVKFTGIGIYLEEKAVASLTPKWKGKTPSQLFESLEF<br>YRDIIKGPFEKFIRSTKVRTLEGSEYVRKVSENCIAHMKSEGTYGDAEEKAIQEFREAFKDQFFPPGTAAFYRQSPNGALGL<br>RFSKDETIPEHEYAVINNKPLSEAVLETMIGEIPVSPALKESLATRFYEFLKIDNFNIRN
>Medicago.truncatula.Medtr1g115830.1
MCQKDFLKNLVKNELVKELDLNLPYSHQCSQDKKLNVEFLEFPATVTPPDSTKTYFLGGAGHGNAVTALASKWKGRTAA<br>ELLESLDFYRDIIKGPFEKLIRGGKLKTLDGREYVRKVSENCIDQNFPTGASVFYKQSPTGTLGLRFSKEETMIGEISVSPALK<br>ESLATRFYDLMKINNFNMGN
>Medicago.truncatula.Medtr1g115820.1
MAASITAITVENLEYPAVVTSPVTGKSYFLGGAGERGLTIEGNFIKFTAIGVYLEDIAVASLAAKWKGKTSQELLDTLDFYR<br>DIISGPFEKLIRGSKIRELSGPEYSRKVMENCVAHLKSVGTYGDAEAEAMQKFAEAFKPINFPPGASVFYRQSPDGILGLSFS<br>PDTSIPEKEAALIENKAVSSAVLETMIGEHAVSPDLKRCLAARLPALLNEGAFKIGN
>Medicago.truncatula.Medtr1g115890.1

MATPSVTSLAIESIVFPPTMKAPGSTNNFFLGGAGVRGIQIQDKFVKFTAIGVYLQDIAIPYLAAKWKGKPPHKLTESVPFFM<br>DIVTGPFEKFMRVTMIRPLTGQEYSNKVSENCVAIWKSLGIYTNEEAKAIKKFVSVFKDETFPPGSSILFTVSPKGLGSLTISF<br>SKDGSIPEVETAVIENKLLSQAVLESMIGAHGVSPAAKQSLASRLSKLFKEGGNANN
>Medicago.truncatula.Medtr1g115870.1
MALPSVTALEIENYAFPPTVKPPGSTNNFFLGGAGERGIQIQDKFVKFTAIGVYLQDIAVPYLAEKWKARSAHELTDTVPFF<br>RDIVTGPFEKFMRVTMILPLTGHQYSEKVSENCVAIWKSLGIYTDEEAKAIDKFVSVFKDETFPPGSSILFTVSPKGLGSLTIS<br>FSKDGSIPEVETAVIENKLLSQAVLESMIGAHGVSPAAKQSLASRLSKLFKEGGNANN
>Medicago.truncatula.Medtr1g115880.1
MVYIEVQKVQGNALEKKNGSIPEVETAVIENKLLSQAVLESMIGAHGVSPAAKQSLASRLSKLFKEEDEDSVLGIGLGSGS<br>VLLLKISG
>Medicago.truncatula.Medtr1g015700.1
MKNDWLSWMDTDPNLFPFEPFLFFLDNSKTFYESSAIEEAFGRVSKFAGALLFWFSGGGSGGGSLRFGGGGGFGNVNLKV<br>KPVITNNVAPRFGFGFGSKRKTCERVSLGKISSFVVRLFWREAKRIQSFPVLSLAAALVPPIQNLSSNLLSGPMQDPDVQMH<br>GGMDQVPKDVERRGCPRLSISELSLANSTVEPKTGIEFPVVLDNLSAGDRNSSLGSEVLVGTGSKNMTIVKIKTLKVYAFGF<br>YVHPYSLCEKLGPKYASISADELNDRNDFYQDLLREDINMTVRLVVNCKGMKINSVRDAFEKSLRARLVKTNPSADFDCL<br>WTFGSYFTENIPIPLGTIIEFKRTVDGRLITEIGGNHVGSVHSKDLCQAFFGMYIGDVPVCEQTKKEIGTNIVNIIRNC
>Arachis.hypogaea.JN660794
MVLPSSLSAVQVDNVTFPATAKPPGSDKTFFLGGAGVRGLQIDDKFVKFTAIAVYLQDNAVPSLAVKWNGKTPSELTESV<br>DFFRDIVTGPFEKFMQVTMILPLTGQQYSQKVSENCVAIWKHLGIYTDQEANAIDKFLSVFKDQNFPPDSSILFTLLPNGSLV<br>IGFSKDGSIPEAGTAVIENKLLSEAVLESMIGKHGVSPAAKQSLATRLYELFKQSGHDDTADKKLHDFDTDNGLHHTKSGN<br>SVAEDAAEKLS
>Arachis.hypogaea.KP202691
MVKAASLAGVNVEFLEFPPVVTPPGTTKSYFLAGAGVRGLPINGVFVTFTGLGLYLEDKAVPYLASRWKAKTPAQLLDSL<br>HFYRDIIQGPFEKLIRGSKLKTLDGPEYVRKVSENCVAYMKSVGTFGDAEEKAIHEFRQAFKDQNFPPGSTVFYEQFPNGTL<br>GLKFSKDDTIPEHRNAFIENKALSEAVLETMIGEIPVSPAFKESLATRLSQILNEANPSIEF
>Acidovorax.avenae.ZP_06210709
MTLRQRSLAWALAGLIACGAVPAGAQTATTVAGVAFEPRVTLADVPLALNGAGVRYKAVFKVYAAGLYLERHAGTLQD<br>VVALPGPKRLSITMLRDIDSTELGKLFARGIEDNLDKAAFSRLAPGVLRMGEIFAAHRRLAAGDRFTVDWLPGTGTVITVK<br>GVPQGEPFREPEFFDALMGIWLGPQPADWKLKDALLGKAG
>Acidovorax.ebreus.YP_002553831
MPWSRISSLKRWAFAGGAALALAVASAGPTTGQDGGAAPAWVRQSLVGATLAGEGQMRFLGLRIYDARLWVTPGFDAS<br>RFGAHPLALELTYHRGFTGAAIARRSVEEMQRQASVPSEQAERWQQRLAAVIPDVQPGERLTGLYQPGQGMRLWRGSQE<br>LAAIDDAELARLFFGIWLSPQTSEPGLRSALLARIPGGAAP
>Aeromonas.salmonicida.YP_001142961
MNWKIPRIGTWGGISLLIAALGAMMALSAQASAAPWQQLRTVGQGEMNWLWFKLYDATFYSQSGRYQPGHYPQALTLT<br>YARDIERDDLLTATAEEWQRLGLGSETQRQQWLGQLAVFWPDVAAGDTLTFYVDEAGVGHFWWQYKPLGTLPDPQFAA<br>AFLAIWLADNSRDPALTRRLRGQL
>Aim18_S.cerevisiae.NP_012068
MDRGRCANMLKSLQRTLAKCQKSPSTNHWQCFKRNFTSIRATKYPGRSNSTFHYWPWFAASTLLATSLYYRDRPVQNDD<br>KTDAFPSHTESIQVDSSVSDFPLTITALNFPVSTTFKLLGYGQRHVTFLRFKVYALGLYLAENDENLVSDTLNETYLHKYFL<br>DVDDSKTPKENLARLLKRDDSKSVMMIDDLLDSGMRMLAKITPVRNTDFKHLKEGLVKTISKHPDVANNKDTLAKGLSE<br>LNDAFSRKGSVRKNDDLIIELLANGALQFSYHDSKNNEFEVMGVVNNQLVGKFLFSQYLCGEKSPSPQAKKTAIDKLITLL
>Aim46_S.cerevisiae.NP_012069
MRLISKVLVKTNCLEVGMRRAPQWYSHYSTTAGNARVNKKGSKVVPVLTGLALASIFAKKWYDDSQIKKADATSVAVD<br>ASISAFPKKMGPPQWPFSTQYELIGKGVRCVSSITFKAYGLGIYVAAEDKHLVSEVLDSKFLSQAFIDTAAPPSPENSHQDNL<br>RAALNDPAKAPILINNLLDSGIRLMSKNTPIKAGSFKLLMDGTKKSVLKNPDSQSQDKDRLEAGFQELHDCFRSVKGLVAR<br>DDDFFIELNKDCSMNLSYYARKKDEFVILGTVKEPLIGKLLFAHYLAAVDPPSPEARKEVIDALVSLS
>Anaeromyxobacter.dehalogenans.YP_002491670
MRKAAAVAALLLVLPLGAAARKVAGVELPDSVTVEGKELRLNGAGIRKKLWIEVYVGALYLATPSSDANAILAADEPRR<br>VRMVFRRDVDQKSIMGAFRDGFEANSGAEAAALVPQLDRIAPAIGDVKKGGEITVTYLPGTGAVVTGPKGTATVEGKAFA<br>DALFRNWLGRKPADDDLKRRMLGK
>Asticcacaulis.excentricus.ZP_04771225
MTAFAVRAEVPAGVPDGKLVGRGRYSVLGLSIFDASLYAPNGRYDPQKPFALRLDYLKSFNARYIVDHSIAEIRKQGFSDK

VKLEEWRQQMSAIFPDIRSGAYIAGVRNADGYAQFIHNGRAIGAIRDTQFTRLFFDIWLGSRTSSKALRAKLLGDA
>Bordetella.avium.YP_787063
VFYRPSLIQRCLCAATLALSFQASAQSGIVLDGIAIPKQVIQGEQSLPLIGAGLCSEFIFNIYLAALYARPISHEAQALIAGPGP<br>RRLHLQFLRDISASTLEQAVRQGLEANHSAADVKSLKPAIDTLSALLHRIGQLSNGDIIDLAMEPGAVTIFYNGQSQGRIDDT<br>NLAAALLRIWLGDNPAQASLKEALLGQN
>Burkholderia.vietnamiensis.YP_001115087
MTRRFAALAAVAAWAAFAMPGAADANCVDDVASARLIGSGRFCVFAICLYDAQLWARRAPGAFDTPFALSLAYRHDVT<br>GERLVATGMDEIERLAAAPLPAATRDAWRRDIARAFTDVSRGDVLCGVYLPERGARFYTNGRLTAEVADPAFARAFFEIW<br>LDPRTRATSLRRQLLGDDAR
>Burkholderia.xenovorans.YP_557607
MRAFAALVFALWASAACAGWRGDVQAAKLVGEGDFSVLGFTLYRAQMWSERVPVDYDERFALHIVYAHGVKRERLAD<br>TGIGEIRRLAAAPIPADTLARWRANMMQAFVDVGPGDQLSAVYLPDKGVRFYAGERMTGEVDDLAFARAFFGIWLDPST<br>RAPVLRRQLLGAGQ
>CHI_Aquilegia.formosa.DR924655.1
MTSSDLSSSSSSLARLNMELKVEVYTFCPTLSTAPGSANSFFLGGAGVRGLQIQDKFIKFTAIGVYLEEQKAISSLAVKWNG<br>KSAEELTDSIEFFRDIVTGPFEKFIMVTMILPLTGQQYSDKVVENCVAAWKAMGIYTEAESKAVEEFIQVFKDETFPPGSSIL<br>FTQSSPALTIGFSKDGSIPKEGKATIANKPLSEAVLESIIGKHGVSPEAKHSLAVRISEMMKNQGDTVEEGMVQKMENGEPV<br>KIYPLPN
>CHI_Arabidopsis.thaliana.CAB94981
MSSSNACASPSPFPAVTKLHVDSVTFVPSVKSPASSNPLFLGGAGVRGLDIQGKFVIFTVIGVYLEGNAVPSLSVKWKGKTT<br>EELTESIPFFREIVTGAFEKFIKVTMKLPLTGQQYSEKVTENCVAIWKQLGLYTDCEAKAVEKFLEIFKEETFPPGSSILFALS<br>PTGSLTVAFSKDDSIPETGIAVIENKLLAEAVLESIIGKNGVSPGTRLSVAERLSQLMMKNKDEKEVSDHSVEEKLAKENRE
>CHI_Ipomoea.batatas.BAA90334
SAPPCVAEVKVESYVFPATAKPPGTAKTLILGGAGARGLNIDGKFVKFTAIGVYLEADAVPSLAVKWNGKSAEELTDSVQF<br>FRDIVTGPFEKLTRITMILPLSGKQYSEKVSENCVAFWKAAGIYGDAESKAIEKFNDVFSDQMFPPGASIFFTQSPLGWLTISF<br>SKEGSMPEIASAVIENKPLSEAVLESIIGSKGVSPEAKQSLAVRLSELFKNGVSGGDAITGKVGCENDVIPQTVVSK
>CHI_Oryza.sativa.NP_001051714
MAAVSEVEVDGVVFPPVARPPGSGHAHFLAGAGVRGVEIAGNFIKFTAIGVYLEEGAAVPALAKKWAGKSADELAADAA<br>FFRDVVTGDFEKFTRVTMILPLTGEQYSDKVTENCVAAWKAAGVYTDAEGAAADKFKEAFKPHSFPPGASILFTHSPPGVL<br>TVAFSKDSSVPEGAVAAAAIENRALCEAVLDSIIGEHGVSPAAKRSIAARVSQLLKAESTGDVAAAEPAPVSA
>CHI_Selaginella.moellendorffii.XP_002980623
MAPELLKQGLVIEGTQFPPGLTSPVSQKSLFLAGAGVRSIQINPQVTITVTVLGFYFEDGILKHLSGKWSGKSGSELEKEDDF<br>FTDIINAPSEKIFSVTMLKPLPGTEFSKKVMENTKQVLAESNSLGEDEEKAIEEFSKLFEDQALKPGMGPFYVSSSSGLGVGF<br>TDPDEPSNLKISSSIGNVKFANALLSTMIGKNPVSPASKACIAQRLSALL
>CHI_Vitis.vinifera.P51117
MSQVPSVTAVQVENVLFPPSVKPPGSTNDLFLGGAGVRGLEIQGKFVKFTAIGVYLENSAVPTLAVKWKGKTVEELADSV<br>DFFRDVVTGPFEKFTKVTTILPLTGRQYSDKVSENCVAFWKSVGIYTDAEAKAIEKFNEVLKDETFPPGNSILFTHSPLGALT<br>MSFSKDGSLPEVGNAVIENKLLTEAVLESIIGKHGVSPEAKKSLAARLSELFCKEAGDEKIEAEKVAPVAC
>CHI_Zea.mays.Q08704
MACRRWWSTAVVFPPVARPPGSAGSHFLGGAGVRGVEIGGNFIKFTAIGVYLEDAAVPALAKKWGGKTADELASDAAFF<br>RDVVTGDFEKFTRVTMILPLTGEQYAEKVTENCVAFWKAAGLYTDAEGVAVEKFREVFKPETFAPGRSILFTHSPAGVLTV<br>AFSKDSSVPAAGGVAIENKRLCEAVLESIIGERGVSPAAKLSLAARVSELLAKETAAAADAPQAEPVSITA
>CHI1_Lotus.japonicus.Q8H0G2
MAPAKGSSLTPIQVENLQFPASVTSPATAKSYFLGGAGERGLTIEGKFIKFTGIGVYLEDTAVDSLATKWKGKSSQELQDSL<br>DFFRDIISSPSEKLIRGSKLRPLSGVEYSRKVMENCVAHMKSAGTYGEAEATAIEKFAEAFRKVDFPPGSSVFYRQSTDGKL<br>GLSFSLDDTIPEEEAVVIENKALSEAVLETMIGEHAVSPDLKRCLAERLPIVMNQGLLLTGN
>CHI1a_Glycine.max.Q93XE6
MATISAVQVEFLEFPAVVTSPASGKTYFLGGAGERGLTIEGKFIKFTGIGVYLEDKAVPSLAAKWKGKTSEELVHTLHFYR<br>DIISGPFEKLIRGSKILPLAGAEYSKKVMENCVAHMKSVGTYGDAEAAAIEKFAEAFKNVNFAPGASVFYRQSPDGILGLSF<br>SEDATIPEKEAAVIENKAVSAAVLETMIGEHAVSPDLKRSLASRLPAVLSHGIIV
>CHI1b1_Glycine.max.Q53B75
MATPASITNVTVEFLQFPALVTPPGSTKSYFLGGAGVRGLNIQEEFVKFTGIGVYLEDKAVSSLAAKWKGKSAAELLDSLD<br>FYRDIIKGPFEKLIRGSKLRTLDGREYVRKVSENCVAHMQSVGTYSDEEEKAIEEFRNAFKDQNFPPGSTVFYKQSPTGTLG<br>LSFSKDETIPEHEHAVIDNKPLSEAVLETMIGEIPVSPALKESLATRFHQFFKELEANPNIEN

>CHI1b2_Glycine.max.Q53B70
MATPASITNVTVEFLQFPALVTPPASTKSYFLGGAGVRGLNIQEEFVKFTGIGVYLEDKAVSSLGAKWKGKSAAELLDSLD<br>FYRDIIKGPFEKLIRGSKLRTLDGREYVRKVSENCVAHMESVGTYSEAEEKAIEEFRNAFKDQNFPPGSTVFYKQSPTGTLG<br>LSFSKDETIPEHEHAVIDNKPLSEAVLETMIGEIPVSPALKESLATRFHQFFKELEANPNIEN
>CHI2_Glycine.max.ACU13680
MAFPSVTSVTVENVTFPPTVKPPCSPNTFFLAGAGVRGLQIHHAFVKFTAICIYLQYDALSFLSVKWKTKSTHQLTESDQFF<br>SDIVTGPFEKFMQVTMIKPLTGQQYSEKVAENCVAIWRSLGIYTDSEAEAIDKFLSVFKDLTFPPGSSILFTVSPNGSLTISFS<br>GDETIPEVTSAVIENKLLSEAVLESMIGKNGVSPAAKQSLASRLSHLFKEPGVCDPQSHK
>CHI2_Lotus.japonicus.Q8H0G1
MALPSVTALQVENVAFPPTLIKPPASANTLFLGGAGERGLHIQDKFVKFTAIGIYLQDTAVPSLAVKWKGKPVDELTESVQF<br>FRDIVTGPFEKFMQVTMILPLTGQQYSEKVSENCVAIWKHLGIYTDEEGKAIDKFVSVFKDQTFPPGSSILFTVLPKGSLAISF<br>SKDGSIPEVESAVIDNKLLSEAVLESMIGAHGVSPAAKQSLASRLSELFKHHAEV
>CHI3_Glycine.max.ABA86742.1
MAFPSVTSVTVENVTFPPTVKPPCSPNTFFLAGAGVRGLQIHHAFVKFTAICIVYLQYDALSFLSVKWKTKSTHQLTESDQF<br>FSDIVTGPFEKFMQVTMIKPLTGQQYSEKVAENCVAIWRSLGIYTDSEAEAIDKFLSVFKDLTFPPGSSILFTVSPNGSLTISFS<br>GDETIPEVTSAVIENKLLSEAVLESMIGKNGVSPAAKQSLASRLSHLFKEPGVCDPQSHK
>CHI4A_Glycine.max.AAT94362
MATEEVLVDEITYPTKITTTKPLSLLGHGITDMEIHFIHVKFYSIGVYLEPEVVGHLDQFKGKSAKELEDNEEFFNALISAPVE<br>KFIRLVVIKEIKGAQYGVQIETAVRDRLAAEDKYEEEEEEALEKVIEFFQSKYFKKLSVITYHFPANSATAEIVVSLEGKEDS<br>KYVIENANVVEAIKKWYLGGSSAVSSSTIQSLASTFSQELSK
>CHI4B_Glycine.max.CX707131.1
MATEEVLVDEITYPSKITTTKPLSLLGHGITDMEIHFIHVKFYSIGIYLEPEVVGHLEQFKGISAKELEENDEFFNALISAPVEK<br>FIRLVVIKEIKGAQYGVQIETAVRDRLAAEDKYEEEEEEALEKVIEFFQSKYFKKHSVITYHFPTNSATAEIVVSLEGKEDSK<br>YVIENANVVEAVKKWYLGGSSAVSSSTIRSLASTFSQELSK
>CHIL_Aquilegia.formosa.DT728112.1
MGSEMVMVDDIPFPPQITTTKPLSLLGHGITDIEIHFLQIKFTAIGVYLEPEVVEHLKQWKGKTASELAEDDDFFDALISAAV<br>EKFLRIVVIKEIKGSQYGVQLESAVRDRLAADDKYEEEEEEVLEKVVEFFQSRYLKKHSIITFSFSPKSDSLEISLSAEGKEES<br>KIKVENANVVEMIQKWYLGGTRGVSQTTISSLANNLGTLLS
>CHIL_Arabidopsis.thaliana.NP_568154
MGTEMVMVHEVPFPPQIITSKPLSLLGQGITDIEIHFLQVKFTAIGVYLDPSDVKTHLDNWKGKTGKELAGDDDFFDALAS<br>AEMEKVIRVVVIKEIKGAQYGVQLENTVRDRLAEEDKYEEEEETELEKVVGFFQSKYFKANSVITYHFSAKDGICEIGFETE<br>GKEEEKLKVENANVVGMMQRWYLSGSRGVSPSTIVSIADSISAVLT
>CHIL_Ipomoea.nil.BJ578539.1
MGTEMVMVDEIPFPPQVNLDNKLLSLMGHGITDVEIHFLQIKYTAIGVYLDPEIVSHLQKWKGKTPVDLAQDDDFFEAIIN<br>APVDKVLRVVVIKEIKGSQYGVQLENSVRDLLAEVDKYEEEEEAALEKVVDFFQSKYFKKSSVITFSFPANTATAKIVFATE<br>GKEDSSIEVENANVGGMIKKWYLGGSRAVSPSTISSLANILPD
>CHIL_Oryza.sativa.NP_001065587
MGTEIATVEVEGIPFPQEITVSKPLSLLANGITDIEIHFLQIKYNAIGVYLEKDNVLAHLESWKGKKAEELVQDDGFFQALVS<br>APVEKLLRIVVIKEIKGSQYGVQLESSVRDRLVSVDKYEEDEEEALEKVTEFFQSKYFKPNSVITFHFPTTPGIAEISFVTEGK<br>GEAKLTVENKNVAEMIQKWYLGGESAVSPTTVKSLADQFAALLSA
>CHIL_Selaginella.moellendorffii.FE507018.1
MEMDPTFAQSIQSPSSSETLILLGHGITDMTIETIHVIFTKIGVYFAPQVKDHLQSFKCLPVSELLKDGSAFFQQLIQAPVSKLI<br>KILLVKGQLGSQYASTIETSVRDRLAYDDKYEEDEEIALANLCEFFQSKKLEPNSTIVYSWPSSSSHVEVFVHEEGSKAPSSFI<br>VNNENVSTSIIEWILGENSMTPSTVESVAKSIATEC
>CHIL_Syntrichia.ruralis.CN201507.1
MGLPVVKVEEIDYTTTITPPGASSSLDLIGYGNTGMEIETVEIRFTALGFYAAAPALKEHLSKWKGKPASELVEDDSGFHKE<br>LIQSPGEKLVRISIIKGIKGLPYGSALQSSLRDRLVNDDKFEEEEEEALEKMVEFFQGHNLPKGANILYYWPTPASVQISLSEE<br>DGKIPEEVAYTIADANVAEALLDLYFGENTITPSTLSSVAEAIAX
>CHIL_Vitis.vinifera.XP_002280158
MGTEMVMVDEIPFPPQITTAKPLCLLGYGITDIEIHFLQIKFTAIGVYLEPEIVGHLQPWKGKSGKELAENDDFFEALISAPGE<br>KFLRIVVIKEIKGSQYGVQLESAVRDRLAADDKYEEEEEEALEKVVEFFQSKYFKKDSIITFHFPATSCTAEIVFATEGKEES<br>KITVENANVVEMIKKWYLGGTRGVSPTTISALANTLATELSK
>CHIL_Zea.mays.NP_001151452

MGSETETVNVEGIPFPAEITVGNPLSLLGTGITDIEIHFLQIKYNAIGVYLHNAGGGDSTTPTLLGHLGAWKGKTAEELLADA AFWAALVAAPVEKLFRVVVIKEIKGSQYGVQLESSVRDRLAAADLYEDDEEEALEKVADFFQSKYFKPGSVVTFHFPAPA SASPGPAAEITFATEGKGDARIAVENGNVAGMIQTWYLGGDSAVSPSTVRSLANRFAALLAVAA
>CHILa_Physcomitrella.patens.XP_001773128
MGPQVVKVEDIDFATKFTPPTGSTELDLIGYGNTGMEIETVEIRFTAIGFYAEPSISEHLQKWKGTPSSNLVEDDSGFHKELI QAPVEKAVRISIIKGIKGLPYGSALQSSLRDRLVNNDLFEEEEEEALEKLAEFFQPHNLPKGTNIIYHWATPSSVKVSLSEEG KMPEDVAYTIDDAHVAEALLDLYLGENTITPSTLASVAEAIAA
>CHILb_Physcomitrella.patens.XP_001769093
MGLQVVNVEGIDFATKFAPPTSSTELDLIGHGNTGMEIETVEIRFTAMGFYAEPSISEHLQKWKGKAVSELVEDDSGFHKEL IQVPVEKAVRISIIKGIKGLPYGSALQSSLRDRLVNDDKFEEEEEEALEKLVEFFQPHNLPKGANIIYHWATPDTVKISLSEEG KIPDEVSYTIEDANVAEALLDLYLGENTITPSTLSSVAEAIAAQVA
>Colwellia.psychrerythraea.YP_268116
MFKLSFSLCSSYLSYFYLICCLSQSVVFANSISDEMELPNNLQSTIDTQAFVSIGETTFSILFWDLYKSQLLTTSGKYPINIATD KVLFTINYLADISSEDLINRTIDQWDHLGIPAEEYLTYVSDLKNMWPDIKEGDSLSLLIHQGRSVFYFNQQYIGVITPSEFGQI FLAIWLSENTSEPELRRELLGSITYD
>Cupriavidus.metallidurans.YP_582677
MKSLKSPSRTTALSRRFAATLCATLALWTLMPPASAMVIEGVRFDDATRLGGKELALNGAGLRSVFVIKGYVAGLYLPER ARNATVALATRGPKRLQLRVLREVEAESFIKALNEGLRENHSELQMRMLADRLAQLQQTMTEMGAAHRGDIINFDFTPEG GTVVALNGIPRGQAIAGEDFYQAVLRIFLGDHPVDRNLKRGLLGG
>Deferribacter.desulfuricans.YP_003497050
MKKLSIILLLLVLIAPVGYSKVVKGVNIPDTYQYFGKNLVLNGTGFRKKFFIKVYIGALYLEQKTDDANAVISAPTKVVKM HFLYKKVKASQMKDAFKESFEKIDENLLKESAVQEFLNAVSFDVVKGDEVDLIIDNDMVTVLKNGDKIGDFKSKSLADALI KIYVGDEPADSGLKEGLLGKE
>Delftia.acidovorans.YP_001561779
MTALPRWMKTAALAMALAMPLAATAQEPPRTVAGVAYPAQAGVGGQTLQLNGAGIRYKAIFKVYTAGLYLEKPAASLQ EIAALPGPKRVSVTMLREIDSAELGKLFARGIEDNMERARFSRLVPGVLRMSDIFTQHKKLLPGENFSVDWVPGQGAQVFV KGQAQGAPFQEPEFFQALLGIWLGPHPADEQLKKALLGG
>Desulfatibacillum.alkenivorans.YP_002433609
MKKVRQLSSFIFILCLICALTAPALAKDFHGWPLPDKMPADESTDLVLNGAGFRTKFLLKIYVGALYLTHKETDANKIINAD EYMGVRMHFTFRKVGHDSLVEILNTGFDKATNGDTSGIQSQIDHLFALLPQEFCKNDIVDLLYVPGRGIVTHINGKYAGEC PGLDFKQAVFAIWLGDEPVTEKLKAEMLGIETS
>Desulfococcus.oleovorans.YP_001528241
MRIMPKQRFINRLVTTWVLIFCFTTGLYGAQVNGADFEKAVYVSEKRLTLRGAGLLRYLVVIKAYAGAFYLEAGLPPDRA LENVTRQLVLHYFHAIPAEDFATATTTMIEKNVTPDQFARLAPLVDRMNALYRDVAPGDRYTATYIPESGTELALNGQAL GTVPGAAFSRAFFSIWIGENPIDKGFRNDLLKGLAP
>Desulfomicrobium.baculatum.YP_003158573
MIRKYIFTCLIILSIASPASGGQIGDVTLPDQITVADSTLTLNGLGMRTFTFFDVYAAGLYLPTPSTDAGKILATDAPRGMTM HFLRKVEAEKIADAWLEGLAANTPAADASLQERFVALGSMMETMDKGETLSCLYNPADGTTIMVRGQLKGVIPGKDFND ALLGCWIGPKPGPGEKFKAGILGQR
>FAP_Ashbya.gossypii.NP_982871
MLNKRISNLATILRFIALPRARNIIAPQEYIANSVPVAITRRAARNTFVFSVALTPFVLYSAFHNDSAESDFPELVEVYPGVRP FPAVLGPPELPLQTNYKLLGHGVRAVTFLSFKVYALGIYAAVDDLPLIPRTLSAEYLSTLDTEKVDAPAKEQLYKAMQDHE KSRAVVNDLLGKGLRLVAKITPIRNTDFTHLKDGLVKSILNHPAARHEGETLANGLEELRAAFTRRGSVPKDDDLVLELQA TGALQLYHRDAKTGQTTTLGKVTEPLIGRYLFSQYLSGKAPLSKDTKDSVTRKIISMV
>FAP_Aspergillus.nidulans.XP_661820
MAFKPPLSVSKSILFRNCGQCIRNRYLRPTATSRYLSTSSPLRNNPLRARANANSREEDVAKYRRSMIVSGAGILACGMAM YGVIKLDLFGLELQQQQKAQEAAEKKKNNGTMRMDGPDGFTSSPSVIRIQGQDGVEQVTTGTSSVPYFPSTIRLPKYEGDG SSAASKLAPWDELTGNGEDEEEYQLLGLGVRTVSFLKIQVYVVGLYVAKSDISELQQRLVHMAAHPPSDQEVITNQVGAT SATSLVSTERQRLKDLLLDGEKGEDAWNAILKEDGLRTAIRIVPTRNTDFAHLRDSWVRGITTRAQKANARAKAAATEAG AGAANPDEFQDDVFGSAVNDFKTLFGGGQRKHVPKGQTLLLLRNARGELDALFQPDASKPFRFMGRVSDERISRLVWLIY LGGKNVSSEEARRNIVDGIMGIVERPIGTVVQKIL
>FAP_Candida.glabrata.XP_445534
MLRTLVRRSLIKNTGLATRATRLSAVTKRLQSSAPKPATEKSDKKAWWIVGLVSAAFAGGTYVCTWEREELQKERDGKS VLVDDAVTPFPVVISRPTYPLSTKYDILGSGIRSVSVLTFKAYALGIYIARQDKPKVAQVFDSTFMSKNFIDMDENKSHAEN

VKIALDDPEKSRILIDNLLDSNIRMVAKLTPIKNASAKLLKEGIIKNVQMHPDASKNKETLAMGIKEVEEAIKIKGPVPKDDD<br>FLMELLADGSLKFSYYNRRKDIVNELGTVHQPLVGKYLFAQYLSGSNPVSPGTKDQCAETLASLV
>FAP_Capsaspora.owczarzaki.
MSFWTAFRLSGSNVRIAAASFARAGLSRMRPSTAAAAAAAGMSMSTMAAPAAAAAQPRRMRALALAAASGFAAVSIAA<br>AAIAAAKREVVRAEPPNTTQHDTEYAFDKSSGARFPTHLTLANTNLALVGTGVRTVTFLQVQVYAIGLYVPDSAGLKLAIQ<br>RHRDQLVPSTAAETLINLKQPLSIRLVAVRNPDIKHLREALGRAMAARLKHIQDPVERAGAQMAVDQFKALFPQGKIAPGD<br>AVDFVLRPDGVLQLSLRGESLGTVASKFVQTSILDTYIGASSVSLEAQRNFLSGLQAFASQ
>FAP_Clavispora.lusitaniae.XP_002617063
MRTQLPRIITHNVSPHMWKLIRPGLLRAKPTFARKFTSPASFSRVTATIAATSLFLGGSLILNDASNSSLAGVSVDSSIDAFPL<br>ELNNKDIISSKYELVASGVRSVTFVGFKVYGAGIYVQADDHKKLTTILDEYSKLNKGRTVEQLLNDKELSQEIIDDISQKISY<br>AIKITPVRNTDYGHLRDGLTKSLLACPLTKTMREEVGNGVEQLRNIFQGFKGSVPKNDTLWLVAEKDTVTVLHEGTKAKK<br>VEKMGTITEPTMKRVLLVSYLSCVKPLSKPLQENFVSHVSTEP
>FAP_Coccidioides.immitis.XP_001244265
MSSHLPIRRTLCQCVRSNPGSALRHFPQRQQTRLLSSSRSLRAAANPLRNASRSGRKSHTEYKRSIVLSAAGMASCAVAMF<br>GVINIYFPQGVQKDDSKDGIDNGAIKLDGPPGLAPKDDSTVIIDGVEQVSTGNSTIPHFPKSIRLPASLDSPDSRRQSARTPGE<br>EIKKSGEKEEDYYLLGLGIRTVSFLNIQVYVVGFYIAASDMATLQQRLVREAASPSISGVPNETAVTATSLVPKEREDLKKA<br>LLDPEQGEEVWNQILKEGGIRTAIRIVPTRNTDFLHLRDGWVRAITGRAQRANVRAKALATKEHDAHPQSEFADDSFGEA<br>MGDFKALLGGGGGGRKSVPKGQTLLLLRDKVGALEILYQPGDAKPSVWLGEVLDERISRLLWLQYLAGKVVASDGARRA<br>IIDGVMGIVERPVGTVEQMVA
>FAP_Coprinopsis.cinerea.XP_001828427
MSSLLLRSLSFARSACRQSAGVGGAKRTFSSSTAPSSTNGFRRGLACALGLGTLAASVAYARPVHLDAGESAPVVVDPATN<br>IEFPKAITVQANTKLPPLSLVGVGVRTVSFIGIKVYSVGFYADLNNPNLKIPKEMSPEDKVREIIKNTACVVRIVPTRSTSYTH<br>LRDAFMRALQGRLAVAKKEGTITEQQEFEAGGPMRKLKSIFPIAPLGKHVPLDMYLAAPTPGKPRALVFRDLGAIENDWV<br>ATELVLHYFEGDGPSPPLKKSVLERLENFSR
>FAP_Cryptococcus.neoformans.XP_777860
MPPRTARLFAILRSLRPVHAAPCLAAAVGGTLLYAGAESVRLGRWENPLLVHAHSHPQPVFRVDPETSIEFPLSLPLATPSP<br>TLTLVGLGVRKVSFLKIKVYSAGFYLQDGATRCLHHIPGWATFTAQHLLTAPSPSPAGETAAPQLSGEALMANLLDQRIAC<br>AVRIVPNRNTDFGHLRDAFTRALIGRQKLERAKGALSEADEVRITEAIQTLKTFFPAQTVHKGKSVTLLRPPEGGIVVEFEG<br>TILGKLNDPWIGKQLILTYFADNGAVSDKLKEDVAKGLEGFTNKQRAGGQ
>FAP_Cunninghamella.elegans.DY890967.1
NIYAGTLEDPTTKLRFPVHLHSNSEWKRLIGFGARQVSFLNLNVYVVGLYMKSEDIGKLQTLPGWKDFNKTDFLKKEELAI<br>ELLKQPLDVAIRIVPVRNTNTQHLRDGFTRSLLQRMRNQSDSMTEDEEREILDAIREFKTLFVNAKVKKDTEFIFTKTKDGQ<br>FKMEYEDKDMGTVNNKWLAVNFIMTYLNPDTPASELALQDIADGFDNLMNTSPNSTK
>FAP_Cyanidioschyzon.merolae.CME052C
MLNKNSKALWTPLRDYVRRRNAGLSGTVGAPWTVRSNSVQRGCEKENDHRFWHALREGASDLATALRARAPQPSWSRF<br>ADATSTTAYGFAVWTWRQGAPWLALMGVSATLATLLTASAHCERQDAPDATAASPVLGISLETQLGGIKTNEQSEAPSTS<br>HGMDLFKRTLIPSQEVFVGSGVRLMLGLVRVYYVGLYVDPVGARNVLLSDYYGKDAQMICADTAFWDAFRSFRHSVVM<br>HVIRSVDAKHVVQGLERGLTKRLRYAKNKLQMPDDRAKLAQLKNYLLGIRQIPENSQIRFAILDEGNTLMIEMNSVLVGY<br>VESCPALCYAIDDIFLGSKPVSAEAKQSFCTGMERIIKE
>FAP_Dictyostelium.discoideum.XP_643054
MNSLIKSSNNIFRNVLNKRQIINNRVSYRFYSSSNNYQNFNNNNNNNNNNQNNQNNNNNKNYFKDFMAVGIVGGLSLAIPSI<br>AFCVECFEEDDEFITQIKTGFKFPKILSTEYPFNLVNIGSRKLSFINMNVYSIGFYINKDNAKNKLNEHYSNQSKEDFCMNKV<br>EIAQDILDKGIGVTIKIRPTRKVNWGHIYGGFQRSLIIMLLKKYDMPLDEIEVLMNQLKSTLKSTQEISTTESIDFIKNDGLDPS<br>LIILFNNKQVLEIKDKRLANCFFDFYLGDNTKTPEARNQFFENLWSIFNHDQNLKNNIHHTLTNPI
>FAP_Dictyostelium.purpureum.
MLSLLLRNTSRNRQLVRCLSRASSPQRFSHYSFNYQNNNNNNNNNSNNNNFNYKKILTYGIVGIALAVPSIVLCQECFDEDE<br>DFITQIKTGFKFPKILNKDQNILFNIVNIGNRKLSFININVYSLGFYINQEHAQTKLSEHVTKSKEEFCNDKESIKQEILDKGIG<br>VSLKIRPNRKVTWGHIYGGFQRALIVMLLKNYNMTLEEIEPMMLELKESLKPHQEISTSEQIDFVKKDGDSPSLIIFFNEKPV<br>KEIKDKRLANCFFDFYLGQNSKAPEATKEFYENLWNIFNNKQDHHHHQSTLSPI
>FAP_Kluyveromyces.lactis.XP_451711
MLRFTHVLNNGAKRSALSLGRSYLRGFGSMHGPRVAVSTLIKKDKKPNGFRGMLALFVGIGTLAVSGLSTNLYNDQNVKE<br>DPWKSVSVDKSIDPFPTELKAPEFPISTEYVMLGFGIRSVTFISFKVYGLGIYAAKEDLGLIPKVLDSNFLSTAFIDFDSSKSHQ<br>ENLKTALDNPETSRILINNLLDSGIRLVAKITPIRNTDFNHLKDGLVKSILGHPDSKKDEDRLTNGLQQLRDAFSRKGSVPKN<br>NDLLIELQANGYLQVSYFDRKTGESTTMGQVKETLIGKLLFSQYLSGPKPLSPSTKDSVVSKLVTLA

>FAP_Laccaria.bicolor.XP_001873641
MFRQALLSRCRPAQPLRISPRRFASFLFPRPQARNQPRLLLWATAISLATYLTFSPRTVHLDADSQPFPKPSPEDSVVDPATSI<br>SFPKTIRVPSKINIPPLSLVGLGVRTVSFLNIKVYSIGFYADLDNPNLMVPKDLSPEEKIKYIVRNTACVIRIVPTRSTSYTHLR<br>DAFMRALQARLVKGKQEGTLTEDDAQSAASPMRKLKSLFPNSPLTKHTPLDAFLSPPTPGRPRALVFRDLGAIEHDWVAT<br>ELVLNYFEGAVPSPALKKSVIEKLETFEAK
>FAP_Lachancea.thermotolerans.XP_002553468
MIRALARGATIPRSFHYIQYYQKRNLLTHGLKKKHIPPYKTILALGAGAFISGLFMLQKDSITNDAGPIENSESSVEVDKSVS<br>PFTVVLSPPETLLTTKYTLLGFGPRSVTFLGFKVYALGIYVANEDLPLIPKILNPTYLCKAFLDTDSAKSHSENVGAALKDAT<br>KSRVLVGSLIDGGVRFMAKITPIRNTDFNHLKDGLVKSILNHPECQKNQEAVSRGLQELKNAFTRKGSVPKNDDLIIELQAN<br>GSLQLSYRSRKQNECMMLGRVDEPLIGKFLFSQYLGGDKPLSPPTRETFAQKVKTLV
>FAP_Magnaporthe.grisea.XP_360071
MLRQPASRCLRALPRRQQLPQSRTIFSRRSRASPGRAREELNVQRLAQESHEYNQMRRTFLTAGAVAGIISFIYTAYKLKLA<br>IDNKPAKLDSTLPPSDPLLDGSNRKVVLHDDQGRELVPTGNKTVEFFPRTIDLPTVSENRASSAPSDPLAPPIVVNPSTQQTT<br>EYTLVGLGTRSVTFIGINVYVVGFYIATADIAALQSRLVKRVNPIATTLVAGEKGDLHKSLLDPVEGEAIWNELLRDGIPAR<br>SAFRILPVRDTDFHHLRDGFVRAIQARSDKLVDEAGSNAGGDLVKADKDAFGESMRAFRQLFNRGKVPKSKELLLTREDG<br>GRLRVVYDDGKSREVIGTVDDERVSRALWLNYLAGKNVASEPARKSIADGVLEFVERPVGTVAAQVL
>FAP_Monosiga.brevicollis.XP_001743322
MATATGGAMLGVAGCEPPVPGMRVVKDPLSNLEHPAALEAGSLLAPQESLALVGCGTRAVTFLNYYVYSVALYLREEDR<br>RQWGNRYAEPLPEAFREAVNQSPRVIRLTPYRKAAFSHLRDGFNRALFARLHKSSVGPEEDERIRGELQELSQVWSWSYN<br>WSAILIIFALLYESTIAL
>FAP_Mucor.circinelloides.GR548173.1
QCQTSGLDSLGAGLTTAGYLAFSNPAYSEAPAYAGTVEDPATNLVFPINLNTDNEWKRLIGLGVRAVTFLNMNVYVVGM<br>YMKGEDIGALRKLDGWKDFDKSKFLQDTDLAEQFLDQPYALSIRLVPTRATNTQHLRDGFLRLLMQRMKDQDMSEDEER<br>EVLKAIQEFKSNFVSMRVKKDSEFIFTKTKEGGLKLVYEGKDYGTVQNPWLAKNFFMAYLNPKAPSSEAALHDIADGFER<br>LMKEN
>FAP_Neurospora.crassa.XP_961584
MLRQSTFKQLPRSFGSAAKAATRTPASRRTFLSPPRSQPNEHLNRLDVNKLSEQQLKYARNRRAFLWTGLFCSVSSMIYVG<br>YLIKLELKKPVHADSSLPSTDPLAGTNAVERKVVLRDEKGHELVPTGNSTVPTFPRTLELGSFEGAIAPAPGPSASGLAPTIT<br>GPDQTEYTLVGFGLRTVTFIGIQVYVVGYYVATADVAPLQSALTKKVNPIATTLVAGERDQLRTDLLDPAKGEEIWDGLLA<br>NGIPARSVFRVVPVRDTDFHHLRDGFVRAIQARAGKVGDEQFGEAMKDFRQIFNRGKVPAKQELLLIRDGEGKLSVVYDD<br>GGNKKEGRPAGRQLVGTVDDERVSRALWLNYLAGKKVASEPARKSIVEGIMEFVERPVGTIAAQVV
>FAP_Pichia.guilliermondii.EDK39871
MFRQFASVLRTSFRGQKLVSVLNSTSRNHYRPLFATGVAFGLFSAATTSAIFLDAPSNFPPETSVTVDSSISPFPTSLSPEVNP<br>NLSQKYALLGYGVRYVTFLSFKVYGIGVYIANSDIAKARRIVSEALRSDPGSLADPEESAKVIAKLLDEKVSFAVRICPVRN<br>TDYSHLKDGLIKSTLAHSLSKTNRDVVGAGLDELRQVFQGRKGSVPKNHLLYLEVLKDGSLSVSYEDPKKAVVTPMGVV<br>KEPMVGQILMLQYLSGRKPLSEELRRSCGEKLAVL
>FAP_Pichia.pastoris.XP_002490854
MTFFAKCPSFLLLSAEENQLIFYRLVSKHPPVQMFRLLRSRGFTSSFKFRLLATSSVASVGLFTFLQVVPERSLIWSDASLSKL<br>PLPDITESISVDKSVPAFPKNIGGKTDFTLLGYGTRSVTFLSFRVYALGIYIAKEDIPKVQTVLDSKFMSNFTNKTKGSSHFEH<br>VSNALKDATLSSILVENLLDADIRFKVRIVPVRNTDFNHLKDGLIKSILASSKTKEIQREQGDLATQLDQGLDELRKPFTTRG<br>SFAKGNALLLERLPNSSLELSSETYDKQGLLLKQTHLGEIESPIISKLLLLQYLSGDKPLSPNTKEKSCEQLSLLV
>FAP_Polysphondylium.pallidum.EFA78345
MITSIGRNSTKLLLGRNNNNNKYLLRYSCTNSLNNNSNNNYSNNNNNDYQQKQQQQNRYSYTKIIGVSSLALLLPSVVLC<br>KEYFEDHEDFIVQIKTGYQYPKKLINKDNDIEYSFVNIGTRVMSFMKINVYSMGVYIDELEARLKLGSQVERVEQSEELTTQ<br>SELDFIRTKSGELLVLHDQQLLKKIENNRLSSVLFEFYLGDYTKVPEVRQDFFEQLYNIIKNKNDRDITSIGIH
>FAP_Schizosaccharomyces.pombe.NP_593665
MFGTRFFAYQFCKNWLSKRPGLVFWSSVATINAMYQTRPLYCESVTKTIQQTYEGLVQKRINNEDELFLLGSGPRYVSFLSI<br>HVYDIELYIQKKDVQTVHTILQKEVDPKLGLEMSMKDEEIGSRIVAALLKNEMKYAIKIVPTRNTNFSHLRGGFVRGLQSR<br>MSQNDPAEQAAVSKFRSTFPVNRTCFKETALWMKLYGNDFCYIYKDSEIGHMHDENYMVSNLFLKGYLVGPRVNSEQAR<br>ESVCLTLRRIMDGTLLF
>FAP_Sclerotinia.sclerotiorum.XP_001591796
MSFLPRTPRILHTLPRSQCQRLTPIRTHLRTLTHKPATPTPHLDAITLHRLEATQRAHQKRRTIYLATGFLTGMICIWVTATSI<br>DLKPAKLDSTRAHDTLHSSTEVIIQKPHESTPPTPSEPHELIPTGTSTIPTFPKYIQHSSSPDTPPETYQLLGLGIRTVSFLNIQVY<br>VIGLYIQQSSIPLLQSRLIEKASPPNSGASTLVAGEKDFLKKQLLHETDSEEIWTNILQTAGLKTIIRIVPTRNTDFHHLRDAW<br>VRSLTARAQRNRDEFGDELFGRSIAEFKALFNRGSVPKGRELLLNRDVEGRLSVWCEEGERGIRRLGEVRDERVSRGIWLG

YLAGKNVSSEEARRNIIEGVMGFVERPVGTVEGQVHV
>FAP_Yarrowia.lipolytica.XP_502910
MFRNALKSARQVKAPVRAFSTRATAARAVPRPSNTLLFSTAAACAAGLSLYSLYTHQSIALDEKKGPIIQPTDSVQPPTSTPPFPTKMSADGKEWSLLGCGVRRVSFLGFDVYAIGLYLPESQKREVRELLQSTSGFQQANGDVEEFKKSLLDPVHGAAKIRWLLDQGIDIRIRIVPVRNTDFGHLRDGFVRTILAHPEAKEASQNKEFADGLSELKTIFSRKMSVPKHNILVMNRKGNNGELKITYYDAKSEADLGEGSELGTVHNPQVSELLLLQYLTGKKPISETLRDSVINGLVTVAIE
>FAPa_Chlamydomonas.reinhardtii.BG854514.1
MVGFETRDGFAIPTGNAKQSDSLKPENFKFWSARAAPMTPFASLSASILGGLGKPGSAGGRVEPATGYEFPAELCYLKKPCPSLAGLGVRNKKIVIKDIHVYALGIYVDAAAAKSALSGFKKKTAAELEADQSFYDAVVSTPSVEKSLRLVISSRLVDRKKFLDALEDRLAPRLKQAGEPSTLDNFRAQFDSVHFEKGLEIAFTCVDNKKLVTKVAGQEKGSIASPALCSSLFDIYLGADPVSKDAKSSFGKSLAAALKE
>FAPa_Haematococcus.pluvialis.DV203389.1
KILGLKNINIYALGIYVDSAAAKKALGSKFKSLDKETLANSQALLDEVLACESIEKTLRLVISFGALKRSQFVEALNERLAPGLKQAGDEEVLERFKAQFDGATFSKGTEITFTQHGKQLVTKISGQQVGLLTSPSLSKALWDIYAGPDPVSPEAKASFATTLASVIKD
>FAPa_Micromonas.pusilla.XP_003058444
MGVRVKRIAGIGVKVYACGLYVDPEDARAALGDRFVGRDVADVGKDQSLFDGVLRSDDVDKTVRLAFARNIDSAKIRDALSERLRPALGRDSESLKTFETYFDGVTFEKGQALTFSATGGKLETTMKGKSVGVIHDARLCAALFDAYLGRDPVVPSAKRSLGEALAARVVG
>FAPa_Micromonas.sp.RCC299.XP_002503292
MIGFKEHIQGVRADVARRRNERRGEPPVEFRLRPVPTARRLWANAMGGRLGIGSDGNRVALVTEPKTGLKLPGEYCVNGGKCAPLTGMGVRIKRIAGIGVKVYACGLYVNPASARAAVGDRYVGKSVKDVAKDQALFDVVNAAADVEKTVRLVFARDIDSAKIRDALSERLRPALGADSPSLRRFEAYFDGVTFKKGQSLTFTASGGKLITSFGSKEAGQIADGKLCTALFDAYLGKSPVVPSAKESLGEQLAGHVTG
>FAPa_Ostreococcus.lucimarinus.XP_001419318
MSETMGATDRTARAPERGAAATRATTRARPEREQMSPASTRAPSGDGRAWTRADAADGVDGVERPRSAERRRRRRPTRARPGLASTSAPSDAGSTPSSEATAMVTEPRTGLKVPAAMTPGDKALALAGRGARVKRIAGLSVKVYACGFYVDVDGARDGDAASYERLVRLVFARNVGGDKIVEALAERIRPAMDADSPALRAFEAIFDGVSFKKGTSLDFHATFEGELATFIKGKRVSVIADASLCRALFDCYVGKDPVIPELKSTVCAFVDGLN
>FAPa_Volvox.carteri.FD828667.1
ASNVNGLKAENFRFWSAKAAPPTPLASLSSSILSGLGRPGAQGGREEPATGYEFPPEYCYLRKQCPSLAGLGVRSKKILIKDINVYALGIYVDAPAAKSTLSAFKKKPVEDLVKDQSFYDGAVVAAPNVEKTLRLVISSRLVDRKKFLEALEERLAPRLKQAGEPGTLEEFRKQFDGVHFERGLEIAFTCPDNKKLVTKVGGQQKGTISSGALCSSLFDIYLGSDPVSNEAKTTFGKSLAASFHER
>FAPa1_Arabidopsis.thaliana.NP_567140
MVSFRFPFSFSQPPRATTSFSGFSISAVAVSVTVGAAAAGAAIAASRNPSHPILEWAFSSHRSSLSPWGSITLADESVVEPKTGFSFPASIGDSRRLLGVGLRKKSLLGLKNIDVYAFGVYADCDDVKKLVGDKYANLPASEIRGNKSFMDDLMEADIKMTIRLQIVYGKLNIRSVRNAFQESVGNRLKKFGGSDNDELLQSFTSLFKDEYKIPRNSTIDLTKDPGHVLSVAIEGNHVGSVKSHLLCRSILDLYIGEEPFDKNAREDFLDNAASLAFDN
>FAPa1_Cycas.ovule.EX927025.1
PHFAKQRDLLARNLWETCTRRRGASYLLDRFACTSLITDVGQMENTMVEPSSGVSFPAVLDETKQLAGVGLRRRSILGLKNINVYAFGLYADEGGMKEMLGDKYRKLSVAELKGSQEFYEDVMENDLGLTVRLEIVYGNLSIGSVRSAFKESLGSRLQKFGGSQNEELLQRFTTQFKDEYKLQCGTIIDLTRLPGHVLQTKIDDKEVGCIQSQLLCRSLFDLYIGEDSFDKQAKEKIGLSLASFISK
>FAPa1_Glycine.max.ACU23031
MSSMLRFPFSFLQPQRPQFPRPFTAFAAAAAVSSSDRSFLRNALNSFFSVNPSMPLLGSLSLADSGVCVVEAKTGTSFPSILDSSQKLCGIGLRKKNVLGLKNIDVYAFGVYATDEDIKRHLSEKYGKLSASELQGNKEFTEDLMESDISMTIRLQVVYGRLSIRSVRSAFEVSVGSKLQKFGGSDNKELLQRFTSQFKDEFKIPRGSVIHLSRDKGHVLRTSIDGQEVGSIQSKLLCKSILDLYFGEEPFDNQAKEEIELNMASYL
>FAPa1_Oryza.sativa.EAZ04407
MVSLRFPAATFPRLPPPRPPSRPAFAAALAAAAAAAAAAAASLTLTAKSAGRPPLPHPAPLWASISLADGAAPGSVEPRTGAAFPAETSGGRRLLGVGLRRTTILGLKSIDVYAFGVYADDHDLRQLREKYQKLPVSQLKENAELINDALERDIRMTVRLQIVYGRLSIRSVRSAFEKSVGSRLLKFGGSDTHELLQSFVSLFKDEYKLPKGSVIELSRESSHVLKISIEGEELGSIQSKLLCKSIL

DLYIGDDPFDKNAKESVQENMASILKN
>FAPa1_Physcomitrella.patens.DC930902.1
MNAVPGPSFSSRSFWTGVTSFVKNGGVPQRWRAAAGAGAGVVGLGLALTRPEVAPHRKLAAEELWRGLSSWKCDVSRFASIAWAEPVEEVVEPSTGMTFPSVVEEGRLFTGTGLRKKSILGLKKITVYAYGVYVDPASLKSQLGNKYSSENPEELKKNEEFYDDVMVSDVGLTVRLVIVYGSLKIGSVRGAFEESVGSRIKKFGGAANIELLQRFTGAFTDDIKLPKGTIIDLTRLPGNVLQTKIDGTEVGSVQSSLLCRSLFDLYIGDDPFDKEAKKLIGQNLASLLC
>FAPa1_Populus.trichocarpa.XP_002300842
MVSLRFPFLFSQPKKHPNGISRTITSRSFPATTTTVACALAAGAAAFAGIAATRNSKNPKQDNPFIQNALNLLFSNHLLAPWASLSLADPSPSVVETKTGAAFPSVIFESRRLLGIGLRKKTILGLKNIDVYAFGVYADADEVRKVLSEKYGKLSVSELKESKEFKEDFMGGDIGMTVRLQIVYSKLSIRSVRSAFEESVGSRLQNFGEPNSKELLQRFTSQFKDEYKIPRGSVIELSREQGHVLRTTIDGKEVGSIQSKLLCRSILDLYIGEDPFDKEAKEDIESKFASLLQVDH
>FAPa1_Selaginella.moellendorffii.FE510754.1
MPPCRSPGPPAAMAAAAAAAVAVAVNYSYAHLKLSLPPPSPSPFFAAISMALPSPPLTVESSTGAEFPAWIDSDGAKLELAGTGLRKKSILGLKSIVVYAFGIYANAAKLRELKLKDDKKAISLAELPCMDAEMSVRLVIVYGKLKMGTVRSAFEESIGGRIKKFSGAENKELLQSFTQLFKEDIKLPKGTAIDMVRLPGHVLSTRIDGQEVGSVKSELLCRCLFDLYIGDEPFDVAGKEAIAAGLSSMLS
>FAPa1_Zea.mays.NP_001149585
MVSLRFPTAAIPRLPPSQAPNGVAIAATVAAVAAAAAAVAASLTLTGKSTGRPVPHPAPLWASLSLADAAAPSSVEPRTGATFPTEAAVGRRLLGIGLRKTSVLGLKSIDVYAFGVYADDNDLKQQLKEKYSKFSVSELKGNGELINDALERDIPMTVRLQIVYGRLSIRSVRSAFEKSVGSRLQKFGGQDTKELLQSFVAVFKDEYKLPKGSVIELSRESNHVLKICIEGEEVGSIQSKLLCKSLLDLYIGDDSFDENAKDGIHENIASILKS
>FAPa2_Arabidopsis.thaliana.NP_180199
MSNMDPNSVLPKRSFLQHELFSQLHIPGSLAFEAFSCISKFTGALLCWFSHGNLQKEVSKHQWGLTCKSRDSLKHVFEHRNVSVFPFHYVSKDISPGFFGNISKSTIQHFVNEAERLHSCSLLSLAAAMIPSLNVMSANGLALPLGSNDVKLRENIEHRTCPENTEHRTCQVGCEEYSGLSFQKLDWTRQSVEPRTGIEFPMLLKENASRSNSEVLVATGSRTMKIIRIKSLKVYAFGFYVHPSSVCQKLGRKYASVPASKLDKCDDLYKDLLREDIVMSVRLVVNYNGLKINTVRDVFEKSLRARLVKANPKTDFNCLNDFGSFFRQDIPIPAGTIIDFRRTEDGQLITEIGGNLIGAVRSKDLCRAFFGMYIGDVPVSEQTKEEIGRKVVGIIKRC
>FAPa2_Ginkgo.biloba.CB075371.1
GSKAPTLPVLALATAFVPALDNSSSKLLPIPIEDNLDMIEEKQKERHTSLQNHCDLESHGEGDVLYLSRRKCGSIRDVVEPRTGIKFPTALIGTEYGSSNSGLISQVLVGIGVRSTTVAKLKAIKIYAFGLYIQPDCVCQTLGQKYYTVPPEELKNRQDFFEDLLRHDIHMTVRLVVHYKGLKLRMVQNAFESALRKRLRKIKEVADDDGLIEFCAYFSKDISLSPGTTIDFHRMPGGQLRTEIGGKELGTIYSSNLCRAFFDMYVGETPVSLKAKEDIGENIGRVIKRC
>FAPa2_Marchantia.polymorpha.BJ862702.1
GPGAKVDDYSSCQVLAGVGFRSKTLIRVKSIVIYAFGLYVEPNHLRAKLGDKYAGVPPEELKYRPEFYDDLLSQGVGMTVRLVVHYKGLSMGMVRSAFESSLKLRLKRIKGVEDDEGLQDFCSLMSEDLRIFRGTTIDVRWQPGGALQTEIGGHQLGTVFSHHLCRAFFDLYIGETPVSESAKYEIGESFGRLLTNRPHV
>FAPa2_Physcomitrella.patens.XP_001783129
MEGGWLHFPSLVLDFTSNLGFSHSSSCKVPNGNGQCSHFSNPSSKSVSASSSCPTLSENSHLSDVSPLSILSSNSISPVPEKLVLGNGVAAVLHAAGAFLRLLRSRDGDKGSRRRFIKSEKLKTLNDLKAVNYTLIRDISPGIQIISLCTRGVLSSSFFSQISSVLEGGWSKLICYDAAPIVLAAALPPSSLALFQYGQDAFGVDNCDGALFGLPSCNSQAVVVEPKTGLEFPMQLCHNNIDGQDSDASLSCQILAGVGARSKEIMRLKSIKIYAFGLYVHPDHLRGQLGDKYEGLSAEELENGLDFLEDLLRVHEVEMTLRLVVHYQGLKMRMVRNAFHESLRNRLSKIGGNGDEGLQTFCSYLSDDIRLHKGSTIDVRWQVGGRLRIEIEGRRVGVIHSPPFCQAFFDLYIGDSPVSVAAKQEIGSNFARIVKERSRKLSRKYNAVLSRNKSFYIEIDVHQ
>FAPa2_Selaginella.moellendorffii.XP_002987458
MESDTASQDWTSDLTKTVVEPHSGVQFPLHLLGLEGNGLQLSQVLGGVGVRCRQLMKIKSINLYAFGMYVHPESLRAQLGEKYAGVSPSDLKFRSEFYDDLLRHEVSLTVRMVVLYKGLTVDLVRSAFQVSLRNRLRKIKGAEDDEGLQIFCSYLSGDLKIHKGTVIDIHWQPGGRLQTVVDGRRVGTIFSENLCRAFFDLYIGDPPVSSSAKHSIGESFARILNN
>FAPa2a_Oryza.sativa.NP_001057086
MKADRLMLSNLDHDLGYLHKFPSDFPMSHDLGLSLFTHAGTMVGSSLRQHRQICSSGNLIVQEAFDRLNKFARAFCYWLSRVSNPKNLRRLMSMEGPSSGACQSHINHLSSRMQNLAVLQFGYLVREEHAVQLLLANFASTTLGRLWNDFQQQHACNVLTLAGAMAIVPPLENISLKTLAESMALGNIKDYVSRPMDKPYLEDKCIKSRSVAVPSTIFQGDAIEPKTGIKFPAFLEDDSSPSTTVLVGMGFKGVKVMRVKNLDLYAFGLYLQPNTISEKLGPKYASVPTINLKDNPDFYDDLLRENLPMRVRLVLHYNGLSIGAVRDVFEKSLGLRLQKMNPNTDYHCLKTFGSYFNEDIPIPAGTKIDFCQTSDGQLITEIDGRQIGAVKSKDLCRALFGMYIGDSPVSLQAKKDIAQNVAGLIGKC
>FAPa2a_Populus.trichocarpa

LHVPETMAFEALNHVSKLAGGLIFWFSSSNLSRQISGNQRSPSCKSPAQVKNSTSTRDLAGLGFGSISKGESPVVFSKISNFV MRLLHREAERRQSFPVLSLAAALIPSLVIRSSKLLAIPGENDDVPVHASTGQRPCEVERSGCPGLSFPDLNWTRHAVEPRTGI EFPMLLDQNRSRLTSEVLVATGSRTMTIIRIKTLKIYAFGFYVHPNSVCKKLGPKYASVPMGLDTVLIQVLMIIMREDISMTV RLVINCNGIKINTVKDAFEKSLRNRLAKTNPDTDYNCLTTFGSFFTKDVPLPVGTVVDFRRTADGQLITEIGGNQIGAVRSK DLCRAFFDMYIGDIPVSEQTKEEIGKNVACIIGKC
>FAPa2b_Oryza.sativa.NP_001048296
MKPDWSIFSKFDHNGGYLHKFPIDSPISHDIGLGLISHFGTLVESSFQHPRHICSTGNGAVQEAFSCFNKFAGAFYFWLSRAS NPKIFHRLSAIAGSSSRACQSQIKQVTSCMQHLAGLRFGSQVREEHAIQILLAKLANATFGRLWNEVEERHACNILMLAAAT VPPFENISPKMLADSMTLGRDNGRTREPVDQHSLEENHSGCTCVAVPRIILPEDATEPKTGIKFPTLLEDNSNPTSEVLVGM GFRSMRIMRVKNLNLYAFGLYIQPDSICKRLSPKYASVPVSELKDHPDFYEDLLRENIHMTVRLIVSYNGLSIGTVRDAFEK SLCFRLQKMNPNTDYHCLKTFGSYFSEDICIPAGTKIDFRQTSDGQLITEIDGKQIGAVQSKDLCRAFFDMYIGDPPVSVETK QDIAQNVGGLIRRCY
>FAPa2b_Populus.trichocarpa
IANILPNTVLSRGSVLLHELFRFLHVPGTMAFEALNGMSKLAGGLIFWFSSSNLSRQISGNQISPTCRSSAQVKNSTSTRDLA GLGFGFVSKVESPVVSSTISSFVMRLLLGEAERLQSFPVLSLAASLVPPLVVRASKVLAIPLENGDVQVHASIDQRPCEVEHC GCPGLSFSDLNWTRHAVEPRTGIEFPMFLDQERSRLTSEVLVGTGSRTMTIIRIKSLKIYAFGVYVHTNSVCEKLGPKYASIP MGSDTVLIRVFIMILREDISMTVRLVINCNGIKINTVRDAFEKSLRNRLLKTNPDTDYHCLTTFGSFFTKDIPLPAGTTVDFRR TADGQLITEIGGNQIGVVCSKDLCRAFFDIYIGDIPVSEQTKKEIGKNVASIIGKC
>FAPax_Chlamydomonas.reinhardtii.XP_001698296
LSQLWNRARQPHHAHAQPHQTSHHQATQLCTCGGASTGMDCSAACAASAAADPDLLSQPLHAKHGHGSHSLQHLLATG SRQWGSSSWWDGRSMLKVKIYDFALYADTTKAREVLRSGLPSFAPGGTGSALIARSSSGGGGGSPTDLLVPPASGVGLSLT IRACRNLPLPLLGAEFERILQRRHEKAGGRADDPALRELLSYFSRERLPAHVVVGGGPGNSSEPAVRKGAAITFSRSSSGEL VTEAGGALLGRVRSPALAEALFDLYMGEMPVSKKAKAAATTALLQLAEGGDSPYRLQPGERLLCAPGASPTSQPNACVL ASS
>FAPax_Coccomyxa.sp.C-169.GW220737.1
MEVESDYEEKRRLRGPLAPLRLFLSSGRAVSKAGKWGLEKVRRRGRTGPVMGSVTAEHFNRESEQNLQDRWQWPQGLS GLGPLSFLQPRRGPAAGAQGEQHGRDCFPAVLSNSMDGGAESTSGRQELVTVGSRQWGHPAISSLLRVKIYDFAMYMDG KQARESRVAEQYKGRQKAAPLDSTFFQRLRSSDDVDMALMVRASRNLPIKLLAGEYERILRRRLAVVGGSPDDAALAQM VTCFREESLPESIKAAGSVRKGTVLTFTRDHAGTLSARADEHELVTVQSRPLCHAVFDLYMGDQPVSRKAKRIAGESFQRL VEGQDGEYQPPRLQLVCDDSLGACEL
>FAPax_Volvox.carteri.XP_002951924
EFERILQRRHEKAGGRADDPALRELLSYFSKEKLPEHVLVTSSSVSSSPSGGVGDAVRKGSSITFSRSSSGALVTEAGGRVLG RVESPALAEALFDLYLGDQPVSKRAKAAAGAALLDMAAAATDGGSGGAVPTRYRYRPQPGEKLVCGPGAASGATSPSW GGLIDVKACILEVP
>FAPax2_Chlamydomonas.reinhardtii.DC847756.1
ALSPSVQVADAPAGAPGGGDKAGALGRRILSQLVAPVNGVPPMDATLMLHAARDLPVDQLGQELFDVLNRRSIKAGGAP NDPALQRLIGFLSRSSIPASTLTPDKVHVRRGSSIVFSRRNGSPLV
>FAPb_Aquilegia.formosa.DT759216.1
WFSTKKPFSHTLSLPLSTYYSTSCYHKSKRLLFSPKSAADIYSSSDEDEDLIVEPNTNVKFQSVLSVPGCPTPLSLLATGYREK VFAIVGVKVYAVGLYTNVSITKSLKAWKSRTEAEFQKDSSFFNSIYRAPTEKTLQIVMVRDIDGKTFWDALDSAISPRIKAP TPADESALSTFRIIFQQRSLKKGTLVFLTWLDPSKMLVSISADGSPSTVDATIEATNVNLAFFNIFFGDAPVSPSLKAAVCKGF AQILR
>FAPb_Arabidopsis.thaliana.NP_175757
MDGILAAVPSAVCVSLRISCRNLDNAESIYHFPGKSLNRVSVLQTGNYVSRKGNSLLKNRHCGEISRVIVKSAASSVGNAED YAEETATSVKFKRSVTLPGCSSPLSLLGTGFREKKFAIIGVKVYAAGYYVNESILSGLSAWTGRSADEIQRDSSLFVSIFQAQ AEKSLQIVLVRDVDGKTFWDALDEAISPRIKSPSSEDTTALSTFRGIFQNRPLNKGSVILLTWINTSNMLVSVSSGGLPTNVD ATIESGNVTSALFDVFFGDSPVSPTLKSSVANQLAMTLV
>FAPb_Chlamydomonas.incerta.EC112863.1
MQKSLAQRAGRQAFRAARCPAALRSRPAAVSRGACQPVRASLTVKDAQSGVEYLLAQKFWQGEPYRCLGAATRSKNILF VNVKVYSITAYVEADRAAKELGVRQRGGFFESDNDYASALVDGAFNKVIMLHLVRDVTGEQFTEAINKSLLPRMQLAGD TAALDKFNDYFNSKNLVNNTEVILMWSMAGDLEVLVTPPVAAPQEYGTATPELRISSAALGRGLFEIFLG
>FAPb_Chlamydomonas.reinhardtii.XP_001696959
MQKSLAQRAGRQTFGAARCPPAALRSRPAAVSRGACQPVRASLTVKDAQSGVEYLLAQKFWQGEAYRCLGAATRSKNIL FVNVKVYSITAYVEADRAAKELGVRQRGGFFESDNDYASALVDGAFNKVIQLHLVRDVTGEQFTEAINKSLLPRMQLAAS

LDKFNDYFNSKNLVNNTEVILMWSVAGDLEVLVTPPVTAPQEYGTATPELRISSAALGRGLFEIFLGDSPVVPEAKTEWIKGAKQLLDSENVKRSTRKA
>FAPb_Chlorella.sp.NC64A.EFN53544
VKVYAVALYVEAEKMARELGVRNRGGFFDGDDDFCQALVDGGCVKALQLELARDVEGAQFVQALEEALAPRMRLMGDTASLEAFKKVFVDKKLAKGTNVVLMYRTDATLDVAVRPGRVDWSTIVADASIASAGLCRGLFEVYMGGESVVADAKKEWANGARKLLESDDIRRQTRKGGSG
>FAPb_Coccomyxa.sp.C-169.GW225238.1
MYAPVHNCTNINSHTARLIRSQSSDRPKVPCVLPQQNLSRCRQQLRSPLARCHPLARRGGVSVQCSSGLVVREESTGVEFPEVTTLWEGGKMRSMGAGVRAKKFAFVPVKVYAVTVYVEAEKAARELGVRQRGGFFDDNRDEDFTLALVDGAFAKALVVQLVRKVEGKQFYEALEEALAPRLRLAGDTGSLAKFGDFLSGRSLEKGTAIILFYRVEGVLEVALMPPGSSDYSQAKPELRVESPMLCRALFEVYMGSDSIVPHARAAWAGGARTLLSSEQVRRDTRKSGS
>FAPb_Glycine.max.AAT94361
MLGAVAASTSLCFSPSTYSTRIVVLRRISNSTLALNNGHSFLLLSAKPMHFSSHKSSRRQPHFLAQAAASSAVNAEYVEEPATNVKFQTSLSFPGCSNSLTLFGTGYREKVFAIIGVKVYAAGLYLDQSIALELNAWKGQSKEAIQGNSSLFETIFQSSFEKSLQIILVRDVDGKTFWDALSDAISPRIQQPTTTDETALTTFRGVFLDRPLKKGAIIILTWLNPSGLLVSVSSNGLPSTMDATIESANVASALFNVFLGDSPVSPSLKASVAESLSKVLK
>FAPb_Ipomoea.nil.CJ754196.1
DSEIKGQGKKLEENGRKNDTPYLILLGLGEFAFLLPSSLENKNMAAVGSMPSSLWISVSAPTKSKITSCNVKPRFSLPLSKTHSYFTIFSTFPSHFSLGSLDARLPGLFSPRAASSSSVGNAEYVEEPTTNVKFQRSLSLPGCCTSLSLLGTGYREKVFAIIGVKVYAAGLYVNESILNKLDAWRGRSAAELQNDSPLFETIFKAPLEKSLSIVLVRDVDGKTFWDALDEAISPRIKSPSADDETALSTFRTIFQGRPLKKGTFIVLTWVDPTKMLVCVSSDATPSSIDAKIESANVTSALFDVFLGRNPVSPSLKASVANGLEVVLK
>FAPb_Micromonas.pusilla.XP_003059642
MSATHAALAARRAVVPARASASRRARRVSSRRGRATTTTTRAAVTEPATKITFEDALPSAVKDGASLTCVGAGVREKKIAIINVKVYAVALYVDADACKAALTTGATPLDGAFDKTLAIELARDVGGETFWDALEDAVTPRIRRIATDMATKEDEDGNFMATVAEAAEVAEEAAMDGAESLKGLFAGENLKKGTRVTIAWRPNASDGGDVLCVSVGGGKSIASASEELALALFDVYLGDDPVSDAAFNAFTKGVAKLVAPF
>FAPb_Micromonas.sp.RCC299.XP_002504118
MTIPASAARVVSPARRDVRALRSRAGSRATFSKQARVNNEIVARAFSIVEPATKVSFPKTFNPHGGDTMQCIGAGVREKKIAIINVKVYGVAMYVDAAKCKEELANGGSLLTGSFDKALLVQLVRDVDGKTFWEALDEAVGPRIRRIATDMATAEDEDGNFMASVAEAAEVAEEKAMDECEEIKGLFQGRKLRKDDKVLIWWSPNKGKFEIGVVGGMPLELTSQQLAQAVFDVYCGDDPVSPSAFQSFVAGAAAL
>FAPb_Oryza.sativa.CT853638.1
MSVVPVVSSALAGPAIAVARPASGSVTRLRAPHGVPAGSVPLASGLANCHRGAAVAAARRLPISSAAGGGTVGDAFVIEDTINVKFPREIAVPGYTEPLVILGTGYRERFFLKIYAAAFYVDCSIGVDTMRWSEKVGIETFDASSVFVSIFKAPVVKSLSIILIRDVDGKTFVKALDDIIARQIKKPSAEEEQGLSTFQKTFLGRSLKQGTTVYLTWLEPSRLLISISGNQDPCQVDAEITSATVNYALYDGFFGSSPVSPTLRSSTAQLLEAILTK
>FAPb_Ostreococcus.lucimarinus.XP_001420321.1
MLRRALLRSRSSIALNASRALAPSFKARALSTDDDAKAKTRAQAEAEAAKKINAGASPLFSWTEVDVSNADRTIPRWQNAAFVVVVGAFFAYFGNKLTSRAGRRAARGIRAAATDPATKISFPDTNSAGLTVLGAGCRVKRVAIIDVKIYALAMYVDADAARAQKGKGLLNGDYDKELAIELARDVDGKTFMEAMDESLGPRIREIATNMATAEDEDGNFMASVAEAAEKAEEAAVDSLDAMRDGFSSLKLKQGTKMTITWTSNGCAIAVAGAAKMEFESAEFAKALLDVYVGEGPVAPAAAQTFEKGLAAL
>FAPb_Ostreococcus.tauri.XP_003082012.1
MLRRAAERCRSLARATLLDVHHPCASRDVSHTVNARRKAEETTEQKELRARAEKAAERKIRASDSPLFNWVEVDVKNDDTEIPRWQNAVFIVVAGTFLAYFGHKLAKSELERRARATAAKENLESRARAVIRERMREGRSVADVAADDAFEGLAPEEIDALVSRERDGERGAHAGLSQARAIRGRNDRRTSRTTTRAAVDPATKISFPDATPSGLVALGAGARVKRVAIIDVKVYALCMYVDAAKAKDQRGKGLLAGDYDKELAIELARDVDGKTFYEALDEALKPRIAEIATNLATKEDEDGNFMASVAEAAELEEEKALDALSDMRDGLIKLNLKQGTKMAISWTPKGANKVGIEVQGATKLEFDSTVFAQALLDVYVGSAPVAPAAAQAFEKGLASL
>FAPb_Physcomitrella.patens.DC956762.1
MALLSCRAVSQSISISLSSVSVAASKPASRTNATKCGSARLQSSLVAGSQLGTSSILSVTAKSKVSVNKVLGVRAAVGADNVVTEPATSIKFALLLTVPDSSNTLTFLGAGVREKQIAFLKVKVYAVGVYAQPDVAASLASWKGKSAADLVKDEALFQELAQAPVEKALQIKLARDVDGATFWGALDEALVPRLTASGAGADGDAALAEFGNVFKNRSLQKGYVITLTWVQPSTLRIAVAESEAANLKTEASIESKALLSALYDVFLGTSAVSPSAKAAVAEGISKLP
>FAPb_Selaginella.moellendorffii.FE511008.1
EPSTGFGFSNLEKLPGASCELSLLGLGVREKKIAFVAVKAYAVGFYANKEQLXXXXXXXXXXXXXXXXXXFAGAFEKGL

VVKLARDVSGALFSSALNDELKPRLKSSPEGEKILSEFGKLFQNRKLKKGTTVYFTWIQPDTLYVGVSDGETPATPNSTFVS<br>GFFASRLFDVYLGEKSVSPSLKASIADHLHV
>FAPb_Volvox.carteri.FD813973.1
EGVSRQRISVVPGYIETQDFSGLLEDRCKMQQSCRTSLRSARPAFGSRPFQAVFRPSYAGRRTFVPVRASLTVKDAQSGVEF<br>LLAQKFWQGEAYRCLGAATRSKQIVFVNVKVYSVAAYVEADRAAKELGIRERGGFFETDDDYCSAILDGAFNKVLALHL<br>VRDVTGEQFTEAINKSLAPRMQLAGDTASLDKFNAFFSSKNLVNNTEVLLLWSMAGDLEVLVTPPVTAPQEYGQATPELRI<br>SSPALSRGLFEIFLGSSPVVPEARAEWVKGAKTLLESENVKRASRKA
>FAPba_Marchantia.polymorpha.AU081874.1
LVAVTVALEQGGRRHLWLHDTRKMFQWCFTLTAAIQSFGVQTISAFARQNDRRGTPRLSYTAPVYPSIQPQRTPALTGFLG<br>HLLIAQSHARVAFAEAPSSTSMSTMPTEESEGVEKEVVEPVTGIKFAKSTNAPGASKELVLAGAGVRIKKVFAAVKVKVYA<br>VGLYVDPSAVSSLQAWKSKGTKEIEKDESFSKTLIDLPVEKSIRIVLARDVEGAQFWGALGDALAPRLKALG
>FAPba_Populus.trichocarpa
IMVSIRISCTNLNNSKILHHFPLKSPNRVCVLGAGWGPLYLGYKILRTYHCGKIMRLLFSPIAAVGSAEYTEETATKEKFQRS<br>LSLPGCSTSLSSLNSGYREKVFAIIGVKVYAAGLYVNPSILSTLSTWKGQSASEIQENSALFSSIFQAPLEKSLQIVLVRDIDGK<br>TFWDALDDAISPRIKTAIPVDESALSIFRSIFQGRSLKKGTLIFLTWLNPSKMLVSVSSDGIPSSVDARIESENVTSALFDVFFG<br>DAPVSPSLKNSAANGLATIL
>FAPbb_Marchantia.polymorpha.BJ845865.1
MAASSVTMACLTSVSVVKVPDSQYPSTKRHCPTAPVLPSIQPRRTPYRSGFFADFPNAQVHSRCAFAQASSSRKIPTLPTMA<br>SLRVEDEVVEPVTNIKFPKSTIAPGASTDLVLAGAGVREKKIAFLKVKVYAVGFYVDPKGISSLDAWKSKGTSELEKDESFS<br>KVLIDIPVEKSLRIVLARDIEGAQFWGALDEALAPRLKAA
>FAPbb_Populus.trichocarpa
MHGVLASVPMLICVSIDLTTFNVLNSKSLYIFPCKNMNREEIPESGHYENRKGGEFLGMKCCNEIERIIVRDPVAAVGSAEY<br>TQEPATKERFQTSLSLPGCSTSLSSLNSGYTEKVFAVIGVKVYAAGIYVNPSILTTVSTWKGQSASEIQENSALFSSIFQAPLE<br>KSLQIVDGKTFWDALDEAISPRIKEATSGDKSVLSTFRSIFQGRPHKKGTSIFLTWLDPSKMLVCVSSDGTPSSLDATIASEN<br>VSSALFDVFLGDAPVSPSLKTSVANGLETIL
>FAPd1_Fragilariopsis.cylindrus.GW082127.1
MLFFASSRQSILISSQSPAASRWCLAAAQQHAATAGAIRAPRIVRSYTRRALSAATATSSHQAAARRTAFVAVMGVVAMTI<br>DYNNHTNTGVLSVGALPSHSSLSRCDSSSPTTTTTRTTTTTDCDAINVGKITTEPITKILFPALVNGMSLAGVGVRVKYMFV<br>NVYAVGAYFDPIAMMAIKQGTTTDIETALVDPTYPRTIRIVMNRGLSVDKFINAIVEAVEPRLKGKNLDTLDEFKELFPKVD<br>LTEGDEVEMTIRGDVLLLKTGLGVGTIRSRPFTEAMCDVYFGKDAVSPTLKNDVLKGIPNL
>FAPd1_Phaeodactylum.tricornutum.XP_002182851
MFATAVSRLSSRFQILPRPIYRRTLATLVFGNNAHAEKSASTATWWVVAALGGLTLTSKTLLDIKIPRGGDVIAAGTPVQEK<br>ATGILFPQLCNGYYIAGCGVRVKYGFVKVYAVGSYFDPLAMSAVKKGSRESMEQALLDPTYPRTIRIVMNRGLSIEKYTSA<br>IVEALEPRMKGQDLEKLEEFKQLNPAVDLVQGAELEMTIRGDTMLYKNAVGGVGTIRSRVFTEAMCDVFYGSDAVSPTM<br>KQSVLEGIPNL
>FAPd1_Thalassiosira.pseudonana.XP_002297476
MMLSSSRVLCHQGRILTRQSTRRITTSSKSTSLSIAIAAYTTATASILLYNLHSSHDVTNSSHNNYRAAQCSAPLGGEAVMLS<br>PKTEPGTGILFPRLCNGMTLAGCGVRVKWGFVKVYAVGTYMDSLAMSVIKSQGEKEVKKALLDPNYPRTLRIVMNRDLSI<br>DKYTSAIIEALEPRMKGQDLQSLEEFKKLNPPVDLIQGAEMEMTIRGDTLLYKNAVGGLGQIKSLVFTRAMCDVFYGEKA<br>VSPTHLEDVLKGVKEL
>FAPd2_Fragilariopsis.cylindrus.GW076569.1
MMMIQRYALLCSLLLVSTVSAMKDAATGIPFDSKSSGLSLFGVGVRKKGPIKVYSVGMYCSDNVQEDLSSISRSDSKDKK<br>AIAALRSGASSNPTTFVLKMNFKVGAEKMASAIADSVSPRHFSDSEVSQLKDLIFEGVSGKGAAVKGTVLEFDCTDDGVEV<br>KVDGKGYGNVPSDALGKAFRDVYLDDKCVSPKLRENILENCCAL
>FAPd2_Phaeodactylum.tricornutum.XP_002181351
MVRVWMTLALFLSVTMTAASAMTDKATGITFAPKKNDLEIFGVGVRKKGPIKVYSVAMYCQNSVKEHLNLISRSADKGK<br>AAFKALRHGAHEASSTTFLLQMNFKVGAEKMASAIADSVTPRYANTAEVESLKSLIFKGVAGKGAATKGTTFEFDCSKEG<br>VHVSVDGKSQGKVASDGLGAAFCDVYLDDKCVSPPLRESCLETCFAP
>FAPd2_Thalassiosira.pseudonana.XP_002286126
MTSRSLVFKVAAFLAVALSIAPSSVHAAAALIDSATKIQFDDTLGGLSLFGVGVRKKGPIKVYSVGMYSDDETKASISSIPKS<br>NKDGALSTLRTSLKSATATTFLLKMNFKVGAEKMAAAIAESVAPRTSDKAAVEALKKLILDGVATKGAATPGTVLRFDCS<br>SETGVKVSVDGKEIGAAPGLCEAFCDVYLDDKAVSPALRNSCVENCCT
>FAPd3a_Thalassiosira.pseudonana.XP_002295280

HGLLSNTNRLRLLRGGASPTSSTTAKLDAIESKLKPLQDAATGVSFSPKLDDLYLVGVGVRKKSIIKIYAVSMYASPSVLNALSSLPSGKQHRKEAAAALRNAARLFDNASNAKTTFVLEMVYGVDAKSIASAIGDSLKPRYGGSTSDIQHLESLIAEGLSKKGGQASKGTVFRFDCSEDGVDVSVDGKLQGSAKFKGMGSAFVDVFIDDNAVSPSLVDSCIDNLSG
>FAPd3b_Thalassiosira.pseudonana.XP_002296145
AEEDELARAAEIEELRRQEEEAAEASAAAKQQKVDVVESKMQSLKEKATGVTFDAKLDDGLYLVGVGVRKKAIINIYGVAMYTSPAVLEAVSAFQRGKQKLDAQNALRNAARSFDSATPKTTFVLEMVFKADAKTIAGAIADSVKPRYSGAASDVNELESLIFEGVKSKGGTAIKGTVFRFDCTESGVTVSVDGNEQGQVESEGIGSAFVDVFMDDKAVSPQLIDSCLDTWCESGL
>FAPd3c_Thalassiosira.pseudonana.XP_002295280
ESLRSFAVTMNLVNINAGSSRYTPFVSKTRQSANKNQWKASSVNPFISELSKSRAKQSADKALLYAQGTVQSKEAAKYHLFDRASSYLYNLASIIDGNSSANHYYITASDNDETTDSDFSDEVEGYAEETEDAADKLEEETSLNEESVAKLKAIESKMKPLQDYATSIIFAPKLDGLYLVGVGVRKKSIISIYAVSMYSSPKVLNAVSSFPPGKQHKREAAAGLQNAARSFSYQSSMTSFVLEIVYKVDAKTIASAIADSVKPRYDGSISDVEVLKSLIFEGVNSKGGVATKGTVFRFDCSEEGVSVSVNESMQGTARYRGMGSAFVDVFMDGNHVSPTLVDSCLDTWSGVDLS
>FAPd3d_Thalassiosira.pseudonana.XP_002295280
AEETEDAADKLEEETSLNEESVAKLKAIESKMKPLQDYATSIIFAPKLDGLYLVGVGVRKKSIISIYAVSMYSSPKVLNAVSSFPPGKQHKREAAAGLQNAARSFSYQSSMTSFVLEIVYKVDAKTIASAIADSVKPRYDGSISDVEVLKSLIFEGVNSKGGVATKGTVFRFDCSEEGVSVSVNESMQGTARYRGMGSAFVDVFMDGNHVSPTLVDSCLDTWSGVDLS
>FAPd4_Thalassiosira.pseudonana.XP_002290658
VVLAIVASIVGPNILSHDVSTNGQSVFIDGIAFPSSISKQTLIGGGTRFKWGFKVYGVGIYGEEKTVQKLKKQYTTEIPPALFEDFSQSKAAKTLLLRFHREVASSDVAEALGEALKPKVGKQTSDAFETFILNMIGGDILAKGSDIFIACKGEKVTASLTGGNASSSMNVKGLCPAIFMVYLGDNPVSQQAKEGFAKGFSSM
>Flavobacterium.psychrophilum.YP_001296794
MKNFIFSFFILFLFFESNVVTAQTQFETDGVIVPRTIPFQNSTLQLNGFGTRSKMWVDVYVQALYVTSLSQDAQYIINGETEMAIRIQIKSSMVSSAKFSKNFDKGFEQSAGKDIYSFKPRIELFKKMLKNKIVKEDIFTLVYNPKDESVWVYKNNDLKGKIPGLDFKKILFGIWLSDNPVDEGLKKELLGKE
>Francisella.tularensis.YP_763169
MLRKLLIIIFISLPLFGVAEELTLQQIKSQQVGKVHFSKWFFDVYDAELYSENGHFSWDKPFLLKIHYLRSFSGKNIANHTVKEIAEQHPQLAHTTLDKYKEIFTRLMPDVKNGTNLYGYMDKDGNGYIYSDKGLLGEIPNKTLSKYFFEIWLSDKSSHIKLSKQLRGL
>Gallionella.ferruginea.YP_003847632
MKKKLLCAMFLLVSHSTQAVELKGVALPDVVHLGNSNLVLNGAGVRSKFIFDLYVTALYLNAKKNMATAVLSDLGEKRIAMYFLDDISAANLLYSFDEGIRDNHSATELAAMKEELHKFDVICHRMVRLKAGDVILFDYQIGTGTQVWVNGGLRGTIAGSTFYNTLLKIWLGEKPVQQDLKLKLLGGF
>Geobacter.uraniireducens.YP_001230365
MRKLVVVLLLMLAAVNAHALEVAGVHLETAVIVNNHPLKLNGYGIRKKFFVKVYIGSLYTAKAVAKADEALSEPGDKLIRMNFLHSKVDKGKITEAFSEGFAANSPQLAGSAEVKKFLAFFTSDFSKGDTVDLSLGSDGRVVAVHNGKVLGTITSKRLAKGILAIYLGEKPADEALKKGMLGRES
>Haemophilus.influenzae.ZP_04464277
MKMKSLFVAMITFFSTAPFAHWQPIGNAEYTWGPFHVYTIGLFSETGTYQENERPLMLSFKYEKPIEGKNFAITLIKEIETLKLNDGDTQSWLKEMQETFPDFSPNDILNYIALPDKGYFVLNDTVLEHDFDAKFNQAFIGIWLAPNSTFVKLQPQLLGKTKSNHEAAEFYLKPESESFDEQESMPELPPHYLLDSQKNSQG
>Halothiobacillus.neapolitanus.YP_003263764
MSSHSIATLGRSRLLASTLLVLSLGASNPVLASNETMPKQMQLGQTDLTLLGKGTATYLWFDVYDAALYAPENIKPAQILSQETPRVLVLQYHHAVTVKDIEKASWQTLDKQLTPSELQSIKPQVDALQSSMKNVSPGDQYTLTWQPANAQKKPELTLKLNDKIVFKSDNARLAATYFGIWLGKPPLSQSLKRSLLGG
>Klebsiella.pneumoniae.YP_002240698
MRFALLLLWLTTLAPAAHAADWLTWRRVGEATLTWGPFTVYHSQLRTPNGRYDGPQQDRALIITYRRDIDRDALVDATRDQWQAQGILQQEPRSEAWLRMLQGIWPDVAPGSQLAFVVSGGEGQFWYRASAAQTAFTPLGPRQSAAFSTRFLAIWLDPRTTYPELRQQLIGGTP
>Labrenzia.alexandrii.ZP_05117176
MFSFGSGPAHADLGAAARSVPSASLVGEGRMKFLGFNVFDAELYAPNGVYSSSNPFALRLTYLRNFKGKAIAEKSAEEMAKQGVSKAQLDGWTKQMTAIFPNVSSGQSITGVRTASGSSVFYLGNKEIGTIADPAFTKHFFDIWLGSNTQNPRLRAKLVGAGS
>Marinobacter.algicola.ZP_01895894

MKKALSTGFASLLLTAALAAPAAALTVEGVDVPDTYSAMDTELKLNGAGTRSKWFMDLYIGGLYVPETIDDGQTIINADE<br>PQAITLHIISGMITSDKMKSATMEGFENSTGGDLAAIKDDVDAFLDVFSEEIKDGDVFDLVYLPGEGVRVLKNGDQRATIG<br>DLKFKKALFGIWLSDEPAQEDLKEKMLGQR
>Methylibium.petroleiphilum.YP_001019430
MERRACLQLLAAAACAAAVPALRAQATARPPAEALAELPGARLQGSGRLRYFGLHIYDARLWVGPDYPATAPSREDYAA<br>HRFALELEYARSLDGEKIAERSIDEMQRAGPLPDAQRQGWLAFMTTTFPNVVAGSRITGVHRPGEMARFYFNGQPAGELR<br>DPTFAQRFFGIWLGPQTSQPALRRSLLGGTP
>Myxococcus.xanthus.YP_633103
MKATLTAVVLSLMFAAPAFAKEVAGVKYPETASVAGKELKLNGVGLRSKLVFKVYTAGLYLETPSKDGAAIISSDQIKRV<br>RMYMLRDLDKKTIVDAIGDGFKKNAGSKLPELQPKLDTFNAAIPDVKKGDELILTYIPGQGTQVSSKTGKEISVEGKDFSD<br>ALFSVFVGKSPVDGSLRDGMLGKD
>Polaromonas.naphthalenivorans.YP_983872
MTLTKRCVTTLAGLLLTVGIQAAPVEMAGVKLADPIDLGDARLQLNGAGIRYKAVFKVYVAALYLNKKAATPEEVYATS<br>GPKRISITLLREIDSNELGKSFTKAFEENAPKTEMSRLIPGLLKMGQVFADQKKMVAGENFTIDWVPGTGTVISVKGKPQGE<br>PIKEAEFFNAMMRIWLGSKPADWKLKDELLGKAS
>Pseudoalteromonas.atlantica.YP_660905
MKKLLTFTFISALLLPLSLSASPIDALKPVGSAKLSVVFWDIYQSTLYSDDGTFTSDDLTQESQSPLALNIQYLRDIEADELVE<br>ATADEWNKLGLGEAIYQPWLTQLTAIWPDIQENDELLFVLNHTDGGVFYFNQEEIGRIDDRSFGVNFLRIWLDKKASYPKL<br>RNKLIGQSQ
>Pseudomonas.mendocina.YP_001188588
MAERSLHLLSALLLSLALATTSVHASTQSGWRERLPQASLIGSGDFSWFGFSVYNAKLWSPSAKVDFDQPFALELTYRRNI<br>SRDTLVDTSLDEIRRIDAQALQAGREAEWARQMRQAFVDVQDGTRITGVYLPGEGCRFYVDGQLQHVVDDPEFARAFFSI<br>WLDPQTRSPKLRAALLGLNR
>Pseudomonas.putida.NP_744873
MTTYSMSNSFYLDFHGCSITGARGLQKCACWALKTIHGSSNICTSVYYCIVFSKVLEVRMAHPARWTCLCLLLAAMSSAA<br>DWRAELPAAQRLGAGDFTWFGLRLYTARLWAVGPVQDWNQPFALELLYHRSLSRDTLVKASLEEMQRLGGGSMTAQQR<br>AAWTRAIEQAFVDVSPGMRITGLYLPRQGCRFYVDGKLSHAIDDPVFARAFFAIWLDPRARDPQLRQRLLGLAGDDRGA
>Pseudomonas.syringae.ZP_06459501
MRAPRWLWLVMLLSAGASADWREALPNAQVVGVGDLSLFGFRVYTARLLSPAKPFVADAPLALELTYHRDIDREDLVD<br>ASIDEIKRISGSRVNDQQLAQWRQQMNQSFVDVKPGMKITGVYLPGREARFYVGDKLQHVVPDSEFAKAFFSIWLDPKTR<br>NPELREQLLGSTGS
>Psychroflexus.torquis.ZP_01252671
MKFIYVLMMAFLMTMPAIAQTEVAGVNLPNTLEVGETQLTLNGAGVREKFWMDMYAGGLYTNVKMTEASKVMNEDAL<br>MAIELHIVSGLISSKKMSSAVEEGFQNSTKGNTKPFRPKIDKFISFFSDEISVGDVFDITYLPSKGVVVFKNNKELGTIEGLDY<br>KKALFGIWFCDKPADEDLMNGMLSL
>Ralstonia.eutropha.YP_294806
MAYQVVRLPPMLHRRNSPSSGPLSAPHPLSRPRQTPRRLLAMLAAGVAFGVLVPAGATEIEGLRFDDAARVSGKVLQLNG<br>TALRSGLLLKGYVAALYLPEKGRNATVVLGTPGTKRLQLRMLREVEPTALSRAIQKGMRENHSELQMQMLSARMVQFEH<br>TIDQVGTARKGDVINFDFSPDAGTVVAINGTPRGRPIPGDDFYQAVLRVFLGEHPVDRDVKRGLLGG
>Rhodoferax.ferrireducens.YP_521361
MSTRNSLKQLLAIGLLALVSVLPSWAAEVGGVKIDEAVTVEGQPLKLNGAGVRTKFVFKVYAMGLYLTDHKTTPSDIQAL<br>AGPKRVTIVMLREVASDELAKTFVAGLNNNSNAAEKAKVSDQTQKFEKMFTTVPVVKKGDVITLDWLPATGTVSQLNGK<br>PLGEALPDAAFYNAVLRIWLGEKPVDDALKSALLGAK
>Silicibacter.lacuscaerulensis.ZP_05786044
MRDLIQASLSRLRPARNAAILSLVLLAPAGLAATPVTKVLPDAELRGTATFRFLGLPIYDAQLFTKGGGPLNWSQDFGLKLT<br>YRKSLKQKALIDSTLDEMARQGNSAPIEAQLNTCYQAVGAGDSYLAVTKGPDAIEFWRNGRKTCTLSYPGIKRSFMSIFLG<br>NNTRSAAFTRRLRGQ
>Thiomonas.intermedia.YP_003644776
MDHTAHKPLRTFGATASLITALAIASFMPSAQALTLHGVDIPPQVDVGGKTLTLNGAGTRYVFFFKVYTAALYLSQKSTQP<br>QAIYNMAGPKELKLTLLRDVSGKELGDKLNEGIKNNLSPEEFSAFIPSLVQLGGLFAQKSQIKTGETVTIREIPGKGSTIAIDG<br>VPSGGVFTEPQFFNSMLKIWLGKNPAEDRLKRALLGNEADSN
>Variovorax.paradoxus.YP_002942501
MPAALSLPTRLFSRLAVLACAWFALGASAAQVDVAGVKLSDTLDLRGSTLQLNGAGVRYKAIFKVYAAGLYVEKKVSTP

EEALAAPGPKRVAITMLRDIDADELGRFFTKGVEENSPKSEMVNLIPGLLRMGRMFSDQKQLKAGDSFTIDWLPGTGTLVTVRGVPQPDPVKEPAFFNALLRIWLGPAPADWKLKDALLGK
>Vibrio.cholerae.ZP_04409871.1
MKKGLITLFVLLASGLGYAAVANAAQAEHKTFQRWAQWPVVGQATLSWLWLDIYSSQLRAPDGLYHESQDVSPHPVALEIRYLRDISSKQLVDATEDQWRKLGFTTPQTQAWLKQLQQILPDVVTGDRLVYVSDGQRGEFFFSRQQQTERSVGRIDDEAFNDAFLSIWLSPQTEYLTLRNQLIGMNRP
>Vibrio.fischeri.YP_206716
MAKQINMLILAGLLITSSVQSAPLSGLESSGKSELTWYLISVYQAQLFTSSGQFTYGEYPQALEIEYYRNISKENLIKATKEQWQKQAINHPDLNSWLEQLENVFPDVKPEDKLVFTIDENGTNEFFLNNKPIGSIANSDFSQLFLDIWVSEKTTYPDLRLELIGAE
>Vibrio.shilonii.ZP_01866260
MKFPRLITLLAAVVSVTAEAKIVSGVDVPDTLAIQQTELQLNGAGVRSKFFMDLYVGSLFTSLPSTEAASLIRGDQPSAIRLNITSSMITKEKLADALNEGFNNATDGNTTPIDSSINRFVEMTFANDISEGDQFTLVSAPEIGIYSYKNGEMLVLVEGDAFRRALLSVWLGDKPTDKSLKKAMLSQ
>Saccharomyces.Cerevisiae.NP_012068.1
MDRGRCANMLKSLQRTLAKCQKSPSTNHWQCFKRNFTSIRATKYPGRSNSTFHYWPWFAASTLLATSLYYRDRPVQNDDKTDAFPSHTESIQVDSSVSDFPLTITALNFPVSTTFKLLGYGQRHVTFLRFKVYALGLYLAENDENLVSDTLNETYLHKYFLDVDDSKTPKENLARLLKRDDSKSVMMIDDLLDSGMRMLAKITPVRNTDFKHLKEGLVKTISKHPDVANNKDTLAKGLSELNDAFSRKGSVRKNDDLIIELLANGALQFSYHDSKNNEFEVMGVVNNQLVGKFLFSQYLCGEKSPSPQAKKTAIDKLITLL
>Fusarium.graminearum.XP_382272.1
MLRPSTLRPALRASAHTRCAIVRTTRRSLFSQRSNRLTDDLDVNQLNSKRRDYEQNRTAFLAAGAIAGIVSFVYTAWKLKKAIEAQGEKEKAAIKCDTQVPAEIFKTEAGEKRKVVIHDEDGNEVVPTGNTTVKLFPRTLEVENVGSAGDVAGPIAAAVTDKHGTEFTLVGLGTRTVTFLGFEVYVVGFYVATQDVEKLQRYLVKKINPLATTLIPSEKEDLRKALQDATEGEETWNMILKDAGCRSAFRIIPVRDTDFPHMRDGLVRAVQARSARNPDYNDESFGEAMKHFKVLFQRGQVAKKNELLLVRDGAGKLTITYNDSTRKEPVKTVLGTVDDERLSRLLWLNYLAGNKVASEEARKNIINGVMEFVERPVGTVATQVL
>Lupinus.albus.LAGI01_19541
MGSLRFCFSLWNNNNNQPPSPHSSRSFSPFVVAVGFTVGAATALTALTCSNPSPLKSSDTNNSSIPTLPLWGSITMAQSSLPPTQSKNGISFPSSILSDSLNLLGIGFRRKSILGLKSIDIYAFGVYADNNDTKNYLAEKYGTLSASQLKGNKEFTEDLLENDISITVRLQIVYGKLSIRSVRNAFEESVGTRLKKFGESDNKELLQRFTSLFKDDIKIPSGSVIHLSREKGHVLRTTIDGEEMGSIQSKVLCKSILDLYIGEEPFDKKAKEDIELNLASHLQN
>Lupinus.albus.LAGI01_27083
MRNDWLSSMDSDPDILFPFDSFIFGLHYNNSRKFFVPGSLAIHEAFGRVTKLAGALLFWLSSSSSSNLVQDIASSMNNCHRFGNSTMGPVQVKPIVSNNVAGFGFPFRLKRKSSSSSVTLGKISSFIVRVIWREAKRIQSFPVLSLAAALIPPIQNLSSNVLAGPLQNPDVQMHGSIDQIHMEVESQGCPRLSVSELDMTKSAVETKTGIEFPVVLDNVLPGVHNSSFNSEVLVGTGSKNMTIVKIKSLKVYAFGFYVHPCSLCEKLGPKYASISVDELNSHHDFYQDLLREDINMTVRLVVNCKGMKINSVKVAFEKSLRARLVKTNPSTDFHCLTAFGSYFTEDIPLPLGTVIKFRRTVDGCLITEIGGNQIGSVHSKDL
>Lupinus.albus.LAGI01_48571
MFGTMAASTTLSLSPSINSSTFNFHHRIVFHKRISNSTLSFNHSHCFSLSSPSLMHFSSQNNNSTRHTHFFAAVASSSAANAEYVVEPITNVKFQKSLSLPGCADSLVLFGTGYREKVFAIIGVKVYAAGLYINQTIISELNAWKGQSKDMIQGNSSLFKTISQSSLEKSLQIILVRDVDGKTFWGALNDAVSPRIAEPTTIDETALRWSYFSLLESFGG
>Lupinus.albus.LAGI01_50470
VTPLKVDNVTFPPFVKATASNNNFFLGGAGVRGLQEHAKFIKFTDIGIYLQHNAISSLASKWHGKSPKKLTRSIHFFRDIITGPFEKFMQVTMILPLTGPQYSEKVAENCVAILKSLKVYTNEEQKATEKFLSVFKKETFAPGSSIFFTVLHQGSLAISFSKDACIPKVEAAIIKNKAFSEAVLESMIGENGVSPAAKKSLATRLSKLFKQGCGN
>Lupinus.albus.LAGI01_58790
ADEVVLVDEIPYPSKYTTTKPLSLLGYGITDMEIHFLQWKGKSAKELEENDDFFDALVSAPVEKVIRLVVIKEIKGAQYGVQIESAVRDRLAADDKYEEEEEEALEKIVEFLQSKYFKKHSVITYHFSADSQTAEIVVSLEGKDDSKFVVENANVVETIKKWYLGGSNAVSPSTISSLASTLSAELSK
>Lupinus.albus.LAGI01_59017
ADEVVLVDEIPYPSKYTTTKPLSLLVGHLHQWKGKSAKELEENDDFFDALVSAPVEKVIRLVVIKEIKGAQYGVQIESAVRDRLAADDKYEEEEEEALEKIVEFLQSKYFKKHSVITYHFSADSQTAEIVVSLEGKDDSKFVVENANV
>Lupinus.albus.LAGI01_34196
MADEVVLVDEFSYPSKYTTTKPLPLLGHGITDMVIHFLQVKFFSIGVYLDPEIVGHLHQWKGKSAKELEENDDFFDALVSAPVEKVIRLVVIKEIKGAQYGVQIESAVRDRLAADDKYEEEEEEALEKIVEFLQSKYFKKHSVITYHFSADSQTAEIVVSLEGK

DDSKFVVENANVVETIQKWYLGGSRAVSPSTIASLANTLSVELSK
>Lupinus.albus.LAGI01_60584
MADEVVLVDEFSYPSKYTTTKPLPLLGHGITDMVIHFLQVKFFSIGVYLDPEIVGHLHQWKGKSAKELEENDDFFDALVSA<br>PVEKVIRLVVIKEIKGAQYGVQIESAVRDRLAADDKYEEEEEEALEKIVEFLQSKYFKKHSVITYHFSADSQTAEIVVSLEGK<br>DDSKFVVENANVVETIQKWYLGGSRAVSPSTIASLDNSTES
>Lupinus.albus.LAGI01_63477
MVIHFLQVKFFSIGVYLDPEIVGHLHQWKGKSAKELEENDDFFDALVSAPVEKGIRLVGIKEIKGAQYGVQIESASKYFKKH<br>SVITYHFSADSQTAEIVVSLEGKDDSKFVVENANVVETIQKWYLGGSRAVSPSTIASLASTLSAELSK
>Lupinus.albus.LAGI01_73293
GHLQQWKGKSAKELEENDGFFDALIFAPVEKVVRLVVIKEIKASQYGVQIESAVRDRLAADDKYEEEEEEALEKIVEFLQS<br>KYFKKHSVITYHFSADSQTAEIVVSLEGKDDSKFVVENANV
>Lupinus.albus.LAGI01_35967
MTTPTVTSVEIEKVVFPSTVKPPGSDKAFFLGGAGVRGLQIQDNFVKFTAIGIYLQHDAVSSLAVNWNGKTAHELTESVQF<br>FRDIVTGPFDKFMQVTMLLPLTGHQYSEKVSENCVAIWKSLGIYTDEEEKAIHKFVSVFKDQTFPPGSSILFTVLPQGSLVIS<br>FSKDASIPEVEIAIIENKLLSQAVLESMIGRHGVSPAAKQSLATRLSELFKEGGAN
>Lupinus.luteus.l0itg32670
LVPPIQNLSSNVLAGPLQNPDVQMHGSIDQIHMEVDSQGCPRLSVSELNMTKSAVETKTGIEFPVVLDNVLPGVQNSSFNSE<br>VLVGTGSKNMTIVKIKSLKVYAFGFYIHPCSLCEKLGPKYASISANELNGHHDFYQDLLREDINMTVRLVVNCKGMKINSV<br>KVAFEKSLRARLVKTNPSTDFHCLTAFGSYFTEDIPLPLGTVIKFRRTVDGCLITEIGGNQIGSVHSKDLCRAFFDMYIGDAP<br>VSEQTKEEIGRNVVNIIRSC
>Lupinus.luteus.l0itg46375
MATPTVTSVEIENVVFPSTVKPPGSANAFFLGGAGVRGLQIQDNFVKFTSIGIYLHHDAVSSLAVKWNGKTAHELTESVEFF<br>RDIVTGPFDKFMQVTMLLPLTGHQYSEKVSENCVAIWKSLGIYTDEEEKAIEKFVSVFKDETFPPGSSILFTVLSQGSLVISFS<br>KDASIPEVETAIIENKLLAQAVLESMIGRHGVSPAAKQSLATRLSELLKEGGAN
>Lupinus.luteus.l0itg21939
MGSLRFSFSFWNNNPKPPTPHSSRPFSPFAVALGITAGAATAFTLLISSKHDPLKPVPLWGSITMADNALPVTQSKNGSSFPS<br>SILTDSLNLLGIGFRRKSIFGLKSIDVYAFGVYADNNDVKNYLAEKYGALSGSQLKGNKEFIQDVLENDISITVRLQILYGKL<br>SIRSVRNAFEESVGTRLQKYGGSDNKELLQRFTSLFKDDIKIPSGSVIHLSRGKGHVLSISIDGQEVGSIESQLLCKSLLDLYIG<br>DEPFDKKAKEEIELNLASHLQN
>Lupinus.luteus.l0itg08410
MADEVVLVDEISYPSKYTTTKPLPLLGHGITDMEIHFLQVKFYSIGVYLDPEIVGHLQQWKGKSAKELEENDDFFDALISAP<br>AEKVVRLVVIKEIKGAQYGVQIESAVRDRLAADDKYEEEEEEALEKIVEFLQSKYFKKHSVITYHFSADSQTAEIVVSLEGK<br>DDSKFVVENANVVETIQKWYLGGSRAVSPSTIASLANTLSVELSK
>Lupinus.luteus.l0itg40676
MANEVVLVDEIPYPSKYTTTKPLSLLGHGITDMVIHFLQVKFYSIGVYLDSEIVGHLQQWKGKSAKELEENDGFFDSLIFAP<br>VEKVVRLVVIKEIKGAQYGVQIESAVRDRLAADDKYEEEEEEALEKIVEFLQSKYFKKHSVITYHFSADSTTAEIVVSLEGK<br>EDSKFVVENANVVETIKKWYLGGSRAVSPSTISSLASTLSAELSK
>Lupinus.luteus.l0itg49121
TTLCFSPSINSSTFNFHKKKISNSTLSFNNSHCFSLSSPSLMHFSMQSNSRRQPHFFAAAASSSAANAEYVVEPATNVKFPKSL<br>SLPGSADYLVLLGTGYREKVFAIIGVKVYAAGLYLNQTIISELNAWKGQSKDKIQGNSSLFKTIYQSSLEKSLQIILVRDVDG<br>KTFWGALNDAISPRIAEPT
>Lupinus.angustifolius.CCM80406.1.HE999615.1
MNLISVADEVVLVDEIPYPSKYTTTKPLSLLGHGITDMEIHFLQVKFYSIGVYLDPEIVGHLQQWKGKSAKELEENDGFFDS<br>VIFAPVEKVVRLVVIKEIKGAQYGVQIESAVRDRLAADDKYEEEEEEALEKIVEFLQSKYFKKHSVITYHFSADSTTAEIVVS<br>LEGKEDSKFVVENANVVETIKKWYLGGSRAVSPSTISSLASTLSTELSK
>Lupinus.angustifolius.CCM80407.1.HE999614.1
MADEVVLVDEISYPSKYTTTKPLPLLGHGITDMEIHFLQVKFYSIGVYLDPEIVGHLQQWKGKSAKELEENDEFFDALVSAP<br>VEKVVRLVVIKEIKGAQYGVQIESAVRDRLAADDKYEEEEEEALEKIVEFLQSKYFKKHSVITYHFSADSQTAEIVVSLEGK<br>DDSKFVVVNANVVETIQKWYLGGSRAVSPSTIASLANTLSVELSK
>Lupinus.angustifolius.GBRP01042887.1
MRNDWLSSMDSDPDILFPFDSFIFGLHYHNSRKFFVPGSLAIQEAFGRVTKFAGALLFWLSSSSSSNLVQDIASSMNNYHRF<br>GNSTMGPVQVKPIVSNNVARFGFPFRLKRKSSSSSVTLGKISSFILRVIWREAKRIQSFPVLSLAAALVPPIQNLSSNILAGPLQ<br>NPDVQMNGSIDQIHMDVESQGCPRLSVSELNMTKSAVETKTGIEFPVVLDNVLPGVQNSSFNSEVLVGTGSKNMTIVKIKS

LKVYAFGFYVHPCSLCEKLGPKYASISVDELNSHHDFYQDLLREDINMTVRLVVNCKGMKINSVKVAFEKSLRARLVKTN<br>PSTDFHCLTAFGSYFTEDIPLPLGTVIKFRRTVDGCLITEIGGNQIGSVHSKDLCRAFFDMYIGDAPVSEQTKEEIGRNVANIIR<br>SC
>Lupinus.angustifolius.GBRP01065503.1
MFGTMAASTTLCLSPSINSSTFNFHHKKISNSTLSFTNSHCFSLSAPSLMHFSFQSSSRRQTHFFAAAASSSAASAECVVEPAT<br>NVKFQKSLSLPGNADSLVLFGTGYREKVFAIIGVKVYAAGLYLNQSIISELNAWKGQSKDKIQGNSSLFKTIYQSSLEKSLQI<br>ILVRDVDGKTFWGALNDAISPRIAKPTT
>Lupinus.angustifolius.KB407221.1
MNNYWLSFMDSDSDILFPFNSFIFGSNLLHYNDSSRNFYVPGSLSIPQAFERVTTLAGALLLWFSSCSSSNLVQDIAGSMNH<br>GSQFGTATMGSVKVKPNVAGFGFPFRLKRKSSSRALSLGKISSFAMRLIWREAKKFQSFHVLSLAAALVPPIQNLSSNLLAG<br>PLQNPDVQMHGSIDQIPREVESQGCARLSIHELNMTKPAVEPKTGIEFPVVLENVSPGNQSSSFNSEVLVGTGSRTMTIVKIK<br>SLNLYAFGFYVHPYSLCEKLGPKYASISADELNSHHGFYHDLLREDINMTVKFVVNCKGMKINSVKDAFEKSLRARLVKT<br>NPFTDFHCLTAFGSYFSEDIPLPLGTVIKFRRTVDGDLITEIGGNQIGSVHSKDLCRAFFDMYIGDVPVSEQTKEEIGRNVANI<br>IRKC
>Lupinus.angustifolius.KB432257.1
MAAPTVTSVEIENVVFPSTVKPPGSANAFFLGGAGVRGLQIQDNFVKFTAIGIYLQHHAVSSLAVKWNGKNAHELTESVEF<br>FRDIVTGPFDKFMQVTMLLPLTGQQYSEKVSENCVAIWKSLGIYTDEEEKAIEKFVSVFKDETFPPGSSILFTVLPQGSLVISF<br>SKDASIPEVETAIIENKLLSQAVLESMIGRHGVSPAAKQNLATRLSELLKEGGAN
>Lupinus.angustifolius.KB436138.1
MGSVRFSFSFWNNNPKPPSPHSSRPFSPFAVAVGLTAGAATAFTVLTSSNPNPLKPIPLFGSITMADNSLPATQSNNGSSFPSS<br>ILTDSLNLLGIGFRRKSIFGLKTIDIYAFGVYADNNDIKNYLAEKYGAVSASELKGNKELTEDLLENDISMTIRLQIVYGKLSI<br>RSVRNAFEESVGTRLKKYGGSDNKELLQRFTSMFKDDIKIPSGSVIHLSREKGHVLRTSIDGQEVGSIQSKVLCKSILDLYVG<br>EDPFDKKAKEDIVLNLASHLQN
>Lupinus.angustifolius.KB438712.1
MELVIRSPVKVENVTFPPFVNATGSNNNFFLGGAGVRGLQEQGKFIKFTDIGIYLQDNAVSSLADKWHGKSTKKLNKSNEF<br>FKDIIKGPFEKFMQVTLILPLSGPQYSEKVAENCAAILKSHGVYTNEEEKATEKFLSVFKKETFAPGSSIFFTVLHQGSLVISF<br>SRDAYIPKVEAAIIKNKALSEAVLESMIGENGVSPAAKKSLATRLSKLFKEGCAN

Chu, S., Wang, J., Cheng, H., Yang, Q., and Yu, D. (2014). Evolutionary study of the isoflavonoid pathway based on multiple copies analysis in soybean. *BMC Genet.* 15, 76. doi:10.1186/1471-2156-15-76.

Dastmalchi, M., and Dhaubhadel, S. (2015). Soybean chalcone isomerase: evolution of the fold, and the differential expression and localization of the gene family. *Planta* 241, 507–523. doi:10.1007/s00425-014-2200-5.

Liu, Y., Zhao, S., Wang, J., Zhao, C., Guan, H., Hou, L., Li, C., Xia, H., and Wang, X. (2015). Molecular cloning, expression, and evolution analysis of type II CHI gene from peanut (*Arachis hypogaea* L.). *Dev. Genes Evol.* 225, 1–10. doi:10.1007/s00427-015-0489-0.

Ngaki, M. N., Louie, G. V., Philippe, R. N., Manning, G., Pojer, F., Bowman, M. E., Li, L., Larsen, E., Wurtele, E. S., and Noel, J. P. (2012). Evolution of the chalcone-isomerase fold from fatty-acid binding to stereospecific catalysis. *Nature* 485, 530–533. doi:10.1038/nature11009.

Supplementary Table 3. List of sequences identified in the *Lupinus angustifolius* genome and transcriptome by BLAST analysis of 172 CHI-fold sequences.

% Identity	Bit-Score	E Value	*L. angustifolius* accession	CHI-fold gene accession
71.1%	342.043	1.99e-117	GBRP01001161.1	Phaseolus.vulgaris.Phvul.009G143100.1
65.4%	340.887	5.91e-117	GBRP01001161.1	CHI4B_Glycine.max

% Identity	Bit-Score	E Value	*L. angustifolius* accession	CHI-fold gene accession
57.7%	337.035	1.84e-115	GBRP01001161.1	CHI4A_Glycine.max
56.7%	313.923	1.80e-106	GBRP01001161.1	CHIL_Vitis.vinifera
70.9%	294.664	8.43e-99	GBRP01001161.1	CHIL_Aquilegia.formosa
80.5%	262.692	2.05e-86	GBRP01001161.1	CHIL_Ipomoea.nil
81.0%	259.996	2.85e-85	GBRP01001161.1	CHIL_Arabidopsis.thaliana
49.8%	241.121	7.15e-78	GBRP01001161.1	CHIL_Oryza.sativa
78.3%	230.72	1.48e-73	GBRP01001161.1	CHIL_Zea.mays
78.6%	156.762	1.91e-45	GBRP01001161.1	CHILb_Physcomitrella.patens
76.6%	145.591	3.52e-41	GBRP01001161.1	CHIL_Selaginella.moellendorffii
62.4%	144.821	7.71e-41	GBRP01001161.1	CHILa_Physcomitrella.patens
82.8%	130.954	1.26e-35	GBRP01001161.1	CHIL_Syntrichia.ruralis
84.9%	75.485	6.00e-47	KB421708.1	Phaseolus.vulgaris.Phvul.009G143100.1
78.6%	133.265	6.00e-47	KB421708.1	Phaseolus.vulgaris.Phvul.009G143100.1
76.5%	75.8702	6.78e-46	KB421708.1	CHI4B_Glycine.max
48.3%	129.413	6.78e-46	KB421708.1	CHI4B_Glycine.max
75.9%	77.0258	2.70e-45	KB421708.1	CHI4A_Glycine.max
75.9%	126.331	2.70e-45	KB421708.1	CHI4A_Glycine.max
76.5%	74.7146	2.65e-42	KB421708.1	CHIL_Vitis.vinifera
47.9%	118.627	2.65e-42	KB421708.1	CHIL_Vitis.vinifera
75.0%	65.855	8.94e-37	KB421708.1	CHIL_Aquilegia.formosa
60.0%	108.997	8.94e-37	KB421708.1	CHIL_Aquilegia.formosa
84.0%	62.003	6.91e-34	KB421708.1	CHIL_Ipomoea.nil
47.6%	102.834	6.91e-34	KB421708.1	CHIL_Ipomoea.nil
44.3%	58.151	9.40e-34	KB421708.1	CHIL_Oryza.sativa
56.6%	106.301	9.40e-34	KB421708.1	CHIL_Oryza.sativa
40.2%	58.5362	3.50e-33	KB421708.1	CHIL_Arabidopsis.thaliana
83.0%	103.99	3.50e-33	KB421708.1	CHIL_Arabidopsis.thaliana
52.1%	368.237	3.41e-126	GBRP01007050.1	Phaseolus.vulgaris.Phvul.002G276500.1
52.1%	360.533	8.86e-124	GBRP01007050.1	Medicago.truncatula.Medtr1g115870.1
51.6%	357.836	6.86e-123	GBRP01007050.1	CHI2_Lotus.japonicus
55.7%	352.058	1.06e-120	GBRP01007050.1	Phaseolus.vulgaris.Phvul.007G008500.1
51.4%	351.288	2.92e-120	GBRP01007050.1	Medicago.truncatula.Medtr1g115890.1
50.7%	348.206	1.63e-118	GBRP01007050.1	Arachis.hypogaea.JN660794
50.5%	332.413	8.44e-113	GBRP01007050.1	CHI2_Glycine.max
52.4%	327.791	5.86e-111	GBRP01007050.1	CHI3_Glycine.max
57.9%	319.316	1.52e-107	GBRP01007050.1	CHI_Vitis.vinifera
63.9%	301.597	1.57e-100	GBRP01007050.1	CHI_Ipomoea.batatas
64.4%	293.508	4.78e-97	GBRP01007050.1	CHI_Aquilegia.formosa
64.0%	289.271	1.31e-95	GBRP01007050.1	CHI_Arabidopsis.thaliana
62.8%	270.011	2.57e-88	GBRP01007050.1	CHI_Oryza.sativa
45.4%	268.085	1.27e-87	GBRP01007050.1	CHI_Zea.mays
62.4%	259.996	1.71e-84	GBRP01007050.1	CHI1b1_Glycine.max
61.5%	253.062	7.24e-82	GBRP01007050.1	Phaseolus.vulgaris.Phvul.003G216600.1
62.6%	251.521	3.37e-81	GBRP01007050.1	CHI1b2_Glycine.max

% Identity	Bit-Score	E Value	*L. angustifolius* accession	CHI-fold gene accession
63.4%	249.98	1.12e-80	GBRP01007050.1	Medicago.truncatula.Medtr1g115840.1
57.9%	237.654	5.29e-76	GBRP01007050.1	CHI1a_Glycine.max
63.0%	237.654	5.77e-76	GBRP01007050.1	Phaseolus.vulgaris.Phvul.007G008600.1
55.0%	236.498	1.73e-75	GBRP01007050.1	Medicago.truncatula.Medtr1g115820.1
63.5%	229.18	1.48e-72	GBRP01007050.1	Medicago.truncatula.Medtr1g115850.1
44.3%	228.794	2.39e-72	GBRP01007050.1	CHI1_Lotus.japonicus
55.0%	225.328	5.26e-71	GBRP01007050.1	Arachis.hypogaea.KP202691
55.3%	127.487	3.88e-34	GBRP01007050.1	CHI_Selaginella.moellendorffii
52.6%	117.472	6.06e-31	GBRP01007050.1	Medicago.truncatula.Medtr1g115830.1
34.2%	147.132	2.66e-38	KB432257.1	CHI2_Lotus.japonicus
70.2%	145.591	9.54e-38	KB432257.1	Phaseolus.vulgaris.Phvul.007G008500.1
48.1%	144.05	9.81e-37	KB432257.1	Phaseolus.vulgaris.Phvul.002G276500.1
30.0%	144.05	1.17e-36	KB432257.1	Phaseolus.vulgaris.Phvul.002G276500.1
29.2%	139.428	1.23e-35	KB432257.1	Medicago.truncatula.Medtr1g115870.1
25.7%	136.346	3.73e-34	KB432257.1	Arachis.hypogaea.JN660794
38.7%	134.42	7.14e-34	KB432257.1	Medicago.truncatula.Medtr1g115870.1
39.7%	134.035	8.29e-34	KB432257.1	CHI2_Lotus.japonicus
31.4%	134.035	9.95e-34	KB432257.1	CHI2_Glycine.max
64.7%	134.035	9.95e-34	KB432257.1	CHI3_Glycine.max
33.1%	133.65	1.21e-33	KB432257.1	Medicago.truncatula.Medtr1g115890.1
37.1%	133.265	2.42e-33	KB432257.1	CHI_Vitis.vinifera
69.1%	133.265	4.01e-33	KB432257.1	Arachis.hypogaea.JN660794
76.0%	131.724	5.06e-33	KB432257.1	Phaseolus.vulgaris.Phvul.007G008500.1
29.2%	130.568	1.55e-32	KB432257.1	Medicago.truncatula.Medtr1g115890.1
45.5%	128.257	1.40e-31	KB432257.1	CHI_Ipomoea.batatas
77.3%	124.02	2.80e-30	KB432257.1	CHI2_Glycine.max
28.1%	123.635	6.59e-30	KB432257.1	CHI_Arabidopsis.thaliana
65.2%	130.568	1.14e-37	GBRP01023746.1	Phaseolus.vulgaris.Phvul.009G143100.1
49.7%	126.716	2.81e-36	GBRP01023746.1	CHI4B_Glycine.max
47.8%	125.561	7.82e-36	GBRP01023746.1	CHI4A_Glycine.max
47.2%	119.398	1.98e-33	GBRP01023746.1	CHIL_Vitis.vinifera
86.3%	347.821	5.05e-119	GBRP01030694.1	Phaseolus.vulgaris.Phvul.001G152000.1
43.2%	338.191	2.03e-115	GBRP01030694.1	FAPa1_Glycine.max
85.0%	290.812	2.20e-96	GBRP01030694.1	FAPa1_Populus.trichocarpa
41.4%	286.189	6.15e-95	GBRP01030694.1	FAPa1_Oryza.sativa
53.1%	285.034	2.13e-94	GBRP01030694.1	FAPa1_Arabidopsis.thaliana
83.8%	279.641	2.74e-92	GBRP01030694.1	FAPa1_Zea.mays
36.8%	239.195	6.91e-77	GBRP01030694.1	FAPa1_Cycas.ovule
34.8%	223.402	2.99e-70	GBRP01030694.1	FAPa1_Physcomitrella.patens
36.0%	213.001	9.28e-67	GBRP01030694.1	FAPa1_Selaginella.moellendorffii
47.6%	152.91	6.93e-42	GBRP01030694.1	FAPa2b_Oryza.sativa
50.6%	142.124	6.22e-40	GBRP01030694.1	FAPa2_Selaginella.moellendorffii
42.4%	140.969	7.28e-40	GBRP01030694.1	FAPa2_Marchantia.polymorpha
42.7%	141.354	8.67e-39	GBRP01030694.1	FAPa2_Ginkgo.biloba
60.9%	143.665	1.86e-38	GBRP01030694.1	FAPa2a_Oryza.sativa

% Identity	Bit-Score	E Value	*L. angustifolius* accession	CHI-fold gene accession
35.8%	141.739	1.35e-37	GBRP01030694.1	FAPa2_Physcomitrella.patens
77.4%	135.191	1.29e-35	GBRP01030694.1	Phaseolus.vulgaris.Phvul.001G037700.1
39.6%	127.487	6.68e-33	GBRP01030694.1	Medicago.truncatula.Medtr1g015700.1
36.4%	123.635	1.56e-31	GBRP01030694.1	FAPa2_Arabidopsis.thaliana
59.0%	141.354	1.31e-35	KB436138.1	Phaseolus.vulgaris.Phvul.001G152000.1
28.8%	137.887	1.64e-34	KB436138.1	FAPa1_Glycine.max
40.9%	124.79	6.48e-30	KB436138.1	FAPa1_Zea.mays
32.0%	219.935	2.55e-70	GBRP01036913.1	Phaseolus.vulgaris.Phvul.002G276500.1
63.7%	215.698	2.45e-69	GBRP01036913.1	Phaseolus.vulgaris.Phvul.007G008500.1
80.5%	214.927	6.43e-69	GBRP01036913.1	CHI2_Lotus.japonicus
65.0%	211.075	2.20e-67	GBRP01036913.1	Medicago.truncatula.Medtr1g115870.1
80.5%	209.149	3.69e-66	GBRP01036913.1	Arachis.hypogaea.JN660794
48.1%	207.608	4.33e-66	GBRP01036913.1	Medicago.truncatula.Medtr1g115890.1
79.3%	201.83	8.73e-64	GBRP01036913.1	CHI2_Glycine.max
78.9%	198.749	1.98e-62	GBRP01036913.1	CHI_Vitis.vinifera
77.8%	197.208	5.56e-62	GBRP01036913.1	CHI3_Glycine.max
63.6%	191.045	2.66e-59	GBRP01036913.1	CHI_Arabidopsis.thaliana
63.3%	189.889	8.24e-59	GBRP01036913.1	CHI_Aquilegia.formosa
35.7%	181.03	1.86e-55	GBRP01036913.1	CHI_Ipomoea.batatas
46.0%	178.718	8.31e-55	GBRP01036913.1	CHI_Zea.mays
62.7%	171.4	6.48e-52	GBRP01036913.1	CHI_Oryza.sativa
39.3%	169.859	2.29e-51	GBRP01036913.1	CHI1_Lotus.japonicus
85.5%	169.088	3.66e-51	GBRP01036913.1	CHI1a_Glycine.max
70.5%	167.548	1.58e-50	GBRP01036913.1	CHI1b1_Glycine.max
75.6%	166.777	3.27e-50	GBRP01036913.1	Medicago.truncatula.Medtr1g115840.1
52.5%	162.925	7.51e-49	GBRP01036913.1	Phaseolus.vulgaris.Phvul.007G008600.1
63.4%	160.229	8.97e-48	GBRP01036913.1	Medicago.truncatula.Medtr1g115820.1
74.4%	160.229	9.55e-48	GBRP01036913.1	CHI1b2_Glycine.max
36.4%	159.073	2.58e-47	GBRP01036913.1	Phaseolus.vulgaris.Phvul.003G216600.1
36.8%	154.836	1.13e-45	GBRP01036913.1	Medicago.truncatula.Medtr1g115850.1
74.4%	150.214	6.82e-44	GBRP01036913.1	Arachis.hypogaea.KP202691
54.7%	176.022	3.75e-48	KB438712.1	CHI2_Lotus.japonicus
38.7%	174.481	1.68e-47	KB438712.1	Medicago.truncatula.Medtr1g115870.1
57.9%	173.326	2.82e-47	KB438712.1	Phaseolus.vulgaris.Phvul.007G008500.1
58.9%	172.94	2.16e-46	KB438712.1	Phaseolus.vulgaris.Phvul.002G276500.1
59.6%	170.244	4.04e-46	KB438712.1	Medicago.truncatula.Medtr1g115890.1
29.1%	168.703	1.56e-45	KB438712.1	CHI2_Glycine.max
39.3%	168.703	1.57e-45	KB438712.1	CHI3_Glycine.max
25.1%	163.696	1.77e-43	KB438712.1	Arachis.hypogaea.JN660794
36.7%	143.28	1.28e-36	KB438712.1	CHI_Ipomoea.batatas
59.6%	142.895	1.39e-36	KB438712.1	CHI_Vitis.vinifera
74.4%	139.813	2.45e-35	KB438712.1	CHI_Aquilegia.formosa
63.0%	139.043	3.95e-35	KB438712.1	CHI_Arabidopsis.thaliana
27.2%	134.035	1.59e-33	KB438712.1	CHI_Zea.mays

% Identity	Bit-Score	E Value	*L. angustifolius* accession	CHI-fold gene accession
60.7%	132.494	4.53e-33	KB438712.1	CHI_Oryza.sativa
61.4%	122.865	5.39e-30	KB438712.1	CHI1a_Glycine.max
49.5%	531.176	0	GBRP01042887.1	Phaseolus.vulgaris.Phvul.001G037700.1
40.9%	578.17	0	GBRP01042887.1	Medicago.truncatula.Medtr1g015700.1
45.7%	403.675	4.64e-136	GBRP01042887.1	FAPa2b_Populus.trichocarpa
63.7%	393.66	1.81e-132	GBRP01042887.1	FAPa2a_Populus.trichocarpa
69.5%	354.369	8.99e-117	GBRP01042887.1	FAPa2_Arabidopsis.thaliana
70.4%	324.709	8.08e-105	GBRP01042887.1	FAPa2b_Oryza.sativa
53.5%	276.944	2.00e-86	GBRP01042887.1	FAPa2a_Oryza.sativa
37.3%	239.58	4.40e-74	GBRP01042887.1	FAPa2_Ginkgo.biloba
60.0%	182.57	1.22e-50	GBRP01042887.1	FAPa2_Physcomitrella.patens
64.4%	174.481	2.19e-50	GBRP01042887.1	FAPa2_Selaginella.moellendorffii
66.7%	169.474	4.89e-49	GBRP01042887.1	FAPa2_Marchantia.polymorpha
52.4%	161.384	7.05e-45	GBRP01042887.1	FAPa1_Physcomitrella.patens
53.5%	143.665	4.81e-39	GBRP01042887.1	FAPa1_Cycas.ovule
51.6%	143.28	2.12e-38	GBRP01042887.1	FAPa1_Populus.trichocarpa
51.6%	139.813	1.58e-37	GBRP01042887.1	FAPa1_Glycine.max
65.9%	138.272	7.63e-37	GBRP01042887.1	Phaseolus.vulgaris.Phvul.001G152000.1
51.8%	134.42	1.72e-35	GBRP01042887.1	FAPa1_Oryza.sativa
58.3%	132.494	8.94e-35	GBRP01042887.1	FAPa1_Zea.mays
39.6%	121.709	5.53e-31	GBRP01042887.1	FAPa1_Arabidopsis.thaliana
52.6%	120.553	7.54e-31	GBRP01042887.1	FAPa1_Selaginella.moellendorffii
66.7%	259.61	8.48e-86	GBRP01065503.1	FAPb_Glycine.max
67.5%	219.164	7.07e-70	GBRP01065503.1	Phaseolus.vulgaris.Phvul.005G064500.1
56.0%	213.386	9.75e-68	GBRP01065503.1	Phaseolus.vulgaris.Phvul.005G064600.1
87.5%	178.333	1.74e-53	GBRP01065503.1	FAPb_Ipomoea.nil
55.9%	162.155	4.86e-48	GBRP01065503.1	FAPba_Populus.trichocarpa
87.5%	160.999	2.05e-47	GBRP01065503.1	FAPb_Arabidopsis.thaliana
71.8%	155.221	1.68e-45	GBRP01065503.1	FAPb_Aquilegia.formosa
63.0%	142.895	1.68e-40	GBRP01065503.1	FAPbb_Populus.trichocarpa
66.2%	120.939	1.07e-32	GBRP01065503.1	FAPba_Marchantia.polymorpha
66.2%	117.857	6.66e-32	GBRP01065503.1	FAPbb_Marchantia.polymorpha
51.2%	129.028	2.29e-30	KB407221.1	Medicago.truncatula.Medtr1g015700.1
23.5%	133.265	1.17e-33	KB430490.1	Phaseolus.vulgaris.Phvul.009G143100.1
56.0%	129.028	3.23e-32	KB430490.1	CHI4B_Glycine.max
48.8%	127.102	1.47e-31	KB430490.1	CHI4A_Glycine.max

Supplementary Table 4. Settings applied to construct MAFFT alignment.

Algorithm	E-INS-i
Scoring matrix	BLOSUM62
Gap open penalty	1.53
Offset value	0

Supplementary Table 5. Settings applied to construct MUSCLE re-alignment.

Maximum number of iterations	1000
Optimization	Anchor
Distance measure	iteration 1 kmer6_6, subsequent pctid_kimura
Clustering method	iteration 1&2 UPGMB, subsequent UPGMB
Tree rooting method	iteration 1&2 pseudo, subsequent pseudo
Subsequence weighting scheme	iteration 1&2 CLUSTALW, subsequent CLUSTALW
Terminal gaps	half penalty
Objective score	Spm
Anchor spacing	32
Gap open score	-1
Diagonals	minimum length 32, margin 5
Minimum column anchor scores	minimum best 2, minimum smoothed 1
Hydrophobicity	multiplier 1.2, window size 5

Supplementary Table 6. Settings applied to perform MrBayes inference of phylogeny.

Rate matrix	jones (JTT)
Rate variation	Gamma
Outgroup sequence	Arabidopsis thaliana FAPa1
Gamma categories	4
Chain length	1000000
Subsampling frequency	500
Burn-in length	100000
Nchains	4
Heated chain temperature	0.2
Unconstrained branch lengths	exponential 10
Shape parameter	exponential 10

Supplementary Table 7. Results of *L. albus* CHIL sequence (CA410672) alignment to NCBI Reference RNA database.

Accession	Sequence name	% identity	evalue	Total score	Query coverage
XM_004502772.1	PREDICTED: Cicer arietinum chalcone--flavonone isomerase-like (LOC101508569), mRNA	87.07	2E-148	533	96%
NM_001249853.1	Glycine max chalcone isomerase 4-like (LOC732547), mRNA	86.14	7E-143	515	96%
XM_006577815.1	PREDICTED: Glycine max chalcone isomerase 4B (LOC778027), transcript variant X1, mRNA	85.91	8E-142	511	96%
NM_001255112.1	Glycine max chalcone isomerase 4B (LOC778027), mRNA	85.91	8E-142	511	96%
XM_007137575.1	Phaseolus vulgaris hypothetical protein (PHAVU_009G143100g) mRNA, complete cds	85.68	3E-140	506	96%
XM_002280122.3	PREDICTED: Vitis vinifera probable chalcone--flavonone isomerase 3 (LOC100255217), mRNA	79.32	5E-88	333	85%
XM_007011250.1	Theobroma cacao Chalcone-flavanone isomerase family protein (TCM_045520) mRNA, complete cds	77.34	1E-83	318	90%

Accession	Sequence name	% identity	evalue	Total score	Query coverage
XM_008371146.1	PREDICTED: Malus x domestica probable chalcone--flavonone isomerase 3 (LOC103432919), transcript variant X2, mRNA	76.96	6E-80	306	91%
XM_008371145.1	PREDICTED: Malus x domestica probable chalcone--flavonone isomerase 3 (LOC103432919), transcript variant X1, mRNA	76.96	6E-80	306	91%
XM_002520824.1	Ricinus communis Chalcone--flavonone isomerase, putative, mRNA	78.34	2E-79	304	82%
XM_008235660.1	PREDICTED: Prunus mume probable chalcone--flavonone isomerase 3 (LOC103332893), mRNA	77.95	8E-79	302	85%
XM_011003097.1	PREDICTED: Populus euphratica probable chalcone--flavonone isomerase 3 (LOC105108689), mRNA	77.84	9E-78	298	82%
XM_007218345.1	Prunus persica hypothetical protein (PRUPE_ppa011476mg) mRNA, complete cds	77.37	9E-78	298	85%
XM_008368581.1	PREDICTED: Malus x domestica probable chalcone--flavonone isomerase 3 (LOC103430446), mRNA	77.53	3E-77	297	79%
XM_009350801.1	PREDICTED: Pyrus x bretschneideri probable chalcone--flavonone isomerase 3 (LOC103940646), transcript variant X2, mRNA	76.90	1E-75	291	85%
XM_009350800.1	PREDICTED: Pyrus x bretschneideri probable chalcone--flavonone isomerase 3 (LOC103940646), transcript variant X1, mRNA	76.90	1E-75	291	85%
XM_009365522.1	PREDICTED: Pyrus x bretschneideri probable chalcone--flavonone isomerase 3 (LOC103953741), mRNA	76.23	1E-75	291	91%
XM_002325890.1	Populus trichocarpa hypothetical protein (POPTR_0019s08610g) mRNA, complete cds	77.30	5E-75	289	82%
XM_004171007.1	PREDICTED: Cucumis sativus chalcone--flavonone isomerase-like (LOC101225344), mRNA	76.92	2E-74	288	84%
XM_004146426.1	PREDICTED: Cucumis sativus chalcone--flavonone isomerase-like (LOC101218716), mRNA	76.92	2E-74	288	84%
XM_010105346.1	Morus notabilis Chalcone--flavonone isomerase partial mRNA	74.58	7E-73	282	94%
XM_006371225.1	Populus trichocarpa hypothetical protein (POPTR_0019s08610g) mRNA, complete cds	78.30	7E-73	282	76%
XM_004307686.1	PREDICTED: Fragaria vesca subsp. vesca chalcone--flavonone isomerase-like (LOC101305307), mRNA	75.74	7E-73	282	91%
XM_008458730.1	PREDICTED: Cucumis melo probable chalcone--flavonone isomerase 3 (LOC103496746), mRNA	77.07	9E-72	279	80%
XM_009806094.1	PREDICTED: Nicotiana sylvestris probable chalcone--flavonone isomerase 3 (LOC104249627), mRNA	76.44	1E-69	271	81%
XM_006486106.1	PREDICTED: Citrus sinensis probable chalcone--flavonone isomerase 3-like (LOC102613490), mRNA	74.45	6E-68	266	91%
XM_006435856.1	Citrus clementina hypothetical protein (CICLE_v10032749mg) mRNA, complete cds	74.45	6E-68	266	91%
XM_006435855.1	Citrus clementina hypothetical protein (CICLE_v10032749mg) mRNA, complete cds	74.45	6E-68	266	91%
XM_006435854.1	Citrus clementina hypothetical protein (CICLE_v10032749mg) mRNA, complete cds	74.45	6E-68	266	91%
XM_010323153.1	PREDICTED: Solanum lycopersicum probable chalcone--flavonone isomerase 3 (LOC101266223), mRNA	76.03	7E-67	262	81%
XM_006365267.1	PREDICTED: Solanum tuberosum probable chalcone--flavonone isomerase 3-like (LOC102583606), mRNA	76.03	7E-67	262	81%
XM_009628946.1	PREDICTED: Nicotiana tomentosiformis probable chalcone--flavonone isomerase 3 (LOC104117823), mRNA	75.62	3E-65	257	81%
XM_010069312.1	PREDICTED: Eucalyptus grandis probable chalcone--flavonone isomerase 3 (LOC104454463), mRNA	74.07	2E-60	241	84%

Supplementary Table 8. Segregation data of molecular markers tagging *L. angustifolius* CHIL genes.

Line / Marker	97L380-008	97L380-009	97L380-010	97L380-011	97L380-012	97L380-013	97L380-014	97L380-015	97L380-016	97L380-017	97L380-018	97L380-019	97L380-021	97L380-023	97L380-024	97L380-025	97L380-026	97L380-027	97L380-028	97L380-032	97L380-033	97L380-034	97L380-035	97L380-036	97L380-037	97L380-038	97L380-039	97L380-040	97L380-041
CHIL1	2	2	1	0	2	1	2	1	1	1	2	1	2	2	1	2	2	2	1	0	0	1	1	1	0	2	1	2	2
CHIL2	1	2	1	0	1	1	1	2	2	H	2	1	2	2	1	2	1	1	1	0	0	2	2	1	0	2	1	1	1

Line / Marker	97L380-042	97L380-043	97L380-044	97L380-045	97L380-046	97L380-047	97L380-048	97L380-049	97L380-050	97L380-051	97L380-052	97L380-053	97L380-054	97L380-055	97L380-056	97L380-057	97L380-058	97L380-059	97L380-061	97L380-062	97L380-063	97L380-064	97L380-065	97L380-066	97L380-067	97L380-069	97L380-070	97L380-071	97L380-073
CHIL1	1	2	2	1	2	0	2	1	2	1	2	2	1	0	0	1	2	0	0	0	0	2	2	2	0	2	1	0	2
CHIL2	1	2	1	1	1	0	1	2	1	1	2	2	2	0	0	2	1	1	0	0	0	2	2	1	0	2	2	0	2

Line / Marker	97L380-074	97L380-075	97L380-076	97L380-077	97L380-078	97L380-080	97L380-081	97L380-082	97L380-083	97L380-084	97L380-085	97L380-086	97L380-089	97L380-090	97L380-092	97L380-093	97L380-094	97L380-095	97L380-096	97L380-097	97L380-098	97L380-099	97L380-100	97L380-101	97L380-102	97L380-104	97L380-105	97L380-106	97L380-107
CHIL1	1	0	2	1	1	1	0	1	1	1	0	2	1	2	2	2	2	1	0	2	2	1	1	0	2	2	0	1	2
CHIL2	1	0	1	2	2	1	0	2	1	1	2	1	1	2	2	1	1	1	0	0	1	1	1	0	2	1	0	2	1

Line / Marker	97L380-108	97L380-109	97L380-110	97L380-111	97L380-112	97L380-113	97L380-114	97L380-115	97L380-116	97L380-117	97L380-118	97L380-119	97L380-120	97L380-121	97L380-122	97L380-123	97L380-124	97L380-125	97L380-126	97L380-127	97L380-128	97L380-130	97L380-132	97L380-135	97L380-136	83A:476	P27255
CHIL1	0	2	0	2	1	1	1	0	2	0	2	1	2	0	1	1	1	1	0	1	2	1	2	1	2	2	1
CHIL2	0	1	0	2	1	1	1	0	1	0	2	1	1	0	1	2	1	1	0	2	2	1	1	2	1	2	1

1 - marker genotype the same as that of parental line P27255, 2 - marker genotype the same as that of parental line 83A:476, H - heterozygote

Recombinant inbred line numbering as described: Kroc M, Koczyk G, Święcicki W, Kilian A, Nelson MN. 2014. New evidence of ancestral polyploidy in the Genistoid legume *Lupinus angustifolius* L. (narrow-leafed lupin). Theoretical and Applied Genetics 127, 1237-1249.

Supplementary Table 9. Bacterial artificial chromosome-fluorescence in situ hybridization (BAC-FISH) on *L. angustifolius* metaphase chromosomes.

BAC clone	CHIL contig No.	Signal type
5L11	2	single locus
28O01	1	single locus
41I07	-	dispersed
88J04	1	single locus
106M03	1	dispersed
115N04	1	single locus
115L05	1	single locus
129C12	1	dispersed

134F01	2	single locus

Supplementary Table 10. List of anchored scaffolds and contigs with alignment data.

BES	Contig	Scaffold	Accession	Score	E-value	Identities	Gaps	Strand	Scaffold length
088J04_5	1	scaffold13140	KB407220.1	1254	0.0	703/724(97%)	2/724(0%)	+/+	6965
028O01_3	1	scaffold14855	KB408324.1	1421	0.0	792/802(99%)	7/802(0%)	+/+	24381
005L11_3	2	scaffold18235	KB410468.1	1341	0.0	741/748(99%)	5/748(0%)	+/-	20270
134F01_5	2	scaffold18235	KB410468.1	1288	0.0	709/719(99%)	1/719(0%)	+/-	20270
088J04_3	1	scaffold20923	KB412128.1	1389	0.0	754/756(99%)	0/756(0%)	+/-	33655
115L05_3	1	scaffold20923	KB412128.1	1286	0.0	711/724(98%)	1/724(0%)	+/-	33655
129C12_3	1	scaffold20923	KB412128.1	1122	0.0	633/653(97%)	3/653(0%)	+/-	33655
115N04_5	1	scaffold24794	KB414367.1	1657	0.0	902/904(99%)	2/904(0%)	+/-	10024
028O01_5	1	scaffold39988	KB421708.1	529	7E-153	297/303(98%)	0/303(0%)	+/-	24913
106M03_5	1	scaffold39988	KB421708.1	1483	0.0	803/803(100%)	0/803(0%)	+/-	24913
115L05_5	1	scaffold4701	KB424549.1	545	4E-158	295/295(100%)	0/295(0%)	+/-	14686
005L11_5	2	scaffold48639	KB425167.1	1216	0.0	665/672(99%)	0/672(0%)	+/+	22799
129C12_5	1	scaffold52240	KB426408.1	1188	0.0	654/663(99%)	1/663(0%)	+/-	2870
106M03_3	1	scaffold62847	AOCW01123280.1	1683	0.0	924/930(99%)	2/930(0%)	+/-	2017
134F01_3	2	scaffold65565	KB430490.1	1319	0.0	717/718(99%)	1/718(0%)	+/+	18174
115N04_3	1	scaffold90693	AOCW01151194.1	1504	0.0	824/828(99%)	3/828(0%)	+/-	6330

Supplementary Table 11. List of repeats annotated in scaffolds with sequence coordinates.

Contig	Scaffold	Accession	Scaffold sequence start	Scaffold sequence end	Strand	Repeat type
1	scaffold62847	AOCW01123280.1	38	86	+	Simple_repeat_(CATTATA)n
1	scaffold90693	AOCW01151194.1	113	161	+	Simple_repeat_(TTATT)n
1	scaffold90693	AOCW01151194.1	268	346	+	Simple_repeat_(ATTTAC)n
1	scaffold90693	AOCW01151194.1	706	747	+	Simple_repeat_(AAT)n
1	scaffold90693	AOCW01151194.1	1455	1488	+	Simple_repeat_(TTTATA)n
1	scaffold90693	AOCW01151194.1	3798	3843	+	Simple_repeat_(GTTAA)n
1	scaffold90693	AOCW01151194.1	5484	5520	+	Simple_repeat_(ACAAGA)n
1	scaffold13140	KB407220.1	1882	1920	+	Simple_repeat_(AATCA)n
1	scaffold13140	KB407220.1	5393	5425	+	Simple_repeat_(AATT)n
1	scaffold13140	KB407220.1	5491	5532	+	Simple_repeat_(TTTATA)n
1	scaffold14855	KB408324.1	1508	2107	-	LTR/Copia
1	scaffold14855	KB408324.1	2454	2912	-	LTR/Copia
1	scaffold14855	KB408324.1	7127	7753	+	Simple_repeat_(AT)n
1	scaffold14855	KB408324.1	8312	10639	+	LINE/L1
1	scaffold14855	KB408324.1	11950	12294	+	LINE/L1
1	scaffold14855	KB408324.1	12541	12593	+	Simple_repeat_(AT)n
1	scaffold14855	KB408324.1	19713	19787	+	Simple_repeat_(ATATTT)n
1	scaffold14855	KB408324.1	19960	20003	+	Simple_repeat_(ATTCA)n

Contig	Scaffold	Accession	Scaffold sequence start	Scaffold sequence end	Strand	Repeat type
1	scaffold14855	KB408324.1	21222	23470	-	LTR/Copia
1	scaffold20923	KB412128.1	193	240	+	Simple_repeat_(AT)n
1	scaffold20923	KB412128.1	1680	5147	+	LTR/Copia
1	scaffold20923	KB412128.1	6218	6273	+	Simple_repeat_(TGATAA)n
1	scaffold20923	KB412128.1	8131	11908	-	LTR/Copia
1	scaffold20923	KB412128.1	12834	12884	+	Simple_repeat_(TATT)n
1	scaffold20923	KB412128.1	13794	13931	+	Simple_repeat_(CTCGG)n
1	scaffold20923	KB412128.1	14003	14121	+	Simple_repeat_(TA)n
1	scaffold20923	KB412128.1	17752	17790	+	Simple_repeat_(TTCTT)n
1	scaffold20923	KB412128.1	18713	18766	+	Simple_repeat_(ATGAAT)n
1	scaffold20923	KB412128.1	19689	19713	+	Simple_repeat_(TAA)n
1	scaffold20923	KB412128.1	21339	21414	+	Simple_repeat_(AT)n
1	scaffold20923	KB412128.1	22409	22463	+	Simple_repeat_(AATT)n
1	scaffold20923	KB412128.1	28814	28900	+	Simple_repeat_(ATTAATA)n
1	scaffold20923	KB412128.1	29196	29245	+	Simple_repeat_(TAGT)n
1	scaffold20923	KB412128.1	29263	29319	+	Simple_repeat_(AATATTC)n
1	scaffold20923	KB412128.1	30873	30912	+	Simple_repeat_(TTAG)n
1	scaffold20923	KB412128.1	32572	32638	+	Simple_repeat_(AATTAA)n
1	scaffold20923	KB412128.1	33582	33618	+	Simple_repeat_(TAATATT)n
1	scaffold24794	KB414367.1	987	1015	+	Simple_repeat_(ATTC)n
1	scaffold24794	KB414367.1	1157	2149	+	LTR/Gypsy
1	scaffold24794	KB414367.1	2893	6109	+	LTR/Gypsy
1	scaffold24794	KB414367.1	6438	9588	+	LTR/Gypsy
1	scaffold39988	KB421708.1	2	55	+	Simple_repeat_(AATTA)n
1	scaffold39988	KB421708.1	317	363	+	Simple_repeat_(TAT)n
1	scaffold39988	KB421708.1	1434	1522	+	Simple_repeat_(AATATAT)n
1	scaffold39988	KB421708.1	3270	3311	+	Simple_repeat_(ATTGTGTTT)n
1	scaffold39988	KB421708.1	3659	3747	+	Simple_repeat_(AATTTAT)n
1	scaffold39988	KB421708.1	7712	7762	-	LTR/Copia
1	scaffold39988	KB421708.1	7974	9177	-	LTR/Copia
1	scaffold39988	KB421708.1	10318	11645	-	LTR/Copia
1	scaffold39988	KB421708.1	19568	19586	+	Simple_repeat_(AG)n
1	scaffold39988	KB421708.1	21441	21471	+	Simple_repeat_(TAATCA)n
1	scaffold39988	KB421708.1	23904	23926	+	Simple_repeat_(AAAT)n
1	scaffold4701	KB424549.1	1348	2258	+	LTR/Gypsy
1	scaffold4701	KB424549.1	2302	2418	+	LTR/Gypsy
1	scaffold4701	KB424549.1	8960	12922	+	LTR/Copia
2	scaffold65565	KB430490.1	1993	2054	+	Simple_repeat_(GAT)n
2	scaffold65565	KB430490.1	2608	2628	+	Simple_repeat_(AAGA)n
2	scaffold65565	KB430490.1	7979	8004	+	Simple_repeat_(TTTGA)n
2	scaffold65565	KB430490.1	8650	8679	+	Simple_repeat_(TTTTA)n
2	scaffold65565	KB430490.1	14921	14965	+	Simple_repeat_(CTACAG)n

Contig	Scaffold	Accession	Scaffold sequence start	Scaffold sequence end	Strand	Repeat type
2	scaffold65565	KB430490.1	14979	15137	-	LTR/Gypsy
2	scaffold65565	KB430490.1	16325	16421	+	Simple_repeat_(GTG)n
2	scaffold48639	KB425167.1	7824	7845	+	Simple_repeat_(TC)n
2	scaffold48639	KB425167.1	8037	8077	+	Simple_repeat_(ATAATT)n
2	scaffold48639	KB425167.1	10199	10251	+	Simple_repeat_(AAT)n
2	scaffold48639	KB425167.1	12975	13031	+	Simple_repeat_(TTAATT)n
2	scaffold48639	KB425167.1	14662	14716	+	Simple_repeat_(TAA)n
2	scaffold48639	KB425167.1	15189	15210	+	Simple_repeat_(ATAA)n
2	scaffold48639	KB425167.1	19890	19936	+	Simple_repeat_(CTTTACT)n
2	scaffold48639	KB425167.1	21325	22797	-	RC/Helitron
2	scaffold18235	KB410468.1	2964	3178	+	LTR/Gypsy
2	scaffold18235	KB410468.1	3232	3432	+	LTR/Gypsy
2	scaffold18235	KB410468.1	3518	4925	+	LTR/Gypsy
2	scaffold18235	KB410468.1	5229	5576	+	LTR/Gypsy
2	scaffold18235	KB410468.1	5666	8465	+	LTR/Gypsy
2	scaffold18235	KB410468.1	8493	8524	+	Simple_repeat_(AAAG)n
2	scaffold18235	KB410468.1	9160	9208	+	Simple_repeat_(TTTACAT)n
2	scaffold18235	KB410468.1	10261	10707	+	DNA/hAT-Ac
2	scaffold18235	KB410468.1	18388	19815	-	LINE/RTE-BovB

Supplementary Table 12. Summary of the scaffold repeat annotation.

Repeat type	CHIL contig 1	CHIL contig 2
Transpozon (total)	-[a]	3.1[b]
DNA/hAt	-	0.7
DNA/Helitron	-	2.4
Retrotranspozon (total)	22.4	10.7
LTR/Copia	13.6	-
LTR/Gypsy	6.7	8.4
non-LTR/LINE	2.1	2.3
Simple repeats	2.1	1.1
Repeats (total)	24.5	14.9

Supplementary Table 13. List of *Lupinus luteus* and *L. albus* transcriptome sequences aligned to scaffolds with alignment data.

Accession	Scaffold	Scaffold start	Scaffold end	Species	EST name	EST start	EST end	Percent identity	Alignment length	E-value	Score
KB412128.1	20923	16548	17385	*L. albus*	LAGI01_37613	703	1530	92.8	839	0	1229
KB412128.1	20923	16531	17508	*L. albus*	LAGI01_23966	966	1930	85.6	985	0	985

Accession	Scaffold	Scaffold start	Scaffold end	Species	EST name	EST start	EST end	Percent identity	Alignment length	E-value	Score
KB412128.1	20923	16548	17205	*L. albus*	LAGI01_44884	610	1251	90.1	658	0	852
KB412128.1	20923	16548	17205	*L. albus*	LAGI01_42053	688	1332	88.4	658	0	798
KB412128.1	20923	12897	13469	*L. albus*	LAGI01_36345	64	651	91.4	588	0	794
KB412128.1	20923	16548	17008	*L. albus*	LAGI01_36345	868	1325	94.5	461	0	733
KB412128.1	20923	25967	26643	*L. albus*	LAGI01_31136	671	9	86.6	683	0	712
KB412128.1	20923	25999	26629	*L. albus*	LAGI01_36995	886	1524	86.6	653	2.00E-180	639
KB412128.1	20923	13083	13469	*L. albus*	LAGI01_37613	91	486	93.6	396	3.00E-168	598
KB412128.1	20923	13086	13469	*L. albus*	LAGI01_44884	1	393	93.3	393	9.00E-165	587
KB412128.1	20923	25394	25759	*L. albus*	LAGI01_36995	523	888	93.7	366	4.00E-160	571
KB412128.1	20923	25414	25799	*L. albus*	LAGI01_31136	1215	826	91.8	391	3.00E-153	548
KB412128.1	20923	25445	25798	*L. albus*	LAGI01_66841	482	131	92.9	354	8.00E-147	527
KB412128.1	20923	25445	25759	*L. albus*	LAGI01_68685	227	541	94.9	315	9.00E-143	514
KB412128.1	20923	15712	16294	*L. albus*	LAGI01_56049	26	616	82.9	610	5.00E-126	458
KB412128.1	20923	26097	26357	*L. albus*	LAGI01_75015	519	259	96.9	261	2.00E-125	456
KB412128.1	20923	13086	13474	*L. albus*	LAGI01_61111	854	454	84.7	401	2.00E-111	410
KB412128.1	20923	30508	30864	*L. albus*	LAGI01_58927	415	62	85.5	360	7.00E-92	344
KB412128.1	20923	17842	18083	*L. albus*	LAGI01_36345	1321	1570	90.4	251	5.00E-86	325
KB412128.1	20923	16531	16753	*L. albus*	LAGI01_56049	771	993	91.4	223	2.00E-84	319
KB412128.1	20923	30510	30864	*L. albus*	LAGI01_25369	1364	1717	84.1	360	3.00E-80	306
KB412128.1	20923	30508	30792	*L. albus*	LAGI01_60296	286	7	86.7	286	4.00E-79	302
KB412128.1	20923	16531	16753	*L. albus*	LAGI01_49814	944	1166	90.1	223	4.00E-79	302
KB412128.1	20923	26390	26643	*L. albus*	LAGI01_75015	251	9	90.1	255	6.00E-78	298
KB412128.1	20923	24936	25137	*L. albus*	LAGI01_36995	50	250	92	202	2.00E-74	287
KB412128.1	20923	16548	16737	*L. albus*	LAGI01_61111	190	1	92.1	190	4.00E-72	279
KB412128.1	20923	24936	25137	*L. albus*	LAGI01_82299	41	241	91	202	5.00E-71	275
KB412128.1	20923	24936	25117	*L. albus*	LAGI01_31136	1697	1516	92.3	182	3.00E-69	269
KB412128.1	20923	25445	25764	*L. albus*	LAGI01_67726	194	513	81.2	320	3.00E-69	269
KB412128.1	20923	15800	16114	*L. albus*	LAGI01_86350	299	1	84.9	318	1.00E-68	267
KB412128.1	20923	24936	25114	*L. albus*	LAGI01_66841	659	481	92.1	179	2.00E-67	264
KB412128.1	20923	24936	25114	*L. albus*	LAGI01_68685	50	228	91.6	179	8.00E-66	258
KB412128.1	20923	25445	25758	*L. albus*	LAGI01_53379	876	563	80.8	314	8.00E-66	258
KB412128.1	20923	25445	25758	*L. albus*	LAGI01_45737	194	507	80.8	314	8.00E-66	258
KB412128.1	20923	14782	14916	*L. albus*	LAGI01_37613	484	618	99.2	135	1.00E-64	254
KB412128.1	20923	14782	14916	*L. albus*	LAGI01_44884	391	525	99.2	135	1.00E-64	254
KB412128.1	20923	14782	14916	*L. albus*	LAGI01_36345	649	783	99.2	135	1.00E-64	254
KB412128.1	20923	30568	30849	*L. albus*	LAGI01_52045	404	124	84.2	286	4.00E-64	252
KB412128.1	20923	16079	16294	*L. albus*	LAGI01_49814	571	789	87.6	219	2.00E-59	237
KB412128.1	20923	16079	16294	*L. albus*	LAGI01_23966	593	811	86.3	219	3.00E-54	219
KB412128.1	20923	17676	17831	*L. albus*	LAGI01_90903	160	3	91.7	158	3.00E-54	219
KB412128.1	20923	30504	30781	*L. albus*	LAGI01_40000	483	209	82.5	281	3.00E-54	219
KB412128.1	20923	26023	26288	*L. albus*	LAGI01_45737	530	795	80.8	266	1.00E-53	217
KB412128.1	20923	24945	25114	*L. albus*	LAGI01_91856	51	219	89.4	170	2.00E-52	214
KB412128.1	20923	30510	30764	*L. albus*	LAGI01_43384	787	1040	83.6	257	2.00E-52	214
KB412128.1	20923	13297	13474	*L. albus*	LAGI01_23966	302	476	88.2	178	6.00E-52	212
KB412128.1	20923	13297	13474	*L. albus*	LAGI01_42053	302	476	88.2	178	6.00E-52	212
KB412128.1	20923	26023	26303	*L. albus*	LAGI01_53379	540	260	79.7	281	6.00E-52	212
KB412128.1	20923	26020	26284	*L. albus*	LAGI01_13185	2015	1751	80.3	265	2.00E-51	210
KB412128.1	20923	25999	26124	*L. albus*	LAGI01_68685	539	664	94.4	126	5.00E-49	202
KB412128.1	20923	30661	30849	*L. albus*	LAGI01_94974	208	18	86.3	191	7.00E-48	198
KB412128.1	20923	26020	26284	*L. albus*	LAGI01_43592	529	793	79.6	265	7.00E-48	198
KB412128.1	20923	25451	25759	*L. albus*	LAGI01_42983	207	515	77.6	309	3.00E-47	196
KB412128.1	20923	26020	26262	*L. albus*	LAGI01_48300	360	602	80.2	243	1.00E-45	191
KB412128.1	20923	26020	26262	*L. albus*	LAGI01_43212	529	771	80.2	243	1.00E-45	191
KB412128.1	20923	26020	26262	*L. albus*	LAGI01_43189	529	771	80.2	243	1.00E-45	191
KB412128.1	20923	26020	26262	*L. albus*	LAGI01_41587	578	820	80.2	243	1.00E-45	191
KB412128.1	20923	26020	26262	*L. albus*	LAGI01_39823	632	874	80.2	243	1.00E-45	191
KB412128.1	20923	26020	26262	*L. albus*	LAGI01_13430	564	806	80.2	243	1.00E-45	191
KB412128.1	20923	26020	26262	*L. albus*	LAGI01_13430	1997	1755	80.2	243	1.00E-45	191

Accession	Scaffold	Scaffold start	Scaffold end	Species	EST name	EST start	EST end	Percent identity	Alignment length	E-value	Score
KB412128.1	20923	16033	16174	*L. albus*	LAGI01_61111	329	190	90.8	142	6.00E-45	189
KB412128.1	20923	11984	12354	*L. albus*	LAGI01_5057	1697	2057	78.1	371	6.00E-45	189
KB412128.1	20923	26020	26284	*L. albus*	LAGI01_47942	388	652	78.8	265	2.00E-44	187
KB412128.1	20923	26020	26284	*L. albus*	LAGI01_30699	929	1193	78.8	265	2.00E-44	187
KB412128.1	20923	30504	30693	*L. albus*	LAGI01_85199	313	127	85.8	191	3.00E-43	183
KB412128.1	20923	14782	14917	*L. albus*	LAGI01_61111	461	326	89.7	136	1.00E-42	181
KB412128.1	20923	14782	14916	*L. albus*	LAGI01_23966	469	603	89.6	135	4.00E-42	179
KB412128.1	20923	14782	14916	*L. albus*	LAGI01_42053	469	603	89.6	135	4.00E-42	179
KB412128.1	20923	26020	26262	*L. albus*	LAGI01_13185	592	834	79.4	243	4.00E-42	179
KB412128.1	20923	25451	25762	*L. albus*	LAGI01_30699	202	513	76.6	312	4.00E-42	179
KB412128.1	20923	25451	25762	*L. albus*	LAGI01_64802	33	344	76.6	312	4.00E-42	179
KB412128.1	20923	26019	26228	*L. albus*	LAGI01_45482	575	784	81.4	210	4.00E-42	179
KB412128.1	20923	26020	26262	*L. albus*	LAGI01_43230	529	771	79.4	243	4.00E-42	179
KB412128.1	20923	26020	26262	*L. albus*	LAGI01_41141	592	834	79.4	243	4.00E-42	179
KB412128.1	20923	25451	25759	*L. albus*	LAGI01_13185	265	573	76.3	309	2.00E-40	173
KB412128.1	20923	25451	25759	*L. albus*	LAGI01_43592	202	510	76.3	309	2.00E-40	173
KB412128.1	20923	25451	25759	*L. albus*	LAGI01_48300	33	341	76.3	309	2.00E-40	173
KB412128.1	20923	25451	25759	*L. albus*	LAGI01_43212	202	510	76.3	309	2.00E-40	173
KB412128.1	20923	25451	25759	*L. albus*	LAGI01_43189	202	510	76.3	309	2.00E-40	173
KB412128.1	20923	25451	25759	*L. albus*	LAGI01_41587	251	559	76.3	309	2.00E-40	173
KB412128.1	20923	25451	25759	*L. albus*	LAGI01_39823	305	613	76.3	309	2.00E-40	173
KB412128.1	20923	25451	25759	*L. albus*	LAGI01_13430	2324	2016	76.3	309	2.00E-40	173
KB412128.1	20923	25451	25759	*L. albus*	LAGI01_43230	202	510	76.3	309	2.00E-40	173
KB412128.1	20923	25451	25759	*L. albus*	LAGI01_41141	265	573	76.3	309	2.00E-40	173
KB412128.1	20923	7532	7830	*L. albus*	LAGI01_5057	1662	1950	79.2	299	3.00E-39	169
KB412128.1	20923	25451	25759	*L. albus*	LAGI01_13185	2342	2034	76	309	1.00E-38	167
KB412128.1	20923	25451	25759	*L. albus*	LAGI01_47942	61	369	76	309	1.00E-38	167
KB412128.1	20923	25451	25758	*L. albus*	LAGI01_47948	1013	700	76.4	314	1.00E-38	167
KB412128.1	20923	13285	13441	*L. albus*	LAGI01_49814	290	443	85.9	157	5.00E-38	166
KB412128.1	20923	25454	25759	*L. albus*	LAGI01_52195	261	572	76.2	312	2.00E-37	164
KB412128.1	20923	13297	13441	*L. albus*	LAGI01_73608	479	338	86.8	145	3.00E-36	160
KB412128.1	20923	30639	30764	*L. albus*	LAGI01_72909	1	127	88.9	127	1.00E-34	154
KB412128.1	20923	16079	16174	*L. albus*	LAGI01_37613	608	703	93.7	96	2.00E-33	150
KB412128.1	20923	16079	16174	*L. albus*	LAGI01_44884	515	610	93.7	96	2.00E-33	150
KB412128.1	20923	16079	16174	*L. albus*	LAGI01_36345	773	868	93.7	96	2.00E-33	150
KB412128.1	20923	14810	14916	*L. albus*	LAGI01_49814	475	581	90.6	107	8.00E-33	148
KB412128.1	20923	14810	14916	*L. albus*	LAGI01_73608	306	200	90.6	107	8.00E-33	148
KB412128.1	20923	16079	16176	*L. albus*	LAGI01_73608	210	113	91.8	98	4.00E-31	142
KB412128.1	20923	30504	30693	*L. albus*	LAGI01_56931	318	504	82.9	193	4.00E-31	142
KB412128.1	20923	25940	26066	*L. albus*	LAGI01_66841	125	3	86.6	127	2.00E-29	137
KB412128.1	20923	30733	30824	*L. albus*	LAGI01_99474	182	91	92.3	92	2.00E-29	137
KB412128.1	20923	25451	25759	*L. albus*	LAGI01_43678	1161	847	74.6	315	9.00E-29	135
KB412128.1	20923	15239	15374	*L. albus*	LAGI01_77874	453	323	84.5	136	1.00E-27	131
KB412128.1	20923	25451	25760	*L. albus*	LAGI01_66139	516	201	74.3	316	1.00E-27	131
KB412128.1	20923	25445	25759	*L. albus*	LAGI01_45413	1050	730	74.7	321	1.00E-27	131
KB412128.1	20923	25445	25759	*L. albus*	LAGI01_41132	256	576	74.7	321	1.00E-27	131
KB412128.1	20923	17422	17508	*L. albus*	LAGI01_90903	251	165	91.9	87	2.00E-26	127
KB412128.1	20923	25445	25689	*L. albus*	LAGI01_66163	215	465	76	251	2.00E-26	127
KB412128.1	20923	25445	25689	*L. albus*	LAGI01_49387	965	715	76	251	2.00E-26	127
KB412128.1	20923	25448	25626	*L. albus*	LAGI01_75200	46	224	78.7	179	7.00E-26	125
KB412128.1	20923	25454	25759	*L. albus*	LAGI01_48129	1018	707	74	312	3.00E-25	123
KB412128.1	20923	16079	16174	*L. albus*	LAGI01_42053	593	688	88.5	96	1.00E-24	121
KB412128.1	20923	29904	30031	*L. albus*	LAGI01_55920	679	805	84.3	128	1.00E-24	121
KB412128.1	20923	25795	25935	*L. albus*	LAGI01_2004	2958	3099	82.3	142	4.00E-24	119
KB412128.1	20923	25988	26228	*L. albus*	LAGI01_42983	502	742	75.1	241	1.00E-23	117
KB412128.1	20923	25988	26066	*L. albus*	LAGI01_74046	81	3	92.4	79	1.00E-23	117
KB412128.1	20923	25795	25931	*L. albus*	LAGI01_25253	141	4	82.6	138	1.00E-23	117
KB412128.1	20923	25795	25931	*L. albus*	LAGI01_12617	2510	2647	82.6	138	1.00E-23	117
KB412128.1	20923	25795	25931	*L. albus*	LAGI01_6441	3178	3315	82.6	138	1.00E-23	117

Accession	Scaffold	Scaffold start	Scaffold end	Species	EST name	EST start	EST end	Percent identity	Alignment length	E-value	Score
KB412128.1	20923	25445	25674	*L. albus*	LAGI01_79873	180	415	75.8	236	6.00E-23	116
KB412128.1	20923	15439	15608	*L. albus*	LAGI01_77874	230	64	80	170	8.00E-22	112
KB412128.1	20923	12148	12252	*L. albus*	LAGI01_64730	758	654	84.7	105	3.00E-21	110
KB412128.1	20923	29907	30009	*L. albus*	LAGI01_68072	264	365	86.4	103	1.00E-20	108
KB412128.1	20923	25795	25882	*L. albus*	LAGI01_61597	289	202	87.5	88	4.00E-20	106
KB412128.1	20923	29908	29995	*L. albus*	LAGI01_52791	415	328	87.5	88	4.00E-20	106
KB412128.1	20923	25445	25674	*L. albus*	LAGI01_47236	241	476	75.8	236	4.00E-20	106
KB412128.1	20923	25445	25674	*L. albus*	LAGI01_46813	1000	765	75.8	236	4.00E-20	106
KB412128.1	20923	29908	29995	*L. albus*	LAGI01_42123	724	637	87.5	88	4.00E-20	106
KB421708.1	39988	6284	6998	*L. albus*	LAGI01_40095	779	54	90	727	0	935
KB421708.1	39988	2981	3445	*L. albus*	LAGI01_60584	397	844	91.8	466	0	658
KB421708.1	39988	3314	3659	*L. albus*	LAGI01_34196	434	89	95.3	347	1.00E-155	556
KB421708.1	39988	15515	15977	*L. albus*	LAGI01_72279	567	117	86.8	472	2.00E-142	512
KB421708.1	39988	5621	5998	*L. albus*	LAGI01_40095	1449	1071	89.7	381	1.00E-129	469
KB421708.1	39988	2933	3208	*L. albus*	LAGI01_34196	708	433	94.9	276	8.00E-124	450
KB421708.1	39988	18454	18712	*L. albus*	LAGI01_11627	928	1186	96.1	259	6.00E-121	440
KB421708.1	39988	18454	18712	*L. albus*	LAGI01_11584	928	1186	96.1	259	6.00E-121	440
KB421708.1	39988	18454	18712	*L. albus*	LAGI01_11583	1803	1545	96.1	259	6.00E-121	440
KB421708.1	39988	18454	18712	*L. albus*	LAGI01_11540	1799	1541	96.1	259	6.00E-121	440
KB421708.1	39988	18454	18712	*L. albus*	LAGI01_11535	1799	1541	96.1	259	6.00E-121	440
KB421708.1	39988	18454	18712	*L. albus*	LAGI01_11534	1799	1541	96.1	259	6.00E-121	440
KB421708.1	39988	18454	18712	*L. albus*	LAGI01_11490	1803	1545	96.1	259	6.00E-121	440
KB421708.1	39988	5665	5998	*L. albus*	LAGI01_55176	1016	683	90.7	336	2.00E-120	439
KB421708.1	39988	2170	2442	*L. albus*	LAGI01_63477	1	275	94.5	275	1.00E-118	433
KB421708.1	39988	15521	15825	*L. albus*	LAGI01_11627	15	322	90.9	308	3.00E-112	412
KB421708.1	39988	15521	15825	*L. albus*	LAGI01_11584	15	322	90.9	308	3.00E-112	412
KB421708.1	39988	15521	15825	*L. albus*	LAGI01_11583	2716	2409	90.9	308	3.00E-112	412
KB421708.1	39988	18454	18712	*L. albus*	LAGI01_11536	936	1194	94.2	259	3.00E-112	412
KB421708.1	39988	2981	3208	*L. albus*	LAGI01_59017	300	73	96.9	228	3.00E-108	398
KB421708.1	39988	2981	3208	*L. albus*	LAGI01_58790	551	324	96.9	228	3.00E-108	398
KB421708.1	39988	3314	3607	*L. albus*	LAGI01_63477	500	790	90.8	294	7.00E-106	390
KB421708.1	39988	2981	3213	*L. albus*	LAGI01_60727	469	237	95.7	233	7.00E-106	390
KB421708.1	39988	2981	3208	*L. albus*	LAGI01_63477	275	501	95.6	228	4.00E-100	371
KB421708.1	39988	3314	3627	*L. albus*	LAGI01_58790	325	15	88.2	314	4.00E-100	371
KB421708.1	39988	2981	3208	*L. albus*	LAGI01_73293	260	487	94.2	228	9.00E-98	364
KB421708.1	39988	2042	2275	*L. albus*	LAGI01_71298	236	1	94	236	9.00E-98	364
KB421708.1	39988	2286	2548	*L. albus*	LAGI01_34196	1401	1129	89	273	2.00E-88	333
KB421708.1	39988	2218	2442	*L. albus*	LAGI01_59017	525	300	92.4	226	9.00E-87	327
KB421708.1	39988	6284	6621	*L. albus*	LAGI01_55176	397	60	82.8	338	3.00E-83	316
KB421708.1	39988	2218	2431	*L. albus*	LAGI01_73293	35	249	92	215	2.00E-80	306
KB421708.1	39988	20818	20994	*L. albus*	LAGI01_11627	1470	1646	96	177	1.00E-78	300
KB421708.1	39988	20818	20994	*L. albus*	LAGI01_11584	1470	1646	96	177	1.00E-78	300
KB421708.1	39988	20818	20994	*L. albus*	LAGI01_11583	1261	1085	96	177	1.00E-78	300
KB421708.1	39988	20818	20994	*L. albus*	LAGI01_11540	1257	1081	96	177	1.00E-78	300
KB421708.1	39988	20818	20994	*L. albus*	LAGI01_11535	1257	1081	96	177	1.00E-78	300
KB421708.1	39988	20818	20994	*L. albus*	LAGI01_11534	1257	1081	96	177	1.00E-78	300
KB421708.1	39988	20818	20994	*L. albus*	LAGI01_11490	1261	1085	96	177	1.00E-78	300
KB421708.1	39988	20818	20994	*L. albus*	LAGI01_11536	1478	1654	96	177	1.00E-78	300
KB421708.1	39988	16472	16672	*L. albus*	LAGI01_25560	381	181	92.5	201	1.00E-78	300
KB421708.1	39988	1817	2024	*L. albus*	LAGI01_81059	384	176	91.3	209	3.00E-75	289
KB421708.1	39988	19399	19564	*L. albus*	LAGI01_11627	1184	1349	95.7	166	3.00E-72	279
KB421708.1	39988	22149	22305	*L. albus*	LAGI01_11627	1879	2035	97.4	157	3.00E-72	279
KB421708.1	39988	19399	19564	*L. albus*	LAGI01_11584	1184	1349	95.7	166	3.00E-72	279
KB421708.1	39988	22149	22305	*L. albus*	LAGI01_11584	1879	2035	97.4	157	3.00E-72	279
KB421708.1	39988	22149	22305	*L. albus*	LAGI01_11583	852	696	97.4	157	3.00E-72	279
KB421708.1	39988	19399	19564	*L. albus*	LAGI01_11583	1547	1382	95.7	166	3.00E-72	279
KB421708.1	39988	22149	22305	*L. albus*	LAGI01_11540	848	692	97.4	157	3.00E-72	279

Accession	Scaffold	Scaffold start	Scaffold end	Species	EST name	EST start	EST end	Percent identity	Alignment length	E-value	Score
KB421708.1	39988	19399	19564	*L. albus*	LAGI01_11540	1543	1378	95.7	166	3.00E-72	279
KB421708.1	39988	22149	22305	*L. albus*	LAGI01_11535	848	692	97.4	157	3.00E-72	279
KB421708.1	39988	19399	19564	*L. albus*	LAGI01_11535	1543	1378	95.7	166	3.00E-72	279
KB421708.1	39988	22149	22305	*L. albus*	LAGI01_11534	848	692	97.4	157	3.00E-72	279
KB421708.1	39988	22149	22305	*L. albus*	LAGI01_11490	852	696	97.4	157	3.00E-72	279
KB421708.1	39988	19399	19564	*L. albus*	LAGI01_11490	1547	1382	95.7	166	3.00E-72	279
KB421708.1	39988	19399	19564	*L. albus*	LAGI01_11536	1192	1357	95.7	166	3.00E-72	279
KB421708.1	39988	22149	22305	*L. albus*	LAGI01_11536	1887	2043	97.4	157	3.00E-72	279
KB421708.1	39988	19399	19564	*L. albus*	LAGI01_11534	1543	1378	95.1	166	1.00E-70	273
KB421708.1	39988	2286	2442	*L. albus*	LAGI01_60584	241	397	95.5	157	4.00E-67	262
KB421708.1	39988	2286	2442	*L. albus*	LAGI01_60727	625	469	95.5	157	4.00E-67	262
KB421708.1	39988	2286	2442	*L. albus*	LAGI01_81059	177	21	95.5	157	4.00E-67	262
KB421708.1	39988	17430	17581	*L. albus*	LAGI01_11627	535	686	96	152	6.00E-66	258
KB421708.1	39988	17430	17581	*L. albus*	LAGI01_11584	535	686	96	152	6.00E-66	258
KB421708.1	39988	17430	17581	*L. albus*	LAGI01_11583	2196	2045	96	152	6.00E-66	258
KB421708.1	39988	2286	2442	*L. albus*	LAGI01_58790	708	551	95.5	158	9.00E-65	254
KB421708.1	39988	16532	16672	*L. albus*	LAGI01_11627	397	537	97.1	141	5.00E-63	248
KB421708.1	39988	16532	16672	*L. albus*	LAGI01_11584	397	537	97.1	141	5.00E-63	248
KB421708.1	39988	16532	16672	*L. albus*	LAGI01_11583	2334	2194	97.1	141	5.00E-63	248
KB421708.1	39988	23974	24201	*L. albus*	LAGI01_11584	2433	2654	86.4	229	2.00E-58	233
KB421708.1	39988	23974	24201	*L. albus*	LAGI01_11583	298	77	86.4	229	2.00E-58	233
KB421708.1	39988	23974	24201	*L. albus*	LAGI01_11490	298	77	86.4	229	2.00E-58	233
KB421708.1	39988	16532	16672	*L. albus*	LAGI01_11540	2330	2190	94.3	141	4.00E-56	225
KB421708.1	39988	16532	16672	*L. albus*	LAGI01_11535	2330	2190	94.3	141	4.00E-56	225
KB421708.1	39988	16532	16672	*L. albus*	LAGI01_11534	2330	2190	94.3	141	4.00E-56	225
KB421708.1	39988	16532	16672	*L. albus*	LAGI01_11490	2334	2194	94.3	141	4.00E-56	225
KB421708.1	39988	16532	16672	*L. albus*	LAGI01_11536	405	545	94.3	141	4.00E-56	225
KB421708.1	39988	16532	16672	*L. albus*	LAGI01_25560	1624	1764	94.3	141	4.00E-56	225
KB421708.1	39988	16532	16672	*L. albus*	LAGI01_47899	321	181	94.3	141	4.00E-56	225
KB421708.1	39988	16532	16672	*L. albus*	LAGI01_47899	946	1086	94.3	141	4.00E-56	225
KB421708.1	39988	19981	20105	*L. albus*	LAGI01_11627	1347	1471	97.6	125	2.00E-55	223
KB421708.1	39988	19981	20105	*L. albus*	LAGI01_11584	1347	1471	97.6	125	2.00E-55	223
KB421708.1	39988	19981	20105	*L. albus*	LAGI01_11583	1384	1260	97.6	125	2.00E-55	223
KB421708.1	39988	19981	20105	*L. albus*	LAGI01_11540	1380	1256	97.6	125	2.00E-55	223
KB421708.1	39988	17430	17581	*L. albus*	LAGI01_11540	2192	2041	92.1	152	2.00E-55	223
KB421708.1	39988	19981	20105	*L. albus*	LAGI01_11535	1380	1256	97.6	125	2.00E-55	223
KB421708.1	39988	17430	17581	*L. albus*	LAGI01_11535	2192	2041	92.1	152	2.00E-55	223
KB421708.1	39988	17430	17581	*L. albus*	LAGI01_11534	2192	2041	92.1	152	2.00E-55	223
KB421708.1	39988	19981	20105	*L. albus*	LAGI01_11490	1384	1260	97.6	125	2.00E-55	223
KB421708.1	39988	17430	17581	*L. albus*	LAGI01_11490	2196	2045	92.1	152	2.00E-55	223
KB421708.1	39988	17430	17581	*L. albus*	LAGI01_11536	543	694	92.1	152	2.00E-55	223
KB421708.1	39988	19981	20105	*L. albus*	LAGI01_11536	1355	1479	97.6	125	2.00E-55	223
KB421708.1	39988	17430	17581	*L. albus*	LAGI01_25560	183	32	91.4	152	9.00E-54	217
KB421708.1	39988	17430	17581	*L. albus*	LAGI01_25560	1762	1913	91.4	152	9.00E-54	217
KB421708.1	39988	17430	17581	*L. albus*	LAGI01_47899	183	32	91.4	152	9.00E-54	217
KB421708.1	39988	17722	17844	*L. albus*	LAGI01_11627	685	807	96.7	123	1.00E-52	214
KB421708.1	39988	17722	17844	*L. albus*	LAGI01_11584	685	807	96.7	123	1.00E-52	214
KB421708.1	39988	17722	17844	*L. albus*	LAGI01_11540	2042	1920	96.7	123	1.00E-52	214
KB421708.1	39988	17722	17844	*L. albus*	LAGI01_11535	2042	1920	96.7	123	1.00E-52	214
KB421708.1	39988	17722	17844	*L. albus*	LAGI01_11490	2046	1924	96.7	123	1.00E-52	214
KB421708.1	39988	17722	17844	*L. albus*	LAGI01_11536	693	815	96.7	123	1.00E-52	214
KB421708.1	39988	1653	1792	*L. albus*	LAGI01_60584	3	143	93.6	141	2.00E-51	210
KB421708.1	39988	1653	1792	*L. albus*	LAGI01_34196	1639	1499	93.6	141	2.00E-51	210
KB421708.1	39988	1653	1792	*L. albus*	LAGI01_60727	863	723	93.6	141	2.00E-51	210
KB421708.1	39988	1653	1792	*L. albus*	LAGI01_71298	602	462	93.6	141	2.00E-51	210
KB421708.1	39988	5799	5998	*L. albus*	LAGI01_69145	565	369	86.2	203	2.00E-51	210
KB421708.1	39988	5799	5998	*L. albus*	LAGI01_68593	161	357	86.2	203	2.00E-51	210
KB421708.1	39988	17722	17844	*L. albus*	LAGI01_11534	2042	1920	95.1	123	4.00E-49	202
KB421708.1	39988	17430	17569	*L. albus*	LAGI01_47899	1084	1223	91.4	140	1.00E-48	200

Accession	Scaffold	Scaffold start	Scaffold end	Species	EST name	EST start	EST end	Percent identity	Alignment length	E-value	Score
KB421708.1	39988	17722	17844	*L. albus*	LAGI01_11583	2046	1924	94.3	123	2.00E-47	196
KB421708.1	39988	17954	18076	*L. albus*	LAGI01_11627	806	928	92.6	123	6.00E-44	185
KB421708.1	39988	17954	18076	*L. albus*	LAGI01_11584	806	928	92.6	123	6.00E-44	185
KB421708.1	39988	17954	18076	*L. albus*	LAGI01_11583	1925	1803	92.6	123	6.00E-44	185
KB421708.1	39988	17954	18076	*L. albus*	LAGI01_11540	1921	1799	92.6	123	6.00E-44	185
KB421708.1	39988	17954	18076	*L. albus*	LAGI01_11535	1921	1799	92.6	123	6.00E-44	185
KB421708.1	39988	17954	18076	*L. albus*	LAGI01_11534	1921	1799	92.6	123	6.00E-44	185
KB421708.1	39988	17954	18076	*L. albus*	LAGI01_11490	1925	1803	92.6	123	6.00E-44	185
KB421708.1	39988	1809	2078	*L. albus*	LAGI01_59017	907	647	81.2	272	9.00E-43	181
KB421708.1	39988	20575	20796	*L. albus*	LAGI01_89427	47	270	82.1	224	9.00E-43	181
KB421708.1	39988	19981	20094	*L. albus*	LAGI01_11534	1380	1267	92.1	114	1.00E-38	167
KB421708.1	39988	1940	2033	*L. albus*	LAGI01_71298	447	354	96.8	94	1.00E-37	164
KB421708.1	39988	21670	21759	*L. albus*	LAGI01_11627	1643	1732	97.7	90	5.00E-37	162
KB421708.1	39988	22768	22851	*L. albus*	LAGI01_11627	2090	2173	100	84	5.00E-37	162
KB421708.1	39988	21670	21759	*L. albus*	LAGI01_11584	1643	1732	97.7	90	5.00E-37	162
KB421708.1	39988	22768	22851	*L. albus*	LAGI01_11584	2090	2173	100	84	5.00E-37	162
KB421708.1	39988	21670	21759	*L. albus*	LAGI01_11583	1088	999	97.7	90	5.00E-37	162
KB421708.1	39988	21670	21759	*L. albus*	LAGI01_11540	1084	995	97.7	90	5.00E-37	162
KB421708.1	39988	22768	22851	*L. albus*	LAGI01_11535	637	554	100	84	5.00E-37	162
KB421708.1	39988	22768	22851	*L. albus*	LAGI01_11534	637	554	100	84	5.00E-37	162
KB421708.1	39988	21670	21759	*L. albus*	LAGI01_11534	1084	995	97.7	90	5.00E-37	162
KB421708.1	39988	22768	22851	*L. albus*	LAGI01_11490	641	558	100	84	5.00E-37	162
KB421708.1	39988	21670	21759	*L. albus*	LAGI01_11490	1088	999	97.7	90	5.00E-37	162
KB421708.1	39988	21670	21759	*L. albus*	LAGI01_11536	1651	1740	97.7	90	5.00E-37	162
KB421708.1	39988	22768	22851	*L. albus*	LAGI01_11536	2098	2181	100	84	5.00E-37	162
KB421708.1	39988	1809	2024	*L. albus*	LAGI01_58790	912	707	82	217	1.00E-34	154
KB421708.1	39988	20725	20845	*L. albus*	LAGI01_107777	23	142	89.2	121	6.00E-33	148
KB421708.1	39988	1940	2024	*L. albus*	LAGI01_60584	158	242	96.4	85	2.00E-32	146
KB421708.1	39988	1940	2024	*L. albus*	LAGI01_34196	1484	1400	96.4	85	2.00E-32	146
KB421708.1	39988	1940	2024	*L. albus*	LAGI01_60727	708	624	96.4	85	2.00E-32	146
KB421708.1	39988	21732	21999	*L. albus*	LAGI01_88036	1	259	77.6	268	1.00E-30	141
KB421708.1	39988	22768	22848	*L. albus*	LAGI01_11583	641	561	96.2	81	5.00E-30	139
KB421708.1	39988	22768	22848	*L. albus*	LAGI01_11540	637	557	96.2	81	5.00E-30	139
KB421708.1	39988	17954	18076	*L. albus*	LAGI01_11536	814	936	86.1	123	5.00E-30	139
KB421708.1	39988	3788	3931	*L. albus*	LAGI01_27262	23	167	84.1	145	2.00E-29	137
KB421708.1	39988	3788	3931	*L. albus*	LAGI01_16562	23	167	84.1	145	2.00E-29	137
KB421708.1	39988	7352	7530	*L. albus*	LAGI01_37309	1333	1508	82.2	180	3.00E-28	133
KB421708.1	39988	7352	7530	*L. albus*	LAGI01_6414	1318	1142	82.2	180	3.00E-28	133
KB421708.1	39988	3314	3386	*L. albus*	LAGI01_59017	74	2	97.2	73	4.00E-27	129
KB421708.1	39988	3314	3386	*L. albus*	LAGI01_73293	486	558	97.2	73	4.00E-27	129
KB421708.1	39988	23795	23866	*L. albus*	LAGI01_11584	2360	2431	97.2	72	1.00E-26	127
KB421708.1	39988	23795	23866	*L. albus*	LAGI01_11583	371	300	97.2	72	1.00E-26	127
KB421708.1	39988	23795	23866	*L. albus*	LAGI01_11490	371	300	97.2	72	1.00E-26	127
KB421708.1	39988	2767	2885	*L. albus*	LAGI01_76267	322	440	84.8	119	5.00E-26	125
KB421708.1	39988	23124	23201	*L. albus*	LAGI01_11627	2172	2249	93.5	78	8.00E-25	121
KB421708.1	39988	23124	23201	*L. albus*	LAGI01_11584	2172	2249	93.5	78	8.00E-25	121
KB421708.1	39988	23124	23201	*L. albus*	LAGI01_11535	555	478	93.5	78	8.00E-25	121
KB421708.1	39988	21670	21759	*L. albus*	LAGI01_11535	1084	995	90	90	8.00E-25	121
KB421708.1	39988	23124	23201	*L. albus*	LAGI01_11534	555	478	93.5	78	8.00E-25	121
KB421708.1	39988	23124	23201	*L. albus*	LAGI01_11490	559	482	93.5	78	8.00E-25	121
KB421708.1	39988	23124	23201	*L. albus*	LAGI01_11536	2180	2257	93.5	78	8.00E-25	121
KB421708.1	39988	23459	23526	*L. albus*	LAGI01_11627	2294	2361	97	68	3.00E-24	119
KB421708.1	39988	23459	23526	*L. albus*	LAGI01_11584	2294	2361	97	68	3.00E-24	119
KB421708.1	39988	23459	23526	*L. albus*	LAGI01_11535	433	366	97	68	3.00E-24	119
KB421708.1	39988	23459	23526	*L. albus*	LAGI01_11534	433	366	97	68	3.00E-24	119
KB421708.1	39988	23459	23526	*L. albus*	LAGI01_11490	437	370	97	68	3.00E-24	119
KB421708.1	39988	23459	23526	*L. albus*	LAGI01_11536	2302	2369	97	68	3.00E-24	119
KB421708.1	39988	6284	6366	*L. albus*	LAGI01_69145	83	1	91.5	83	3.00E-24	119

Accession	Scaffold	Scaffold start	Scaffold end	Species	EST name	EST start	EST end	Percent identity	Alignment length	E-value	Score
KB421708.1	39988	4269	4455	*L. albus*	LAGI01_98567	2	188	79.2	188	1.00E-23	117
KB421708.1	39988	23974	24055	*L. albus*	LAGI01_11627	2433	2514	90.2	82	6.00E-22	112
KB421708.1	39988	23974	24055	*L. albus*	LAGI01_11540	294	213	90.2	82	6.00E-22	112
KB421708.1	39988	23974	24055	*L. albus*	LAGI01_11535	294	213	90.2	82	6.00E-22	112
KB421708.1	39988	23974	24055	*L. albus*	LAGI01_11534	294	213	90.2	82	6.00E-22	112
KB421708.1	39988	23974	24055	*L. albus*	LAGI01_11536	2441	2522	90.2	82	6.00E-22	112
KB421708.1	39988	20196	20366	*L. albus*	LAGI01_98567	19	188	78.9	171	6.00E-22	112
KB421708.1	39988	48	234	*L. albus*	LAGI01_35371	215	32	79.6	187	6.00E-22	112
KB421708.1	39988	49	234	*L. albus*	LAGI01_35371	254	436	79.5	186	2.00E-21	110
KB425167.1	48639	4836	5695	*L. albus*	LAGI01_32535	1342	485	91.6	860	0	1219
KB425167.1	48639	3455	3932	*L. albus*	LAGI01_54219	565	1042	93.1	479	0	712
KB425167.1	48639	3840	4269	*L. albus*	LAGI01_29482	1098	669	93.7	430	0	671
KB425167.1	48639	14948	15388	*L. albus*	LAGI01_4091	3750	3312	90.5	443	2.00E-157	562
KB425167.1	48639	14955	15388	*L. albus*	LAGI01_19917	15	446	90.5	436	4.00E-155	554
KB425167.1	48639	14955	15388	*L. albus*	LAGI01_4405	27	458	90.5	436	4.00E-155	554
KB425167.1	48639	1941	2290	*L. albus*	LAGI01_54219	155	502	94	350	1.00E-151	542
KB425167.1	48639	1941	2290	*L. albus*	LAGI01_29482	1670	1323	94	350	1.00E-151	542
KB425167.1	48639	1941	2290	*L. albus*	LAGI01_33900	134	481	94	350	1.00E-151	542
KB425167.1	48639	1941	2290	*L. albus*	LAGI01_32708	1540	1193	94	350	1.00E-151	542
KB425167.1	48639	1941	2290	*L. albus*	LAGI01_30117	1648	1301	94	350	1.00E-151	542
KB425167.1	48639	1941	2290	*L. albus*	LAGI01_30051	1629	1282	94	350	1.00E-151	542
KB425167.1	48639	19067	19415	*L. albus*	LAGI01_4091	404	48	92.9	357	1.00E-148	533
KB425167.1	48639	19067	19415	*L. albus*	LAGI01_205	4003	3647	92.9	357	1.00E-148	533
KB425167.1	48639	19067	19414	*L. albus*	LAGI01_200	3389	3744	92.9	356	4.00E-148	531
KB425167.1	48639	14991	15388	*L. albus*	LAGI01_205	39	433	90.9	399	3.00E-145	521
KB425167.1	48639	14991	15388	*L. albus*	LAGI01_205	7305	6911	90.9	399	3.00E-145	521
KB425167.1	48639	14991	15388	*L. albus*	LAGI01_200	7353	6959	90.9	399	3.00E-145	521
KB425167.1	48639	19067	19369	*L. albus*	LAGI01_200	4051	3741	92.6	311	1.00E-125	456
KB425167.1	48639	3455	3739	*L. albus*	LAGI01_30117	1238	955	93.3	285	2.00E-117	429
KB425167.1	48639	19067	19349	*L. albus*	LAGI01_205	3341	3631	92.7	291	2.00E-117	429
KB425167.1	48639	3668	3932	*L. albus*	LAGI01_30051	1128	863	93.2	266	3.00E-108	398
KB425167.1	48639	3668	3932	*L. albus*	LAGI01_65675	471	736	93.2	266	3.00E-108	398
KB425167.1	48639	3668	3932	*L. albus*	LAGI01_62939	542	807	92.4	266	9.00E-105	387
KB425167.1	48639	14964	15388	*L. albus*	LAGI01_200	48	481	83.7	438	3.00E-101	375
KB425167.1	48639	4074	4269	*L. albus*	LAGI01_33900	797	992	95.9	196	6.00E-88	331
KB425167.1	48639	4074	4269	*L. albus*	LAGI01_32708	877	682	95.9	196	6.00E-88	331
KB425167.1	48639	4074	4269	*L. albus*	LAGI01_30051	864	669	95.9	196	6.00E-88	331
KB425167.1	48639	4074	4269	*L. albus*	LAGI01_36054	861	666	95.9	196	6.00E-88	331
KB425167.1	48639	4074	4269	*L. albus*	LAGI01_53501	163	358	92.3	196	8.00E-76	291
KB425167.1	48639	19064	19401	*L. albus*	LAGI01_4405	3363	3705	82.1	353	1.00E-74	287
KB425167.1	48639	6840	7012	*L. albus*	LAGI01_32535	421	249	94.7	173	6.00E-73	281
KB425167.1	48639	6840	7012	*L. albus*	LAGI01_29482	421	249	94.7	173	6.00E-73	281
KB425167.1	48639	6840	7012	*L. albus*	LAGI01_33900	1240	1412	94.7	173	6.00E-73	281
KB425167.1	48639	6840	7012	*L. albus*	LAGI01_30117	421	249	94.7	173	6.00E-73	281
KB425167.1	48639	6840	7012	*L. albus*	LAGI01_30051	421	249	94.7	173	6.00E-73	281
KB425167.1	48639	6840	7012	*L. albus*	LAGI01_36054	418	246	94.7	173	6.00E-73	281
KB425167.1	48639	2128	2290	*L. albus*	LAGI01_65675	155	317	95.7	163	1.00E-70	273
KB425167.1	48639	1988	2290	*L. albus*	LAGI01_36054	1488	1177	85	315	7.00E-69	267
KB425167.1	48639	1988	2290	*L. albus*	LAGI01_62939	77	388	84.7	315	4.00E-67	262
KB425167.1	48639	6843	7012	*L. albus*	LAGI01_32708	431	262	92.3	170	3.00E-64	252
KB425167.1	48639	6843	7012	*L. albus*	LAGI01_53501	661	830	92.3	170	3.00E-64	252
KB425167.1	48639	6843	7012	*L. albus*	LAGI01_55436	609	778	92.3	170	3.00E-64	252
KB425167.1	48639	4074	4269	*L. albus*	LAGI01_32535	1536	1341	88.7	196	1.00E-63	250
KB425167.1	48639	4074	4269	*L. albus*	LAGI01_30117	864	669	88.7	196	1.00E-63	250
KB425167.1	48639	4074	4269	*L. albus*	LAGI01_55436	163	358	88.7	196	1.00E-63	250
KB425167.1	48639	4836	4953	*L. albus*	LAGI01_29482	670	553	98.3	118	3.00E-53	216
KB425167.1	48639	4836	4953	*L. albus*	LAGI01_32708	683	566	98.3	118	3.00E-53	216
KB425167.1	48639	4836	4953	*L. albus*	LAGI01_30117	670	553	98.3	118	3.00E-53	216
KB425167.1	48639	4836	4953	*L. albus*	LAGI01_30051	670	553	98.3	118	3.00E-53	216

Accession	Scaffold	Scaffold start	Scaffold end	Species	EST name	EST start	EST end	Percent identity	Alignment length	E-value	Score
KB425167.1	48639	4836	4953	*L. albus*	LAGI01_36054	667	550	98.3	118	3.00E-53	216
KB425167.1	48639	7686	7827	*L. albus*	LAGI01_32535	150	10	92.2	142	1.00E-48	200
KB425167.1	48639	7686	7827	*L. albus*	LAGI01_29482	150	10	92.2	142	1.00E-48	200
KB425167.1	48639	7686	7827	*L. albus*	LAGI01_32708	163	23	92.2	142	1.00E-48	200
KB425167.1	48639	7686	7827	*L. albus*	LAGI01_30117	150	10	92.2	142	1.00E-48	200
KB425167.1	48639	7686	7827	*L. albus*	LAGI01_30051	150	10	92.2	142	1.00E-48	200
KB425167.1	48639	7686	7827	*L. albus*	LAGI01_36054	147	7	92.2	142	1.00E-48	200
KB425167.1	48639	5571	5695	*L. albus*	LAGI01_53501	470	594	92	125	2.00E-43	183
KB425167.1	48639	4836	4954	*L. albus*	LAGI01_53501	357	475	92.4	119	1.00E-41	177
KB425167.1	48639	4836	4953	*L. albus*	LAGI01_33900	991	1108	92.3	118	4.00E-41	175
KB425167.1	48639	4836	4953	*L. albus*	LAGI01_55436	357	474	92.3	118	4.00E-41	175
KB425167.1	48639	2735	2888	*L. albus*	LAGI01_87791	161	8	87	155	1.00E-37	164
KB425167.1	48639	3840	3932	*L. albus*	LAGI01_32708	968	876	95.6	93	3.00E-35	156
KB425167.1	48639	3840	3932	*L. albus*	LAGI01_30117	955	863	95.6	93	3.00E-35	156
KB425167.1	48639	3840	3932	*L. albus*	LAGI01_36054	952	860	95.6	93	3.00E-35	156
KB425167.1	48639	3455	3548	*L. albus*	LAGI01_29482	1260	1167	94.6	94	4.00E-34	152
KB425167.1	48639	3455	3548	*L. albus*	LAGI01_33900	544	637	94.6	94	4.00E-34	152
KB425167.1	48639	3455	3548	*L. albus*	LAGI01_30051	1219	1126	94.6	94	4.00E-34	152
KB425167.1	48639	3455	3548	*L. albus*	LAGI01_65675	380	473	94.6	94	4.00E-34	152
KB425167.1	48639	3455	3548	*L. albus*	LAGI01_62939	451	544	94.6	94	4.00E-34	152
KB425167.1	48639	3455	3548	*L. albus*	LAGI01_36054	1114	1021	94.6	94	4.00E-34	152
KB425167.1	48639	7454	7554	*L. albus*	LAGI01_32535	252	152	92	101	5.00E-33	148
KB425167.1	48639	7454	7554	*L. albus*	LAGI01_29482	252	152	92	101	5.00E-33	148
KB425167.1	48639	7454	7554	*L. albus*	LAGI01_32708	265	165	92	101	5.00E-33	148
KB425167.1	48639	7454	7554	*L. albus*	LAGI01_30117	252	152	92	101	5.00E-33	148
KB425167.1	48639	7454	7554	*L. albus*	LAGI01_30051	252	152	92	101	5.00E-33	148
KB425167.1	48639	7454	7554	*L. albus*	LAGI01_36054	249	149	92	101	5.00E-33	148
KB425167.1	48639	3840	3932	*L. albus*	LAGI01_32535	1627	1535	93.5	93	8.00E-32	144
KB425167.1	48639	3840	3932	*L. albus*	LAGI01_55436	72	164	93.5	93	8.00E-32	144
KB425167.1	48639	3908	4040	*L. albus*	LAGI01_110242	1	131	86.4	133	3.00E-31	142
KB425167.1	48639	3840	3932	*L. albus*	LAGI01_53501	72	164	92.4	93	4.00E-30	139
KB425167.1	48639	3840	3932	*L. albus*	LAGI01_33900	706	798	91.3	93	2.00E-28	133
KB425167.1	48639	6486	6552	*L. albus*	LAGI01_32535	487	421	100	67	3.00E-27	129
KB425167.1	48639	6486	6552	*L. albus*	LAGI01_29482	487	421	100	67	3.00E-27	129
KB425167.1	48639	6486	6552	*L. albus*	LAGI01_33900	1174	1240	100	67	3.00E-27	129
KB425167.1	48639	3455	3548	*L. albus*	LAGI01_32708	1130	1037	90.4	94	3.00E-27	129
KB425167.1	48639	6486	6552	*L. albus*	LAGI01_30117	487	421	100	67	3.00E-27	129
KB425167.1	48639	6486	6552	*L. albus*	LAGI01_30051	487	421	100	67	3.00E-27	129
KB425167.1	48639	6486	6552	*L. albus*	LAGI01_36054	484	418	100	67	3.00E-27	129
KB425167.1	48639	3668	3739	*L. albus*	LAGI01_29482	1169	1098	97.2	72	1.00E-26	127
KB425167.1	48639	2471	2535	*L. albus*	LAGI01_54219	502	566	100	65	5.00E-26	125
KB425167.1	48639	2471	2535	*L. albus*	LAGI01_29482	1323	1259	100	65	5.00E-26	125
KB425167.1	48639	2471	2535	*L. albus*	LAGI01_33900	481	545	100	65	5.00E-26	125
KB425167.1	48639	2471	2535	*L. albus*	LAGI01_30117	1301	1237	100	65	5.00E-26	125
KB425167.1	48639	2471	2535	*L. albus*	LAGI01_30051	1282	1218	100	65	5.00E-26	125
KB425167.1	48639	2471	2535	*L. albus*	LAGI01_65675	317	381	100	65	5.00E-26	125
KB425167.1	48639	2471	2535	*L. albus*	LAGI01_62939	388	452	100	65	5.00E-26	125
KB425167.1	48639	2471	2535	*L. albus*	LAGI01_36054	1177	1113	100	65	5.00E-26	125
KB425167.1	48639	4017	4101	*L. albus*	LAGI01_124787	84	1	92.9	85	3.00E-24	119
KB425167.1	48639	3668	3739	*L. albus*	LAGI01_32535	1698	1627	94.4	72	4.00E-23	116
KB425167.1	48639	3668	3739	*L. albus*	LAGI01_33900	635	706	94.4	72	4.00E-23	116
KB425167.1	48639	3668	3739	*L. albus*	LAGI01_32708	1039	968	94.4	72	4.00E-23	116
KB425167.1	48639	3668	3739	*L. albus*	LAGI01_36054	1023	952	94.4	72	4.00E-23	116
KB425167.1	48639	3668	3739	*L. albus*	LAGI01_53501	1	72	94.4	72	4.00E-23	116
KB425167.1	48639	3668	3739	*L. albus*	LAGI01_55436	1	72	94.4	72	4.00E-23	116
KB425167.1	48639	2471	2532	*L. albus*	LAGI01_32708	1193	1132	98.3	62	1.00E-22	114
KB425167.1	48639	17061	17178	*L. albus*	LAGI01_61967	627	512	85.7	119	5.00E-22	112
KB425167.1	48639	7454	7552	*L. albus*	LAGI01_33900	1409	1507	85.8	99	2.00E-21	110

Accession	Scaffold	Scaffold start	Scaffold end	Species	EST name	EST start	EST end	Percent identity	Alignment length	E-value	Score
KB425167.1	48639	7454	7552	*L. albus*	LAGI01_53501	827	925	85.8	99	2.00E-21	110
KB425167.1	48639	7454	7552	*L. albus*	LAGI01_55436	775	873	85.8	99	2.00E-21	110
KB430490.1	65565	14607	15564	*L. albus*	LAGI01_32162	1665	699	91.6	967	0	1373
KB430490.1	65565	14607	15564	*L. albus*	LAGI01_33538	1620	654	91.5	967	0	1367
KB430490.1	65565	14607	15564	*L. albus*	LAGI01_46203	1226	273	90.6	958	0	1294
KB430490.1	65565	1581	2179	*L. albus*	LAGI01_30987	31	628	95.1	599	0	975
KB430490.1	65565	7577	8206	*L. albus*	LAGI01_59017	912	303	88.7	633	0	769
KB430490.1	65565	4921	5344	*L. albus*	LAGI01_30987	1324	1748	90.6	429	9.00E-156	556
KB430490.1	65565	15564	15986	*L. albus*	LAGI01_32162	671	263	89.4	425	7.00E-142	510
KB430490.1	65565	9313	9637	*L. albus*	LAGI01_58790	325	1	93.5	325	4.00E-140	504
KB430490.1	65565	2288	2590	*L. albus*	LAGI01_30987	628	930	94.7	303	4.00E-136	490
KB430490.1	65565	9313	9603	*L. albus*	LAGI01_63477	500	790	93.1	291	3.00E-122	444
KB430490.1	65565	9774	10297	*L. albus*	LAGI01_15621	1522	2047	84.2	533	2.00E-116	425
KB430490.1	65565	8867	9153	*L. albus*	LAGI01_60727	469	183	91.2	287	3.00E-111	408
KB430490.1	65565	8797	9094	*L. albus*	LAGI01_34196	731	433	90.3	299	2.00E-108	398
KB430490.1	65565	7950	8209	*L. albus*	LAGI01_73293	1	260	93.4	261	3.00E-104	385
KB430490.1	65565	15564	15855	*L. albus*	LAGI01_33538	626	339	90.4	294	2.00E-101	375
KB430490.1	65565	8867	9094	*L. albus*	LAGI01_73293	260	487	93.4	228	2.00E-94	352
KB430490.1	65565	8867	9099	*L. albus*	LAGI01_60584	397	629	92.2	233	4.00E-92	344
KB430490.1	65565	7577	7787	*L. albus*	LAGI01_58790	917	707	94.3	211	8.00E-90	337
KB430490.1	65565	8867	9094	*L. albus*	LAGI01_59017	300	73	92.1	228	3.00E-89	335
KB430490.1	65565	8867	9094	*L. albus*	LAGI01_58790	551	324	92.1	228	3.00E-89	335
KB430490.1	65565	7979	8206	*L. albus*	LAGI01_63477	43	272	92.1	230	2.00E-87	329
KB430490.1	65565	9313	9623	*L. albus*	LAGI01_34196	434	121	85.6	314	2.00E-86	325
KB430490.1	65565	8867	9094	*L. albus*	LAGI01_63477	275	501	90.7	228	4.00E-81	308
KB430490.1	65565	8053	8243	*L. albus*	LAGI01_34196	1401	1211	91.6	191	3.00E-71	275
KB430490.1	65565	6430	6656	*L. albus*	LAGI01_21702	287	515	86.8	229	3.00E-63	248
KB430490.1	65565	8182	8374	*L. albus*	LAGI01_95800	208	17	89.1	193	7.00E-61	241
KB430490.1	65565	8053	8206	*L. albus*	LAGI01_60727	625	472	92.8	154	1.00E-58	233
KB430490.1	65565	8053	8206	*L. albus*	LAGI01_60584	241	394	92.8	154	1.00E-58	233
KB430490.1	65565	8053	8206	*L. albus*	LAGI01_81059	177	24	92.8	154	1.00E-58	233
KB430490.1	65565	8053	8206	*L. albus*	LAGI01_58790	708	554	92.9	155	3.00E-56	225
KB430490.1	65565	6573	6850	*L. albus*	LAGI01_22399	1935	1655	82.2	282	3.00E-56	225
KB430490.1	65565	3487	3624	*L. albus*	LAGI01_108405	140	3	93.4	138	9.00E-53	214
KB430490.1	65565	6573	6820	*L. albus*	LAGI01_88591	31	281	82.5	252	3.00E-49	202
KB430490.1	65565	16046	16191	*L. albus*	LAGI01_33538	157	13	91	146	1.00E-47	196
KB430490.1	65565	9308	9444	*L. albus*	LAGI01_60584	708	844	91.2	137	6.00E-47	194
KB430490.1	65565	10036	10268	*L. albus*	LAGI01_92824	1	231	83.3	234	1.00E-44	187
KB430490.1	65565	6603	6841	*L. albus*	LAGI01_92181	240	1	81.8	243	4.00E-44	185
KB430490.1	65565	6583	6890	*L. albus*	LAGI01_31187	947	636	79.5	313	4.00E-44	185
KB430490.1	65565	6583	6890	*L. albus*	LAGI01_17327	1532	1221	79.5	313	4.00E-44	185
KB430490.1	65565	6583	6890	*L. albus*	LAGI01_1493	4065	3754	79.5	313	4.00E-44	185
KB430490.1	65565	4476	4573	*L. albus*	LAGI01_30987	1157	1254	98.9	98	2.00E-43	183
KB430490.1	65565	15866	15986	*L. albus*	LAGI01_33538	457	335	91.8	123	1.00E-36	160
KB430490.1	65565	7586	7787	*L. albus*	LAGI01_81059	388	176	82.7	214	1.00E-36	160
KB430490.1	65565	4235	4324	*L. albus*	LAGI01_30987	1070	1159	95.5	90	1.00E-33	150
KB430490.1	65565	512	784	*L. albus*	LAGI01_70925	595	324	78.5	275	1.00E-33	150
KB430490.1	65565	525	780	*L. albus*	LAGI01_36672	1307	1560	78.1	256	1.00E-33	150
KB430490.1	65565	15741	15855	*L. albus*	LAGI01_32162	383	267	91.4	117	4.00E-33	148
KB430490.1	65565	525	773	*L. albus*	LAGI01_66916	257	10	77.5	249	2.00E-32	146
KB430490.1	65565	18063	18167	*L. albus*	LAGI01_87846	186	290	90.4	105	6.00E-32	144
KB430490.1	65565	6660	6944	*L. albus*	LAGI01_74918	508	233	77.8	289	3.00E-30	139
KB430490.1	65565	9968	10104	*L. albus*	LAGI01_108459	1	138	87.1	140	1.00E-29	137
KB430490.1	65565	3186	3267	*L. albus*	LAGI01_30987	930	1011	95.1	82	5.00E-29	135
KB430490.1	65565	4667	4742	*L. albus*	LAGI01_30987	1252	1327	97.3	76	5.00E-29	135
KB430490.1	65565	7703	7796	*L. albus*	LAGI01_71298	447	354	91.4	94	5.00E-29	135
KB430490.1	65565	11664	11830	*L. albus*	LAGI01_4974	3238	3405	81.5	168	5.00E-29	135
KB430490.1	65565	525	773	*L. albus*	LAGI01_51454	844	1089	77.1	249	7.00E-28	131
KB430490.1	65565	7433	7538	*L. albus*	LAGI01_123386	1	104	90.6	107	3.00E-27	129

Accession	Scaffold	Scaffold start	Scaffold end	Species	EST name	EST start	EST end	Percent identity	Alignment length	E-value	Score
KB430490.1	65565	11664	11815	*L. albus*	LAGI01_93109	80	230	82.2	152	1.00E-26	127
KB430490.1	65565	6539	6800	*L. albus*	LAGI01_84368	8	285	78	278	4.00E-26	125
KB430490.1	65565	7703	7787	*L. albus*	LAGI01_60727	708	624	91.7	85	1.00E-25	123
KB430490.1	65565	7703	7787	*L. albus*	LAGI01_34196	1484	1400	91.7	85	1.00E-25	123
KB430490.1	65565	7703	7787	*L. albus*	LAGI01_60584	158	242	91.7	85	1.00E-25	123
KB430490.1	65565	6629	6770	*L. albus*	LAGI01_103260	1	141	83.9	143	1.00E-25	123
KB430490.1	65565	10035	10216	*L. albus*	LAGI01_99268	1	183	80.9	184	1.00E-25	123
KB430490.1	65565	551	784	*L. albus*	LAGI01_11466	2568	2339	76.4	234	1.00E-25	123
KB430490.1	65565	551	784	*L. albus*	LAGI01_10355	2607	2378	76.4	234	1.00E-25	123
KB430490.1	65565	551	784	*L. albus*	LAGI01_10147	2690	2461	76.4	234	1.00E-25	123
KB430490.1	65565	16046	16129	*L. albus*	LAGI01_32162	85	2	91.6	84	6.00E-25	121
KB430490.1	65565	15866	16012	*L. albus*	LAGI01_32162	502	354	84.7	151	2.00E-24	119
KB430490.1	65565	11663	11830	*L. albus*	LAGI01_69620	384	216	79.8	169	2.00E-24	119
KB430490.1	65565	11663	11830	*L. albus*	LAGI01_46586	768	600	79.8	169	2.00E-24	119
KB430490.1	65565	642	768	*L. albus*	LAGI01_32208	573	448	84.2	127	2.00E-24	119
KB430490.1	65565	642	768	*L. albus*	LAGI01_19837	810	685	84.2	127	2.00E-24	119
KB430490.1	65565	11664	11783	*L. albus*	LAGI01_6554	3176	3296	85.1	121	2.00E-24	119
KB430490.1	65565	14838	14939	*L. albus*	LAGI01_105968	34	135	86.2	102	3.00E-23	116
KB430490.1	65565	11670	11830	*L. albus*	LAGI01_47295	796	637	80.1	161	3.00E-23	116
KB430490.1	65565	11661	11830	*L. albus*	LAGI01_15415	1497	1329	79.4	170	3.00E-23	116
KB430490.1	65565	9313	9386	*L. albus*	LAGI01_59017	74	1	93.2	74	1.00E-22	114
KB430490.1	65565	9313	9386	*L. albus*	LAGI01_73293	486	559	93.2	74	1.00E-22	114
KB430490.1	65565	6538	6711	*L. albus*	LAGI01_46653	1	177	81.4	178	1.00E-22	114
KB430490.1	65565	11663	11802	*L. albus*	LAGI01_36826	410	271	80.7	140	1.00E-22	114
KB430490.1	65565	6684	6890	*L. albus*	LAGI01_86792	301	96	76.8	207	4.00E-22	112
KB430490.1	65565	651	794	*L. albus*	LAGI01_91563	246	103	79.8	144	2.00E-21	110
KB430490.1	65565	3879	3937	*L. albus*	LAGI01_30987	1011	1069	98.3	59	6.00E-21	108
KB430490.1	65565	11659	11830	*L. albus*	LAGI01_46028	755	585	78.4	172	6.00E-21	108
KB430490.1	65565	18073	18158	*L. albus*	LAGI01_1967	86	1	88.3	86	6.00E-21	108
KB430490.1	65565	18073	18158	*L. albus*	LAGI01_1920	4508	4593	88.3	86	6.00E-21	108
KB430490.1	65565	512	780	*L. albus*	LAGI01_70925	18	289	75.6	275	2.00E-20	106
KB430490.1	65565	653	790	*L. albus*	LAGI01_91531	104	244	83	142	2.00E-20	106
KB430490.1	65565	18068	18163	*L. albus*	LAGI01_66345	95	1	87.5	96	2.00E-20	106
KB430490.1	65565	11668	11784	*L. albus*	LAGI01_34538	1417	1302	83.7	117	2.00E-20	106
KB412128.1	20923	16548	17223	*L. luteus*	l0itg22510	701	22	92.8	681	0	988
KB412128.1	20923	16548	17223	*L. luteus*	l0itg22509	701	22	92.8	681	0	988
KB412128.1	20923	26002	26635	*L. luteus*	l0itg29770	1	629	94.5	640	0	971
KB412128.1	20923	16648	17205	*L. luteus*	l0itg22925	541	3	87.4	558	1.00E-178	631
KB412128.1	20923	13110	13469	*L. luteus*	l0itg22510	1277	918	96.3	360	2.00E-174	617
KB412128.1	20923	26003	26497	*L. luteus*	l0itg25171	609	123	86.7	498	7.00E-148	529
KB412128.1	20923	25445	25759	*L. luteus*	l0itg25170	709	395	94.2	315	9.00E-140	502
KB412128.1	20923	25999	26363	*L. luteus*	l0itg25170	397	34	90.6	365	5.00E-138	496
KB412128.1	20923	25445	25758	*L. luteus*	l0itg25171	925	612	91.7	314	3.00E-125	454
KB412128.1	20923	30524	30847	*L. luteus*	l0itg00402	154	477	89.5	326	1.00E-109	402
KB412128.1	20923	30524	30847	*L. luteus*	l0itg00401	251	574	89.5	326	1.00E-109	402
KB412128.1	20923	30524	30849	*L. luteus*	l0itg00355	251	576	89.3	328	4.00E-109	400
KB412128.1	20923	30524	30849	*L. luteus*	l0itg00354	154	479	89.3	328	4.00E-109	400
KB412128.1	20923	30524	30849	*L. luteus*	l0itg00350	251	576	89.3	328	4.00E-109	400
KB412128.1	20923	30524	30849	*L. luteus*	l0itg00349	154	479	89.3	328	4.00E-109	400
KB412128.1	20923	16648	16911	*L. luteus*	l0itg22926	397	134	90.9	264	7.00E-100	369
KB412128.1	20923	30504	30849	*L. luteus*	l0itg00367	337	683	85.2	352	1.00E-87	329
KB412128.1	20923	30504	30849	*L. luteus*	l0itg00357	337	683	85.2	352	1.00E-87	329
KB412128.1	20923	30492	30758	*L. luteus*	l0itg18673	237	503	87.3	269	1.00E-76	292
KB412128.1	20923	30491	30713	*L. luteus*	l0itg29672	301	76	89.8	226	8.00E-74	283
KB412128.1	20923	30524	30849	*L. luteus*	l0itg00359	712	1031	83.4	332	8.00E-74	283
KB412128.1	20923	30524	30849	*L. luteus*	l0itg00356	1210	1529	83.4	332	8.00E-74	283
KB412128.1	20923	30524	30849	*L. luteus*	l0itg00353	712	1031	83.4	332	8.00E-74	283

Accession	Scaffold	Scaffold start	Scaffold end	Species	EST name	EST start	EST end	Percent identity	Alignment length	E-value	Score
KB412128.1	20923	30524	30849	*L. luteus*	l0itg00348	1210	1529	83.4	332	8.00E-74	283
KB412128.1	20923	30524	30758	*L. luteus*	l0itg18674	347	581	89.4	237	8.00E-74	283
KB412128.1	20923	30504	30849	*L. luteus*	l0itg00380	337	674	82.6	352	5.00E-72	277
KB412128.1	20923	30504	30849	*L. luteus*	l0itg00360	337	674	82.6	352	5.00E-72	277
KB412128.1	20923	12066	12404	*L. luteus*	l0itg03833	481	148	81.7	339	1.00E-65	256
KB412128.1	20923	12066	12404	*L. luteus*	l0itg03829	481	148	81.7	339	1.00E-65	256
KB412128.1	20923	12066	12404	*L. luteus*	l0itg03826	481	148	81.7	339	1.00E-65	256
KB412128.1	20923	30504	30849	*L. luteus*	l0itg00363	618	965	81.5	353	1.00E-65	256
KB412128.1	20923	30504	30849	*L. luteus*	l0itg00358	618	965	81.5	353	1.00E-65	256
KB412128.1	20923	14782	14916	*L. luteus*	l0itg22510	920	786	99.2	135	4.00E-65	254
KB412128.1	20923	12066	12404	*L. luteus*	l0itg03832	437	106	82	339	2.00E-64	252
KB412128.1	20923	12066	12404	*L. luteus*	l0itg03828	437	106	82	339	2.00E-64	252
KB412128.1	20923	12066	12404	*L. luteus*	l0itg03827	437	106	82	339	2.00E-64	252
KB412128.1	20923	12066	12404	*L. luteus*	l0itg03831	481	148	81.4	339	6.00E-64	250
KB412128.1	20923	12066	12404	*L. luteus*	l0itg03825	482	148	81.7	340	2.00E-63	248
KB412128.1	20923	12066	12404	*L. luteus*	l0itg03823	482	148	81.7	340	2.00E-63	248
KB412128.1	20923	12066	12404	*L. luteus*	l0itg03821	482	148	81.7	340	2.00E-63	248
KB412128.1	20923	12066	12404	*L. luteus*	l0itg03830	437	106	81.7	339	8.00E-63	246
KB412128.1	20923	12066	12404	*L. luteus*	l0itg03824	482	148	81.4	340	1.00E-61	242
KB412128.1	20923	12082	12404	*L. luteus*	l0itg03822	465	148	81.7	323	1.00E-61	242
KB412128.1	20923	12082	12404	*L. luteus*	l0itg03820	421	106	82	323	2.00E-60	239
KB412128.1	20923	24904	25114	*L. luteus*	l0itg25171	1134	924	86.2	211	2.00E-60	239
KB412128.1	20923	24904	25114	*L. luteus*	l0itg25170	918	708	86.2	211	2.00E-60	239
KB412128.1	20923	12082	12404	*L. luteus*	l0itg03819	466	148	81.7	324	2.00E-59	235
KB412128.1	20923	30640	30849	*L. luteus*	l0itg09990	319	109	88.2	212	2.00E-59	235
KB412128.1	20923	30563	30838	*L. luteus*	l0itg51426	462	208	83	278	2.00E-56	225
KB412128.1	20923	30487	30687	*L. luteus*	l0itg19083	208	9	88.1	202	3.00E-55	221
KB412128.1	20923	31048	31214	*L. luteus*	l0itg49594	331	491	91	168	4.00E-54	217
KB412128.1	20923	30669	30847	*L. luteus*	l0itg00396	265	444	87.7	180	8.00E-52	210
KB412128.1	20923	30669	30847	*L. luteus*	l0itg00395	362	541	87.7	180	8.00E-52	210
KB412128.1	20923	30679	30863	*L. luteus*	l0itg47852	470	286	84.8	185	3.00E-47	194
KB412128.1	20923	30487	30709	*L. luteus*	l0itg19082	222	1	84.4	225	1.00E-46	192
KB412128.1	20923	26014	26228	*L. luteus*	l0itg16402	647	433	81.8	215	2.00E-45	189
KB412128.1	20923	30524	30732	*L. luteus*	l0itg21228	280	485	84.2	210	3.00E-44	185
KB412128.1	20923	30662	30866	*L. luteus*	l0itg09641	251	48	82.9	205	1.00E-43	183
KB412128.1	20923	30662	30866	*L. luteus*	l0itg09639	251	48	82.9	205	1.00E-43	183
KB412128.1	20923	14782	14916	*L. luteus*	l0itg22509	920	786	89.6	135	1.00E-42	179
KB412128.1	20923	13347	13474	*L. luteus*	l0itg22509	1040	913	90.6	128	6.00E-42	177
KB412128.1	20923	25451	25747	*L. luteus*	l0itg18148	1232	936	76.7	297	8.00E-41	173
KB412128.1	20923	11984	12183	*L. luteus*	l0itg10638	237	38	81.5	200	3.00E-40	171
KB412128.1	20923	16079	16174	*L. luteus*	l0itg22510	796	701	96.8	96	4.00E-39	167
KB412128.1	20923	11997	12183	*L. luteus*	l0itg10639	224	38	81.8	187	6.00E-38	164
KB412128.1	20923	28977	29141	*L. luteus*	l0itg49594	115	282	87.1	171	6.00E-38	164
KB412128.1	20923	5518	5634	*L. luteus*	l0itg03651	731	615	90.5	117	2.00E-37	162
KB412128.1	20923	5518	5634	*L. luteus*	l0itg03649	964	848	90.5	117	2.00E-37	162
KB412128.1	20923	5518	5634	*L. luteus*	l0itg03647	624	508	90.5	117	2.00E-37	162
KB412128.1	20923	5518	5634	*L. luteus*	l0itg03646	1135	1019	90.5	117	2.00E-37	162
KB412128.1	20923	5518	5634	*L. luteus*	l0itg03645	853	737	90.5	117	2.00E-37	162
KB412128.1	20923	5518	5634	*L. luteus*	l0itg03644	882	766	90.5	117	2.00E-37	162
KB412128.1	20923	26020	26227	*L. luteus*	l0itg18148	905	698	79.8	208	3.00E-36	158
KB412128.1	20923	26020	26227	*L. luteus*	l0itg18149	905	698	79.8	208	3.00E-36	158
KB412128.1	20923	5518	5634	*L. luteus*	l0itg00632	232	116	89.7	117	1.00E-35	156
KB412128.1	20923	5518	5634	*L. luteus*	l0itg00626	232	116	89.7	117	1.00E-35	156
KB412128.1	20923	5518	5634	*L. luteus*	l0itg00613	232	116	89.7	117	1.00E-35	156
KB412128.1	20923	5518	5634	*L. luteus*	l0itg00592	232	116	89.7	117	1.00E-35	156
KB412128.1	20923	5518	5634	*L. luteus*	l0itg00589	232	116	89.7	117	1.00E-35	156
KB412128.1	20923	5518	5634	*L. luteus*	l0itg00587	232	116	89.7	117	1.00E-35	156
KB412128.1	20923	5518	5634	*L. luteus*	l0itg00582	232	116	89.7	117	1.00E-35	156
KB412128.1	20923	7571	7768	*L. luteus*	l0itg10638	238	41	80.3	198	1.00E-35	156

Accession	Scaffold	Scaffold start	Scaffold end	Species	EST name	EST start	EST end	Percent identity	Alignment length	E-value	Score
KB412128.1	20923	30524	30641	*L. luteus*	l0itg00396	154	269	90.6	118	5.00E-35	154
KB412128.1	20923	30524	30641	*L. luteus*	l0itg00395	251	366	90.6	118	5.00E-35	154
KB412128.1	20923	5518	5634	*L. luteus*	l0itg00618	404	287	89.8	118	3.00E-33	148
KB412128.1	20923	5518	5634	*L. luteus*	l0itg00607	404	287	89.8	118	3.00E-33	148
KB412128.1	20923	5518	5634	*L. luteus*	l0itg00601	404	287	89.8	118	3.00E-33	148
KB412128.1	20923	5518	5634	*L. luteus*	l0itg00600	404	287	89.8	118	3.00E-33	148
KB412128.1	20923	5518	5634	*L. luteus*	l0itg00593	404	287	89.8	118	3.00E-33	148
KB412128.1	20923	5518	5634	*L. luteus*	l0itg00591	404	287	89.8	118	3.00E-33	148
KB412128.1	20923	5518	5634	*L. luteus*	l0itg00585	404	287	89.8	118	3.00E-33	148
KB412128.1	20923	5518	5634	*L. luteus*	l0itg00583	404	287	89.8	118	3.00E-33	148
KB412128.1	20923	5518	5634	*L. luteus*	l0itg00581	404	287	89.8	118	3.00E-33	148
KB412128.1	20923	5518	5634	*L. luteus*	l0itg00580	404	287	89.8	118	3.00E-33	148
KB412128.1	20923	5518	5634	*L. luteus*	l0itg00579	404	287	89.8	118	3.00E-33	148
KB412128.1	20923	5518	5634	*L. luteus*	l0itg00577	404	287	89.8	118	3.00E-33	148
KB412128.1	20923	5518	5634	*L. luteus*	l0itg00576	404	287	89.8	118	3.00E-33	148
KB412128.1	20923	5518	5634	*L. luteus*	l0itg00575	404	287	89.8	118	3.00E-33	148
KB412128.1	20923	5518	5634	*L. luteus*	l0itg00574	404	287	89.8	118	3.00E-33	148
KB412128.1	20923	30524	30714	*L. luteus*	l0itg00408	367	547	83.4	193	3.00E-33	148
KB412128.1	20923	30524	30714	*L. luteus*	l0itg00405	342	522	83.4	193	3.00E-33	148
KB412128.1	20923	30640	30743	*L. luteus*	l0itg09987	566	463	94.2	105	3.00E-33	148
KB412128.1	20923	30640	30743	*L. luteus*	l0itg09986	528	425	94.2	105	3.00E-33	148
KB412128.1	20923	32929	33066	*L. luteus*	l0itg36404	414	548	87.6	138	3.00E-33	148
KB412128.1	20923	30524	30744	*L. luteus*	l0itg00383	342	555	81.4	227	1.00E-32	146
KB412128.1	20923	30524	30744	*L. luteus*	l0itg00379	342	555	81.4	227	1.00E-32	146
KB412128.1	20923	30524	30744	*L. luteus*	l0itg00376	342	555	81.4	227	1.00E-32	146
KB412128.1	20923	30524	30744	*L. luteus*	l0itg00375	367	580	81.4	227	1.00E-32	146
KB412128.1	20923	30524	30744	*L. luteus*	l0itg00373	367	580	81.4	227	1.00E-32	146
KB412128.1	20923	30524	30744	*L. luteus*	l0itg00372	367	580	81.4	227	1.00E-32	146
KB412128.1	20923	30524	30744	*L. luteus*	l0itg00368	342	555	81.4	227	1.00E-32	146
KB412128.1	20923	30524	30744	*L. luteus*	l0itg00366	342	555	81.4	227	1.00E-32	146
KB412128.1	20923	30524	30744	*L. luteus*	l0itg00365	367	580	81.4	227	1.00E-32	146
KB412128.1	20923	30524	30744	*L. luteus*	l0itg00364	367	580	81.4	227	1.00E-32	146
KB412128.1	20923	7569	7831	*L. luteus*	l0itg03827	519	262	78.7	263	4.00E-32	144
KB412128.1	20923	220	326	*L. luteus*	l0itg03526	489	383	89.7	107	1.00E-31	142
KB412128.1	20923	220	326	*L. luteus*	l0itg03519	489	383	89.7	107	1.00E-31	142
KB412128.1	20923	220	326	*L. luteus*	l0itg03518	489	383	89.7	107	1.00E-31	142
KB412128.1	20923	7577	7831	*L. luteus*	l0itg03830	510	262	79.6	255	1.00E-31	142
KB412128.1	20923	7585	7768	*L. luteus*	l0itg10639	224	41	79.8	184	6.00E-31	141
KB412128.1	20923	30524	30744	*L. luteus*	l0itg00391	342	556	81.5	228	2.00E-30	139
KB412128.1	20923	30524	30744	*L. luteus*	l0itg00390	367	581	81.5	228	2.00E-30	139
KB412128.1	20923	30553	30662	*L. luteus*	l0itg50136	299	407	90	110	2.00E-30	139
KB412128.1	20923	7569	7831	*L. luteus*	l0itg03832	520	262	78.7	264	8.00E-30	137
KB412128.1	20923	7569	7831	*L. luteus*	l0itg03828	520	262	78.7	264	8.00E-30	137
KB412128.1	20923	7569	7823	*L. luteus*	l0itg03821	564	315	78.4	255	3.00E-29	135
KB412128.1	20923	226	353	*L. luteus*	l0itg03527	517	393	85.9	128	1.00E-28	133
KB412128.1	20923	226	353	*L. luteus*	l0itg03520	486	362	85.9	128	1.00E-28	133
KB412128.1	20923	7577	7823	*L. luteus*	l0itg03824	555	315	79.3	247	1.00E-28	133
KB412128.1	20923	220	326	*L. luteus*	l0itg01455	160	54	87.8	107	4.00E-28	131
KB412128.1	20923	220	326	*L. luteus*	l0itg01451	160	54	87.8	107	4.00E-28	131
KB412128.1	20923	30511	30617	*L. luteus*	l0itg09643	107	1	87.8	107	4.00E-28	131
KB412128.1	20923	30511	30617	*L. luteus*	l0itg09640	107	1	87.8	107	4.00E-28	131
KB412128.1	20923	30524	30643	*L. luteus*	l0itg00392	367	484	88.3	120	4.00E-28	131
KB412128.1	20923	30524	30643	*L. luteus*	l0itg00388	342	459	88.3	120	4.00E-28	131
KB412128.1	20923	30524	30643	*L. luteus*	l0itg00371	342	459	88.3	120	4.00E-28	131
KB412128.1	20923	30524	30643	*L. luteus*	l0itg00370	367	484	88.3	120	4.00E-28	131
KB412128.1	20923	7671	7831	*L. luteus*	l0itg03820	420	262	82.6	161	2.00E-27	129
KB412128.1	20923	30765	30864	*L. luteus*	l0itg02183	744	645	89	100	2.00E-27	129
KB412128.1	20923	30765	30864	*L. luteus*	l0itg02182	712	613	89	100	2.00E-27	129

Accession	Scaffold	Scaffold start	Scaffold end	Species	EST name	EST start	EST end	Percent identity	Alignment length	E-value	Score
KB412128.1	20923	30765	30864	*L. luteus*	l0itg02173	42	141	89	100	2.00E-27	129
KB412128.1	20923	30765	30864	*L. luteus*	l0itg02171	42	141	89	100	2.00E-27	129
KB412128.1	20923	7569	7823	*L. luteus*	l0itg03825	565	315	78.5	256	6.00E-27	127
KB412128.1	20923	7569	7823	*L. luteus*	l0itg03823	565	315	78.5	256	6.00E-27	127
KB412128.1	20923	16079	16174	*L. luteus*	l0itg22509	796	701	89.5	96	6.00E-27	127
KB412128.1	20923	7569	7823	*L. luteus*	l0itg03826	563	314	77.6	255	9.00E-26	123
KB412128.1	20923	25443	25557	*L. luteus*	l0itg55603	374	488	85.2	115	9.00E-26	123
KB412128.1	20923	30487	30627	*L. luteus*	l0itg26912	381	521	83.9	143	9.00E-26	123
KB412128.1	20923	7577	7823	*L. luteus*	l0itg03831	554	314	78.5	247	3.00E-25	121
KB412128.1	20923	7602	7738	*L. luteus*	l0itg37581	378	516	84.1	139	3.00E-25	121
KB412128.1	20923	7671	7823	*L. luteus*	l0itg03819	465	315	82.3	153	1.00E-24	119
KB412128.1	20923	5563	5634	*L. luteus*	l0itg00630	358	287	94.4	72	2.00E-23	116
KB412128.1	20923	7569	7823	*L. luteus*	l0itg03833	564	314	77.7	256	2.00E-23	116
KB412128.1	20923	7569	7823	*L. luteus*	l0itg03829	564	314	77.7	256	2.00E-23	116
KB412128.1	20923	249	383	*L. luteus*	l0itg01417	1126	997	83.7	135	7.00E-23	114
KB412128.1	20923	249	383	*L. luteus*	l0itg01416	1231	1102	83.7	135	7.00E-23	114
KB412128.1	20923	249	383	*L. luteus*	l0itg01414	1294	1165	83.7	135	7.00E-23	114
KB412128.1	20923	249	383	*L. luteus*	l0itg01413	1126	997	83.7	135	7.00E-23	114
KB412128.1	20923	249	383	*L. luteus*	l0itg01411	1231	1102	83.7	135	7.00E-23	114
KB412128.1	20923	249	383	*L. luteus*	l0itg01410	1294	1165	83.7	135	7.00E-23	114
KB412128.1	20923	249	383	*L. luteus*	l0itg01409	1155	1026	83.7	135	7.00E-23	114
KB412128.1	20923	249	383	*L. luteus*	l0itg01407	1155	1026	83.7	135	7.00E-23	114
KB412128.1	20923	249	383	*L. luteus*	l0itg01397	1423	1294	83.7	135	7.00E-23	114
KB412128.1	20923	249	383	*L. luteus*	l0itg01396	1423	1294	83.7	135	7.00E-23	114
KB412128.1	20923	249	383	*L. luteus*	l0itg01395	1655	1526	83.7	135	7.00E-23	114
KB412128.1	20923	249	383	*L. luteus*	l0itg01392	1655	1526	83.7	135	7.00E-23	114
KB412128.1	20923	249	383	*L. luteus*	l0itg01445	764	635	83.7	135	7.00E-23	114
KB412128.1	20923	249	383	*L. luteus*	l0itg01440	764	635	83.7	135	7.00E-23	114
KB412128.1	20923	249	383	*L. luteus*	l0itg01399	1969	1840	83.7	135	7.00E-23	114
KB412128.1	20923	249	383	*L. luteus*	l0itg01398	1969	1840	83.7	135	7.00E-23	114
KB412128.1	20923	249	383	*L. luteus*	l0itg01394	1838	1709	83.7	135	7.00E-23	114
KB412128.1	20923	249	383	*L. luteus*	l0itg01393	1838	1709	83.7	135	7.00E-23	114
KB412128.1	20923	249	383	*L. luteus*	l0itg01391	1800	1671	83.7	135	7.00E-23	114
KB412128.1	20923	249	383	*L. luteus*	l0itg01390	1800	1671	83.7	135	7.00E-23	114
KB412128.1	20923	5561	5634	*L. luteus*	l0itg00633	360	287	93.2	74	7.00E-23	114
KB412128.1	20923	12014	12158	*L. luteus*	l0itg37581	378	524	82.3	147	7.00E-23	114
KB412128.1	20923	5563	5634	*L. luteus*	l0itg00621	358	287	93	72	1.00E-21	110
KB412128.1	20923	5563	5634	*L. luteus*	l0itg00623	358	287	93	72	1.00E-21	110
KB412128.1	20923	5561	5634	*L. luteus*	l0itg25361	619	546	91.8	74	4.00E-21	108
KB412128.1	20923	5561	5634	*L. luteus*	l0itg25360	303	230	91.8	74	4.00E-21	108
KB412128.1	20923	7671	7823	*L. luteus*	l0itg03822	464	314	81	153	4.00E-21	108
KB412128.1	20923	30662	30840	*L. luteus*	l0itg50330	796	618	77	179	4.00E-21	108
KB412128.1	20923	249	353	*L. luteus*	l0itg03523	494	393	85.7	105	1.00E-20	106
KB412128.1	20923	249	353	*L. luteus*	l0itg03522	494	393	85.7	105	1.00E-20	106
KB412128.1	20923	249	353	*L. luteus*	l0itg03517	463	362	85.7	105	1.00E-20	106
KB412128.1	20923	249	353	*L. luteus*	l0itg03516	463	362	85.7	105	1.00E-20	106
KB412128.1	20923	7828	7970	*L. luteus*	l0itg03833	208	67	80.4	143	6.00E-20	104
KB412128.1	20923	7828	7970	*L. luteus*	l0itg03829	208	67	80.4	143	6.00E-20	104
KB412128.1	20923	7828	7970	*L. luteus*	l0itg03826	208	67	80.4	143	6.00E-20	104
KB412128.1	20923	7828	7970	*L. luteus*	l0itg03831	208	67	80.4	143	6.00E-20	104
KB412128.1	20923	7828	7970	*L. luteus*	l0itg03825	208	67	80.4	143	6.00E-20	104
KB412128.1	20923	7828	7970	*L. luteus*	l0itg03823	208	67	80.4	143	6.00E-20	104
KB412128.1	20923	7828	7970	*L. luteus*	l0itg03821	208	67	80.4	143	6.00E-20	104
KB412128.1	20923	7828	7970	*L. luteus*	l0itg03824	208	67	80.4	143	6.00E-20	104
KB412128.1	20923	7828	7970	*L. luteus*	l0itg03822	208	67	80.4	143	6.00E-20	104
KB412128.1	20923	7828	7970	*L. luteus*	l0itg03819	208	67	80.4	143	6.00E-20	104
KB412128.1	20923	19866	19973	*L. luteus*	l0itg48438	268	375	83.3	108	6.00E-20	104
KB412128.1	20923	30504	30601	*L. luteus*	l0itg24621	102	6	86.7	98	6.00E-20	104
KB412128.1	20923	30504	30601	*L. luteus*	l0itg24620	102	6	86.7	98	6.00E-20	104

Accession	Scaffold	Scaffold start	Scaffold end	Species	EST name	EST start	EST end	Percent identity	Alignment length	E-value	Score
KB421708.1	39988	18454	18712	*L. luteus*	l0itg54819	340	82	98.4	259	8.00E-132	475
KB421708.1	39988	3314	3607	*L. luteus*	l0itg08410	559	852	93.8	294	9.00E-128	462
KB421708.1	39988	3314	3604	*L. luteus*	l0itg08411	291	1	93.8	291	5.00E-126	456
KB421708.1	39988	6284	6657	*L. luteus*	l0itg46529	396	19	87.8	378	6.00E-122	442
KB421708.1	39988	7349	7762	*L. luteus*	l0itg50196	788	378	85.3	416	3.00E-109	400
KB421708.1	39988	2981	3208	*L. luteus*	l0itg08410	333	560	96	228	3.00E-105	387
KB421708.1	39988	2981	3208	*L. luteus*	l0itg08411	517	290	96	228	3.00E-105	387
KB421708.1	39988	5682	5998	*L. luteus*	l0itg46529	985	682	88.6	317	5.00E-104	383
KB421708.1	39988	7183	7638	*L. luteus*	l0itg03546	289	754	83.1	469	6.00E-100	369
KB421708.1	39988	2981	3208	*L. luteus*	l0itg40676	309	536	93.4	228	9.00E-95	352
KB421708.1	39988	15653	15915	*L. luteus*	l0itg54819	1142	871	90.8	272	3.00E-94	350
KB421708.1	39988	7183	7577	*L. luteus*	l0itg03536	289	689	83.6	404	1.00E-93	348
KB421708.1	39988	7183	7577	*L. luteus*	l0itg03535	289	689	83.6	404	1.00E-93	348
KB421708.1	39988	3314	3613	*L. luteus*	l0itg40676	535	831	87.3	300	1.00E-90	339
KB421708.1	39988	7197	7638	*L. luteus*	l0itg03538	95	549	81.7	456	2.00E-88	331
KB421708.1	39988	11625	11872	*L. luteus*	l0itg14074	461	215	89.5	248	2.00E-84	317
KB421708.1	39988	11625	11872	*L. luteus*	l0itg14073	567	321	89.5	248	2.00E-84	317
KB421708.1	39988	7197	7577	*L. luteus*	l0itg03533	95	484	82	391	5.00E-82	310
KB421708.1	39988	7197	7577	*L. luteus*	l0itg03529	95	484	82	391	5.00E-82	310
KB421708.1	39988	7197	7638	*L. luteus*	l0itg03537	95	549	80.7	456	1.00E-79	302
KB421708.1	39988	7185	7638	*L. luteus*	l0itg03539	123	576	80.5	462	8.00E-77	292
KB421708.1	39988	22149	22305	*L. luteus*	l0itg44728	378	222	98.7	157	3.00E-76	291
KB421708.1	39988	7259	7609	*L. luteus*	l0itg03541	3	362	82.9	363	6.00E-74	283
KB421708.1	39988	7259	7609	*L. luteus*	l0itg03540	3	362	82.9	363	6.00E-74	283
KB421708.1	39988	7197	7577	*L. luteus*	l0itg03534	95	484	80.8	391	2.00E-73	281
KB421708.1	39988	7197	7577	*L. luteus*	l0itg03531	95	484	80.8	391	2.00E-73	281
KB421708.1	39988	2286	2442	*L. luteus*	l0itg08410	177	333	97.4	157	9.00E-73	279
KB421708.1	39988	2286	2442	*L. luteus*	l0itg08411	673	517	97.4	157	9.00E-73	279
KB421708.1	39988	11660	11872	*L. luteus*	l0itg03546	5	216	90.1	213	9.00E-73	279
KB421708.1	39988	11660	11872	*L. luteus*	l0itg03536	5	216	90.1	213	9.00E-73	279
KB421708.1	39988	11660	11872	*L. luteus*	l0itg03535	5	216	90.1	213	9.00E-73	279
KB421708.1	39988	17430	17581	*L. luteus*	l0itg54819	733	582	98	152	1.00E-71	275
KB421708.1	39988	7185	7577	*L. luteus*	l0itg03532	123	511	80.6	397	2.00E-70	271
KB421708.1	39988	7185	7577	*L. luteus*	l0itg03530	123	511	80.6	397	2.00E-70	271
KB421708.1	39988	7259	7573	*L. luteus*	l0itg03549	3	322	82.6	323	7.00E-70	269
KB421708.1	39988	16532	16672	*L. luteus*	l0itg54819	871	731	98.5	141	5.00E-67	260
KB421708.1	39988	7184	7516	*L. luteus*	l0itg35966	413	73	81.9	343	3.00E-65	254
KB421708.1	39988	2286	2439	*L. luteus*	l0itg40676	153	306	94.8	154	4.00E-64	250
KB421708.1	39988	5599	5998	*L. luteus*	l0itg30081	590	192	80	407	2.00E-63	248
KB421708.1	39988	17722	17844	*L. luteus*	l0itg54819	583	461	100	123	5.00E-60	237
KB421708.1	39988	7593	7762	*L. luteus*	l0itg03536	755	924	87	170	5.00E-49	200
KB421708.1	39988	7593	7762	*L. luteus*	l0itg03535	755	924	87	170	5.00E-49	200
KB421708.1	39988	7593	7762	*L. luteus*	l0itg03533	550	719	87	170	5.00E-49	200
KB421708.1	39988	7593	7762	*L. luteus*	l0itg03529	550	719	87	170	5.00E-49	200
KB421708.1	39988	7593	7762	*L. luteus*	l0itg03541	392	561	87	170	5.00E-49	200
KB421708.1	39988	7593	7762	*L. luteus*	l0itg03540	392	561	87	170	5.00E-49	200
KB421708.1	39988	7593	7762	*L. luteus*	l0itg03534	550	719	87	170	5.00E-49	200
KB421708.1	39988	7593	7762	*L. luteus*	l0itg03531	550	719	87	170	5.00E-49	200
KB421708.1	39988	7593	7762	*L. luteus*	l0itg03532	577	746	87	170	5.00E-49	200
KB421708.1	39988	7593	7762	*L. luteus*	l0itg03530	577	746	87	170	5.00E-49	200
KB421708.1	39988	12134	12374	*L. luteus*	l0itg03546	511	755	83.3	246	2.00E-48	198
KB421708.1	39988	17954	18076	*L. luteus*	l0itg54819	462	340	93.4	123	4.00E-46	191
KB421708.1	39988	12134	12314	*L. luteus*	l0itg03536	511	690	85.6	181	1.00E-45	189
KB421708.1	39988	12134	12314	*L. luteus*	l0itg03535	511	690	85.6	181	1.00E-45	189
KB421708.1	39988	12134	12373	*L. luteus*	l0itg50196	742	502	82.7	243	5.00E-45	187
KB421708.1	39988	12134	12374	*L. luteus*	l0itg03538	303	550	82.6	248	3.00E-43	181
KB421708.1	39988	12149	12374	*L. luteus*	l0itg03539	347	577	81.8	231	6.00E-41	173
KB421708.1	39988	5842	5987	*L. luteus*	l0itg05254	144	289	86.9	146	2.00E-40	171

Accession	Scaffold	Scaffold start	Scaffold end	Species	EST name	EST start	EST end	Percent identity	Alignment length	E-value	Score
KB421708.1	39988	5842	5987	*L. luteus*	l0itg05259	144	289	86.9	146	2.00E-40	171
KB421708.1	39988	12134	12314	*L. luteus*	l0itg03533	303	485	84.6	183	2.00E-40	171
KB421708.1	39988	12134	12314	*L. luteus*	l0itg03529	303	485	84.6	183	2.00E-40	171
KB421708.1	39988	51	246	*L. luteus*	l0itg05543	76	270	82.1	196	1.00E-38	166
KB421708.1	39988	7239	7419	*L. luteus*	l0itg32363	230	51	83.4	181	1.00E-38	166
KB421708.1	39988	7552	7762	*L. luteus*	l0itg03548	2	214	81.6	213	1.00E-38	166
KB421708.1	39988	7552	7762	*L. luteus*	l0itg03545	2	214	81.6	213	1.00E-38	166
KB421708.1	39988	12149	12314	*L. luteus*	l0itg03532	347	512	83.7	166	5.00E-38	164
KB421708.1	39988	12149	12314	*L. luteus*	l0itg03530	347	512	83.7	166	5.00E-38	164
KB421708.1	39988	38	247	*L. luteus*	l0itg37414	78	287	81.5	211	2.00E-37	162
KB421708.1	39988	51	247	*L. luteus*	l0itg01622	604	409	81.7	197	2.00E-37	162
KB421708.1	39988	51	247	*L. luteus*	l0itg01618	340	145	81.7	197	2.00E-37	162
KB421708.1	39988	13072	13386	*L. luteus*	l0itg26186	601	287	76.5	316	2.00E-37	162
KB421708.1	39988	13072	13386	*L. luteus*	l0itg26185	35	349	76.5	316	2.00E-37	162
KB421708.1	39988	1940	2024	*L. luteus*	l0itg08410	94	178	98.8	85	3.00E-36	158
KB421708.1	39988	1940	2024	*L. luteus*	l0itg08411	756	672	98.8	85	3.00E-36	158
KB421708.1	39988	12134	12374	*L. luteus*	l0itg03537	303	550	81	248	3.00E-36	158
KB421708.1	39988	51	247	*L. luteus*	l0itg05542	76	271	81.2	197	1.00E-35	156
KB421708.1	39988	51	247	*L. luteus*	l0itg05539	76	271	80.7	197	5.00E-34	150
KB421708.1	39988	51	247	*L. luteus*	l0itg05538	76	271	80.7	197	5.00E-34	150
KB421708.1	39988	51	244	*L. luteus*	l0itg01626	433	241	80.9	194	5.00E-34	150
KB421708.1	39988	19399	19482	*L. luteus*	l0itg54819	84	1	97.6	84	5.00E-34	150
KB421708.1	39988	12134	12314	*L. luteus*	l0itg03534	303	485	82.5	183	2.00E-33	148
KB421708.1	39988	12134	12314	*L. luteus*	l0itg03531	303	485	82.5	183	2.00E-33	148
KB421708.1	39988	22768	22850	*L. luteus*	l0itg44728	167	85	97.5	83	2.00E-33	148
KB421708.1	39988	1	195	*L. luteus*	l0itg35522	175	368	80.5	195	8.00E-33	146
KB421708.1	39988	12134	12344	*L. luteus*	l0itg03541	150	362	81	216	4.00E-31	141
KB421708.1	39988	12134	12344	*L. luteus*	l0itg03540	150	362	81	216	4.00E-31	141
KB421708.1	39988	7183	7365	*L. luteus*	l0itg14073	248	69	82.2	186	2.00E-30	139
KB421708.1	39988	7183	7323	*L. luteus*	l0itg14074	142	2	85.9	142	2.00E-30	139
KB421708.1	39988	111	246	*L. luteus*	l0itg05541	413	547	84.5	136	3.00E-28	131
KB421708.1	39988	6407	6601	*L. luteus*	l0itg05259	712	907	79.7	198	3.00E-28	131
KB421708.1	39988	12134	12252	*L. luteus*	l0itg35966	191	73	88.3	120	3.00E-28	131
KB421708.1	39988	12134	12309	*L. luteus*	l0itg03549	150	322	81.2	176	1.00E-27	129
KB421708.1	39988	51	246	*L. luteus*	l0itg01629	891	698	78.5	196	2.00E-26	125
KB421708.1	39988	111	247	*L. luteus*	l0itg05540	413	548	83.2	137	3.00E-25	121
KB421708.1	39988	11765	11872	*L. luteus*	l0itg14075	320	215	88.8	108	1.00E-24	119
KB421708.1	39988	1940	2024	*L. luteus*	l0itg40676	70	154	90.5	85	4.00E-24	117
KB421708.1	39988	7184	7387	*L. luteus*	l0itg17994	128	341	78.3	217	4.00E-24	117
KB421708.1	39988	79	246	*L. luteus*	l0itg56419	1144	978	80.4	169	1.00E-23	116
KB421708.1	39988	111	247	*L. luteus*	l0itg05537	413	548	82.4	137	1.00E-23	116
KB421708.1	39988	111	247	*L. luteus*	l0itg05536	413	548	82.4	137	1.00E-23	116
KB421708.1	39988	23126	23194	*L. luteus*	l0itg44728	83	15	95.6	69	1.00E-23	116
KB421708.1	39988	136	247	*L. luteus*	l0itg44998	3	114	83.9	112	2.00E-22	112
KB421708.1	39988	22607	22664	*L. luteus*	l0itg44728	222	165	100	58	2.00E-22	112
KB421708.1	39988	2782	2902	*L. luteus*	l0itg46146	64	185	83.6	122	8.00E-22	110
KB421708.1	39988	11625	11735	*L. luteus*	l0itg03535	3885	3994	84.6	111	1.00E-20	106
KB421708.1	39988	11625	11735	*L. luteus*	l0itg03529	3680	3789	84.6	111	1.00E-20	106
KB421708.1	39988	11625	11735	*L. luteus*	l0itg03540	3522	3631	84.6	111	1.00E-20	106
KB421708.1	39988	11625	11735	*L. luteus*	l0itg03531	3680	3789	84.6	111	1.00E-20	106
KB421708.1	39988	11625	11735	*L. luteus*	l0itg03530	3707	3816	84.6	111	1.00E-20	106
KB421708.1	39988	11625	11735	*L. luteus*	l0itg03545	3175	3284	84.6	111	1.00E-20	106
KB421708.1	39988	11625	11735	*L. luteus*	l0itg03550	1049	1158	84.6	111	1.00E-20	106
KB421708.1	39988	1738	1831	*L. luteus*	l0itg08410	2	93	89.3	94	4.00E-20	104
KB421708.1	39988	1738	1831	*L. luteus*	l0itg08411	848	757	89.3	94	4.00E-20	104
KB421708.1	39988	7183	7323	*L. luteus*	l0itg14075	142	2	81.6	142	4.00E-20	104
KB425167.1	48639	4384	4952	*L. luteus*	l0itg22568	1101	536	91.3	569	0	792
KB425167.1	48639	19064	19342	*L. luteus*	l0itg41810	987	1265	97.8	279	6.00E-140	502
KB425167.1	48639	2022	2290	*L. luteus*	l0itg22569	1430	1162	97.3	269	2.00E-132	477

Accession	Scaffold	Scaffold start	Scaffold end	Species	EST name	EST start	EST end	Percent identity	Alignment length	E-value	Score
KB425167.1	48639	3840	4143	*L. luteus*	l0itg46909	294	5	87.1	304	2.00E-88	331
KB425167.1	48639	4074	4269	*L. luteus*	l0itg22569	846	651	94.3	196	3.00E-83	314
KB425167.1	48639	6840	7012	*L. luteus*	l0itg22568	403	231	94.2	173	1.00E-71	275
KB425167.1	48639	6840	7012	*L. luteus*	l0itg22569	403	231	94.2	173	1.00E-71	275
KB425167.1	48639	2009	2288	*L. luteus*	l0itg32767	1410	1127	84	288	4.00E-64	250
KB425167.1	48639	6843	7012	*L. luteus*	l0itg32767	363	194	91.7	170	6.00E-63	246
KB425167.1	48639	4074	4269	*L. luteus*	l0itg32767	809	614	87.2	196	6.00E-59	233
KB425167.1	48639	11070	11420	*L. luteus*	l0itg00621	19	365	79.2	356	6.00E-52	210
KB425167.1	48639	11070	11413	*L. luteus*	l0itg00618	19	358	79.3	349	2.00E-51	208
KB425167.1	48639	11070	11413	*L. luteus*	l0itg00623	19	358	79.3	349	2.00E-51	208
KB425167.1	48639	11070	11413	*L. luteus*	l0itg00607	19	358	79.3	349	2.00E-51	208
KB425167.1	48639	11070	11413	*L. luteus*	l0itg00601	19	358	79.3	349	2.00E-51	208
KB425167.1	48639	11070	11413	*L. luteus*	l0itg00600	19	358	79.3	349	2.00E-51	208
KB425167.1	48639	11070	11413	*L. luteus*	l0itg00593	19	358	79.3	349	2.00E-51	208
KB425167.1	48639	11070	11413	*L. luteus*	l0itg00591	19	358	79.3	349	2.00E-51	208
KB425167.1	48639	11070	11413	*L. luteus*	l0itg00585	19	358	79.3	349	2.00E-51	208
KB425167.1	48639	11070	11413	*L. luteus*	l0itg00583	19	358	79.3	349	2.00E-51	208
KB425167.1	48639	11070	11413	*L. luteus*	l0itg00581	19	358	79.3	349	2.00E-51	208
KB425167.1	48639	11070	11413	*L. luteus*	l0itg00580	19	358	79.3	349	2.00E-51	208
KB425167.1	48639	11070	11413	*L. luteus*	l0itg00579	19	358	79.3	349	2.00E-51	208
KB425167.1	48639	11070	11413	*L. luteus*	l0itg00577	19	358	79.3	349	2.00E-51	208
KB425167.1	48639	11070	11413	*L. luteus*	l0itg00576	19	358	79.3	349	2.00E-51	208
KB425167.1	48639	11070	11413	*L. luteus*	l0itg00575	19	358	79.3	349	2.00E-51	208
KB425167.1	48639	11070	11413	*L. luteus*	l0itg00574	19	358	79.3	349	2.00E-51	208
KB425167.1	48639	11070	11413	*L. luteus*	l0itg00633	19	358	79	349	1.00E-49	202
KB425167.1	48639	4836	4952	*L. luteus*	l0itg22569	652	536	95.7	117	6.00E-48	196
KB425167.1	48639	11070	11413	*L. luteus*	l0itg00630	19	358	78.7	349	6.00E-48	196
KB425167.1	48639	11032	11413	*L. luteus*	l0itg03651	311	686	77.9	386	2.00E-47	194
KB425167.1	48639	11032	11413	*L. luteus*	l0itg03647	204	579	77.9	386	2.00E-47	194
KB425167.1	48639	11032	11413	*L. luteus*	l0itg03645	433	808	77.9	386	2.00E-47	194
KB425167.1	48639	11070	11413	*L. luteus*	l0itg03649	581	919	78.4	348	1.00E-45	189
KB425167.1	48639	11070	11413	*L. luteus*	l0itg03646	752	1090	78.4	348	1.00E-45	189
KB425167.1	48639	11070	11413	*L. luteus*	l0itg03644	498	837	78.2	349	2.00E-44	185
KB425167.1	48639	11033	11326	*L. luteus*	l0itg32588	318	22	78.8	298	3.00E-43	181
KB425167.1	48639	7685	7818	*L. luteus*	l0itg22568	133	1	90.2	134	5.00E-41	173
KB425167.1	48639	7685	7818	*L. luteus*	l0itg22569	133	1	90.2	134	5.00E-41	173
KB425167.1	48639	3840	3932	*L. luteus*	l0itg22569	937	845	97.8	93	3.00E-39	167
KB425167.1	48639	4836	4953	*L. luteus*	l0itg32767	615	498	90.6	118	4.00E-38	164
KB425167.1	48639	3455	3548	*L. luteus*	l0itg22569	1099	1006	94.6	94	1.00E-34	152
KB425167.1	48639	11070	11324	*L. luteus*	l0itg49947	450	194	79.1	259	2.00E-33	148
KB425167.1	48639	3840	3932	*L. luteus*	l0itg32767	900	808	93.5	93	3.00E-32	144
KB425167.1	48639	7454	7554	*L. luteus*	l0itg22568	234	134	91	101	1.00E-31	142
KB425167.1	48639	7454	7554	*L. luteus*	l0itg22569	234	134	91	101	1.00E-31	142
KB425167.1	48639	1688	1871	*L. luteus*	l0itg12691	227	415	79.3	189	1.00E-27	129
KB425167.1	48639	1688	1871	*L. luteus*	l0itg12690	1101	913	79.3	189	1.00E-27	129
KB425167.1	48639	1688	1871	*L. luteus*	l0itg12689	1101	913	79.3	189	1.00E-27	129
KB425167.1	48639	3455	3548	*L. luteus*	l0itg32767	1062	969	90.4	94	1.00E-27	129
KB425167.1	48639	3668	3739	*L. luteus*	l0itg22569	1008	937	97.2	72	4.00E-27	127
KB425167.1	48639	3668	3739	*L. luteus*	l0itg32767	971	900	97.2	72	4.00E-27	127
KB425167.1	48639	7454	7552	*L. luteus*	l0itg32767	197	99	87.8	99	2.00E-25	121
KB425167.1	48639	2471	2535	*L. luteus*	l0itg22569	1162	1098	98.4	65	9.00E-25	119
KB425167.1	48639	11068	11327	*L. luteus*	l0itg52332	53	306	76.1	264	1.00E-23	116
KB425167.1	48639	11114	11413	*L. luteus*	l0itg25360	7	301	76.3	305	5.00E-23	114
KB425167.1	48639	3668	3739	*L. luteus*	l0itg46909	364	294	95.8	72	2.00E-22	112
KB425167.1	48639	6486	6552	*L. luteus*	l0itg22568	469	403	95.5	67	2.00E-22	112
KB425167.1	48639	6486	6552	*L. luteus*	l0itg22569	469	403	95.5	67	2.00E-22	112
KB425167.1	48639	22012	22113	*L. luteus*	l0itg20800	255	354	87.2	102	2.00E-22	112
KB425167.1	48639	2471	2532	*L. luteus*	l0itg32767	1125	1064	96.7	62	3.00E-21	108

Accession	Scaffold	Scaffold start	Scaffold end	Species	EST name	EST start	EST end	Percent identity	Alignment length	E-value	Score
KB425167.1	48639	5625	5695	*L. luteus*	l0itg22568	537	467	92.9	71	3.00E-21	108
KB425167.1	48639	5625	5695	*L. luteus*	l0itg22569	537	467	92.9	71	3.00E-21	108
KB425167.1	48639	11079	11178	*L. luteus*	l0itg32754	129	31	87	100	3.00E-21	108
KB430490.1	65565	14630	15396	*L. luteus*	l0itg47146	1582	823	92.6	767	0	1133
KB430490.1	65565	2288	2590	*L. luteus*	l0itg40955	476	174	95.7	303	9.00E-142	508
KB430490.1	65565	9313	9609	*L. luteus*	l0itg40676	535	831	95.6	297	3.00E-138	496
KB430490.1	65565	15614	15977	*L. luteus*	l0itg47146	633	280	87.6	365	2.00E-110	404
KB430490.1	65565	8867	9094	*L. luteus*	l0itg40676	309	536	96	228	2.00E-105	387
KB430490.1	65565	8876	9094	*L. luteus*	l0itg08411	508	290	93.1	219	1.00E-89	335
KB430490.1	65565	8876	9094	*L. luteus*	l0itg08410	342	560	93.1	219	1.00E-89	335
KB430490.1	65565	9774	10228	*L. luteus*	l0itg21330	373	829	82.4	462	6.00E-84	316
KB430490.1	65565	9313	9588	*L. luteus*	l0itg08411	291	13	86.7	279	2.00E-83	314
KB430490.1	65565	9313	9588	*L. luteus*	l0itg08410	559	837	86.7	279	2.00E-83	314
KB430490.1	65565	8053	8209	*L. luteus*	l0itg40676	153	309	96.1	157	2.00E-69	267
KB430490.1	65565	8053	8206	*L. luteus*	l0itg08411	673	520	96.1	154	1.00E-67	262
KB430490.1	65565	8053	8206	*L. luteus*	l0itg08410	177	330	96.1	154	1.00E-67	262
KB430490.1	65565	524	789	*L. luteus*	l0itg44971	310	45	82.7	267	7.00E-58	229
KB430490.1	65565	9870	10228	*L. luteus*	l0itg21331	3	359	81.2	363	6.00E-55	219
KB430490.1	65565	2047	2179	*L. luteus*	l0itg40955	614	476	92.8	139	3.00E-49	200
KB430490.1	65565	15429	15564	*L. luteus*	l0itg47146	823	687	90.5	137	8.00E-43	179
KB430490.1	65565	10128	10389	*L. luteus*	l0itg45774	324	62	79.1	264	1.00E-37	162
KB430490.1	65565	548	769	*L. luteus*	l0itg25555	219	2	80.6	222	5.00E-37	160
KB430490.1	65565	7703	7787	*L. luteus*	l0itg40676	70	154	98.8	85	2.00E-36	158
KB430490.1	65565	526	773	*L. luteus*	l0itg34676	95	347	79.1	254	3.00E-35	154
KB430490.1	65565	610	769	*L. luteus*	l0itg25556	160	2	84.3	160	3.00E-35	154
KB430490.1	65565	3186	3267	*L. luteus*	l0itg40955	174	93	98.7	82	1.00E-34	152
KB430490.1	65565	593	789	*L. luteus*	l0itg47635	892	700	83.2	197	4.00E-34	150
KB430490.1	65565	648	789	*L. luteus*	l0itg56281	1	141	85.9	142	1.00E-33	148
KB430490.1	65565	18068	18168	*L. luteus*	l0itg06136	987	887	92	101	1.00E-33	148
KB430490.1	65565	526	768	*L. luteus*	l0itg13351	535	775	78.1	243	8.00E-32	142
KB430490.1	65565	1	112	*L. luteus*	l0itg02088	278	391	90.3	114	4.00E-30	137
KB430490.1	65565	1	112	*L. luteus*	l0itg02086	500	613	90.3	114	4.00E-30	137
KB430490.1	65565	1	112	*L. luteus*	l0itg02085	500	613	90.3	114	4.00E-30	137
KB430490.1	65565	1	112	*L. luteus*	l0itg02083	278	391	90.3	114	4.00E-30	137
KB430490.1	65565	1	112	*L. luteus*	l0itg02079	2056	2169	90.3	114	4.00E-30	137
KB430490.1	65565	1	112	*L. luteus*	l0itg02078	2056	2169	90.3	114	4.00E-30	137
KB430490.1	65565	1	112	*L. luteus*	l0itg02076	646	759	90.3	114	4.00E-30	137
KB430490.1	65565	1	112	*L. luteus*	l0itg02075	608	721	90.3	114	4.00E-30	137
KB430490.1	65565	1	112	*L. luteus*	l0itg02074	608	721	90.3	114	4.00E-30	137
KB430490.1	65565	1	112	*L. luteus*	l0itg02073	646	759	90.3	114	4.00E-30	137
KB430490.1	65565	15741	15848	*L. luteus*	l0itg47146	390	282	89.9	109	4.00E-30	137
KB430490.1	65565	18065	18170	*L. luteus*	l0itg20145	144	39	88.6	106	2.00E-29	135
KB430490.1	65565	6613	6943	*L. luteus*	l0itg26027	356	28	76.4	335	2.00E-28	131
KB430490.1	65565	6613	6943	*L. luteus*	l0itg26026	102	430	76.4	335	2.00E-28	131
KB430490.1	65565	15866	16012	*L. luteus*	l0itg47146	509	361	86	151	2.00E-28	131
KB430490.1	65565	18061	18173	*L. luteus*	l0itg37838	286	174	86.7	113	2.00E-28	131
KB430490.1	65565	18068	18168	*L. luteus*	l0itg06134	974	874	89.1	101	2.00E-28	131
KB430490.1	65565	18068	18168	*L. luteus*	l0itg06133	974	874	89.1	101	2.00E-28	131
KB430490.1	65565	7703	7787	*L. luteus*	l0itg08411	756	672	92.9	85	9.00E-28	129
KB430490.1	65565	7703	7787	*L. luteus*	l0itg08410	94	178	92.9	85	9.00E-28	129
KB430490.1	65565	4	112	*L. luteus*	l0itg26991	168	59	88.1	110	3.00E-27	127
KB430490.1	65565	651	790	*L. luteus*	l0itg35427	20	159	82.1	140	1.00E-26	125
KB430490.1	65565	526	768	*L. luteus*	l0itg13352	341	582	76.1	243	5.00E-26	123
KB430490.1	65565	651	773	*L. luteus*	l0itg47635	532	652	85.3	123	5.00E-26	123
KB430490.1	65565	693	789	*L. luteus*	l0itg48746	110	206	88.6	97	5.00E-26	123
KB430490.1	65565	549	780	*L. luteus*	l0itg53437	548	326	79.3	232	2.00E-25	121
KB430490.1	65565	551	752	*L. luteus*	l0itg33108	209	10	78.7	202	2.00E-25	121
KB430490.1	65565	10268	10392	*L. luteus*	l0itg39598	9	133	83.2	125	7.00E-25	119
KB430490.1	65565	13823	13893	*L. luteus*	l0itg01771	224	294	95.7	71	7.00E-25	119

Accession	Scaffold	Scaffold start	Scaffold end	Species	EST name	EST start	EST end	Percent identity	Alignment length	E-value	Score
KB430490.1	65565	18068	18171	*L. luteus*	l0itg37164	358	461	86.5	104	7.00E-25	119
KB430490.1	65565	18	112	*L. luteus*	l0itg36317	129	224	89.5	96	3.00E-24	117
KB430490.1	65565	512	744	*L. luteus*	l0itg33785	437	220	76.8	233	3.00E-24	117
KB430490.1	65565	525	768	*L. luteus*	l0itg13350	596	836	77	244	3.00E-24	117
KB430490.1	65565	642	769	*L. luteus*	l0itg32448	383	511	83.7	129	3.00E-24	117
KB430490.1	65565	23	112	*L. luteus*	l0itg17604	73	163	90.1	91	4.00E-23	114
KB430490.1	65565	23	112	*L. luteus*	l0itg17603	73	163	90.1	91	4.00E-23	114
KB430490.1	65565	649	769	*L. luteus*	l0itg39710	265	384	84.2	121	4.00E-23	114
KB430490.1	65565	12272	12447	*L. luteus*	l0itg47471	290	115	79.6	177	4.00E-23	114
KB430490.1	65565	640	794	*L. luteus*	l0itg14407	621	469	81.2	155	1.00E-22	112
KB430490.1	65565	11662	11812	*L. luteus*	l0itg42515	17	167	79.4	151	1.00E-22	112
KB430490.1	65565	2	77	*L. luteus*	l0itg47820	96	20	93.5	77	5.00E-22	110
KB430490.1	65565	524	794	*L. luteus*	l0itg14408	734	469	76.7	271	5.00E-22	110
KB430490.1	65565	11663	11802	*L. luteus*	l0itg50589	402	540	81.4	140	5.00E-22	110
KB430490.1	65565	3879	3937	*L. luteus*	l0itg40955	93	35	98.3	59	2.00E-21	108
KB430490.1	65565	11662	11804	*L. luteus*	l0itg08829	546	404	79.7	143	2.00E-21	108
KB430490.1	65565	11662	11804	*L. luteus*	l0itg08827	546	404	79.7	143	2.00E-21	108
KB430490.1	65565	18056	18171	*L. luteus*	l0itg54968	300	415	82.7	116	2.00E-21	108
KB430490.1	65565	18062	18171	*L. luteus*	l0itg11664	115	6	83.6	110	2.00E-21	108
KB430490.1	65565	10313	10391	*L. luteus*	l0itg51342	83	161	89.8	79	8.00E-21	106
KB430490.1	65565	11662	11832	*L. luteus*	l0itg34163	189	21	78.3	171	8.00E-21	106
KB430490.1	65565	23	113	*L. luteus*	l0itg09019	178	269	88	92	3.00E-20	104
KB430490.1	65565	23	113	*L. luteus*	l0itg09017	405	496	88	92	3.00E-20	104

Supplementary Table 14. List of transcripts identified in the annotated soybean genome by BLAST analysis of *L. angustifolius CHIL* sequences.

***L. angustifolius CHIL* gene**	***G. max* chromosome**	**Chromosome locus (kbp)**	**Transcript name**	**Total score**	**Percent identity**
*LangCHIL*1	Gm06	11616	Glyma06g14820	992.6	86.1
*LangCHIL*1	Gm04	46176	Glyma04g40030	882.6	74.8
*LangCHIL*1	Gm09	20428	-	259.2	76.9
*LangCHIL*1	Gm10	9604	-	205.1	82.3
*LangCHIL*2	Gm06	11616	Glyma06g14820	1054.3	80.5
*LangCHIL*2	Gm04	46176	Glyma04g40030	910.5	77.8
*LangCHIL*2	Gm09	20428	-	250.2	75.1
CHIL2	Gm10	9604	-	190.6	81.0

Supplementary Table 15. Conserved sequence blocks shared between *G. max* and *M. truncatula*, *L. japonicus*, *C. arietinum*, *P. vulgaris*, and *C. cajan* genomes in regions having syntenic links to the *L. angustifolius* contigs.

G. max chromosome	Position of conserved block (kb)	Length of conserved block (kb)	Reference genomic sequence showing shared synteny	Position of conserved block (kb)	Length of conserved block (kb)
4	46160	307	*C. cajan* scaf. 000011	944	496
	46100	50	*C. cajan* scaf. 137179	32	107
	44624	3844	*C. arietinum* chr. 5	43890	4168
	44335	3193	*L. japonicus* chr. 1	12901	4369

	43604	4883	*M. truncatula* chr. 3	39919	5600
	46186	308	*P. vulgaris* chr. 9	7995	558
	46309	2559	*P. vulgaris* chr. 9	17925	2144
6	11302	327	*C. cajan* scaf. 000011	944	496
	11635	65	*C. cajan* scaf. 137179	32	118
	9163	4168	*C. arietinum* chr. 5	43891	4167
	9947	2082	*L. japonicus* chr. 1	12901	4238
	9163	5999	*M. truncatula* chr. 3	39878	4953
	11375	207	*P. vulgaris* chr. 9	8194	314
	10833	1253	*P. vulgaris* chr. 9	20268	991
7	7677	212	*C. cajan* chr. 6	19451	266
	7731	1631	*C. arietinum* chr. 8	14152	1247
	8054	344	*L. japonicus* chr. 2	11685	665
	7735	1922	*M. truncatula* chr. 6	29957	3129
	7384	2978	*P. vulgaris* chr. 4	42719	2510
9	38895	288	*C. cajan* chr. 6	19193	524
	38701	473	*C. arietinum* chr. 8	14961	616
	38399	317	*L. japonicus* chr. 2	11685	626
	37826	1406	*M. truncatula* chr. 6	30600	3003
	30170	9028	*P. vulgaris* chr. 4	35690	9420
