# Supplementary material for: Structure, expression profile and phylogenetic inference of chalcone isomerase-like genes from the narrow-leafed lupin (Lupinus angustifolius L.) genome
Source: Front Plant Sci. 2015 Apr 21;6:268. doi: 10.3389/fpls.2015.00268 (PMC4404975; doi:10.3389/fpls.2015.00268)
Supplement: Supplementary file 2 [file Presentation1.PDF]

Supplementary Material 2

Structure, expression profile and phylogenetic inference of chalcone isomerase-like genes from the narrow-leafed lupin (*Lupinus angustifolius* L.) genome

Łucja Przysiecka [1,2], Michał Książkiewicz[1*], Bogdan Wolko[1], Barbara Naganowska[1]

[1] Department of Genomics, Institute of Plant Genetics of the Polish Academy of Sciences, Poznań, Poland
[2] NanoBioMedical Centre, Adam Mickiewicz University, Poznań, Poland

*** Correspondence:** Dr. Michał Książkiewicz, Department of Genomics, Institute of Plant Genetics of the Polish Academy of Sciences, Strzeszyńska 34, Poznań, 60-479, Poland.
mksi@igr.poznan.pl

1. Supplementary Figures

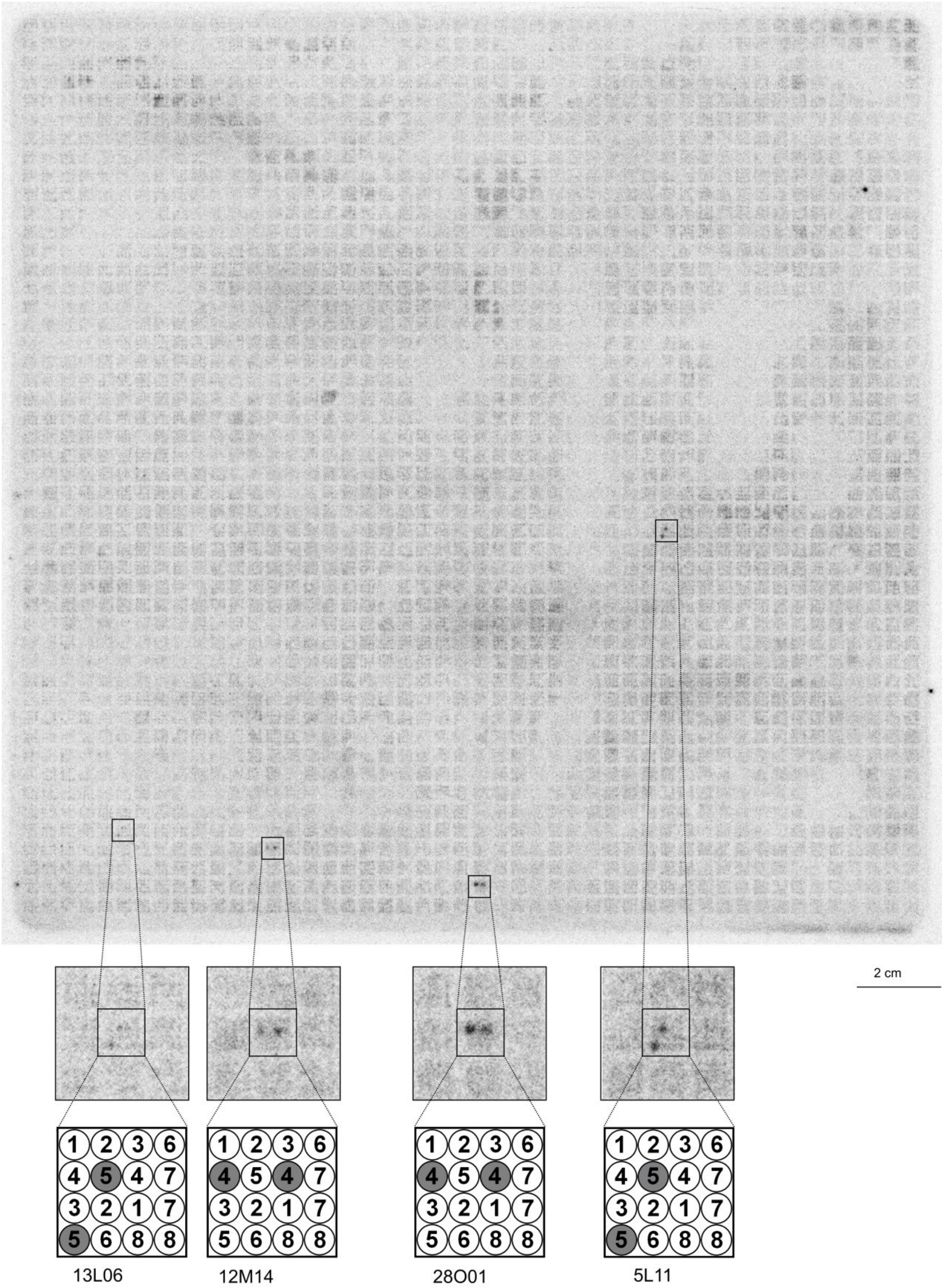

Supplementary Figure 1. Macroarray containing DNA of 18 432 clones subjected to hybridization with the *CHIL* probe. BAC clones were arrayed in 6 large blocks carrying 16 spots (8 clones, 2 repeats) x 16 vertical lines and 24 vertical lines. The enlarged fragments show the arrangement of post-hybridization signals and their coordinate system.

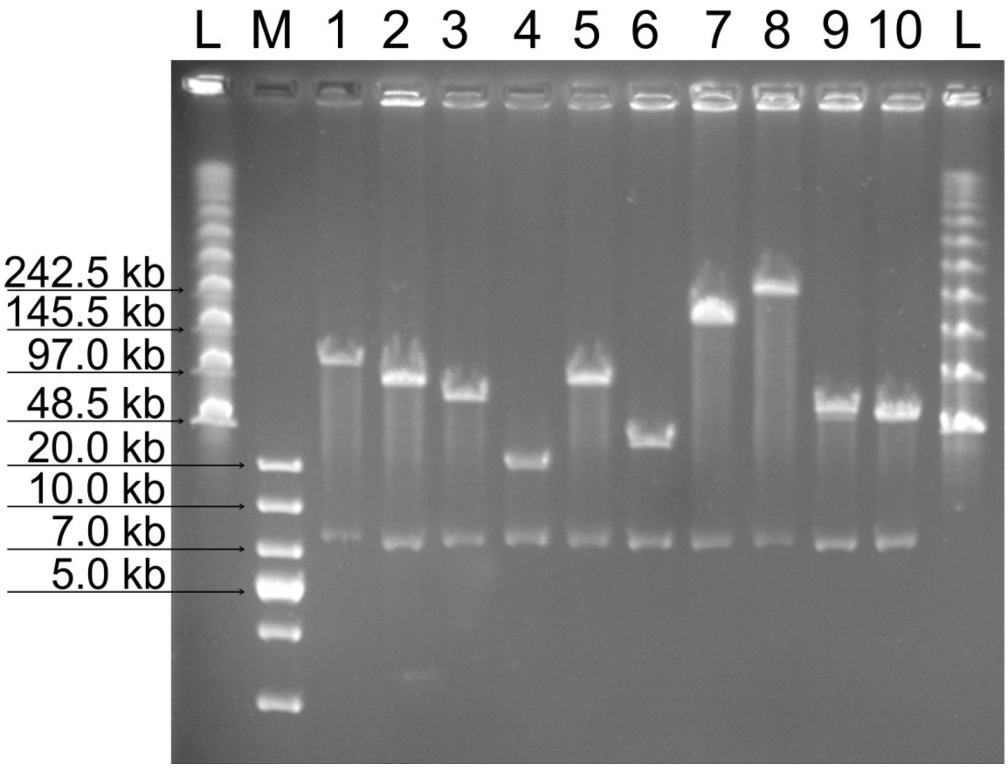

Supplementary Figure 2. PFGE of insert DNA from selected clones from the *L. angustifolius* BAC library. Clones were digested using *Not*I restriction enzyme. L, Lambda ladder PFG marker (New England BioLabs); M, O'GeneRuler 1 kb Plus DNA Ladder (Fermentas), 1-10 BAC clones: 5L11, 12M14, 28O01, 41I07, 88J04, 106M03, 115N04, 115L05, 129C12, 134F01.

Contig 1

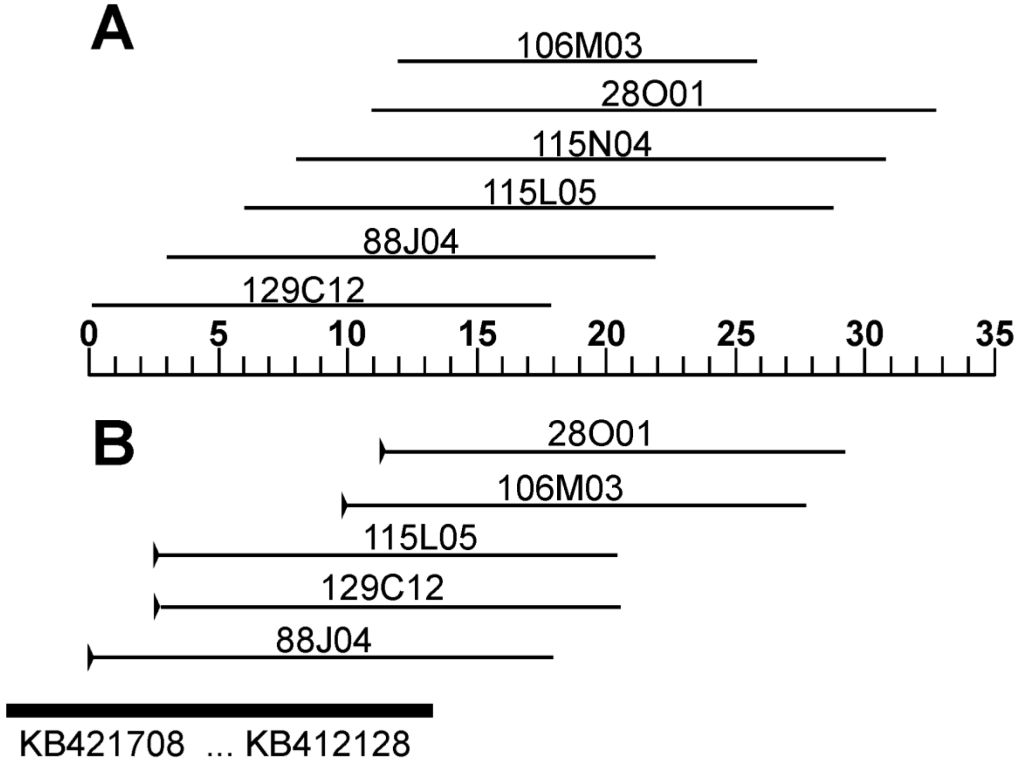

Contig 2

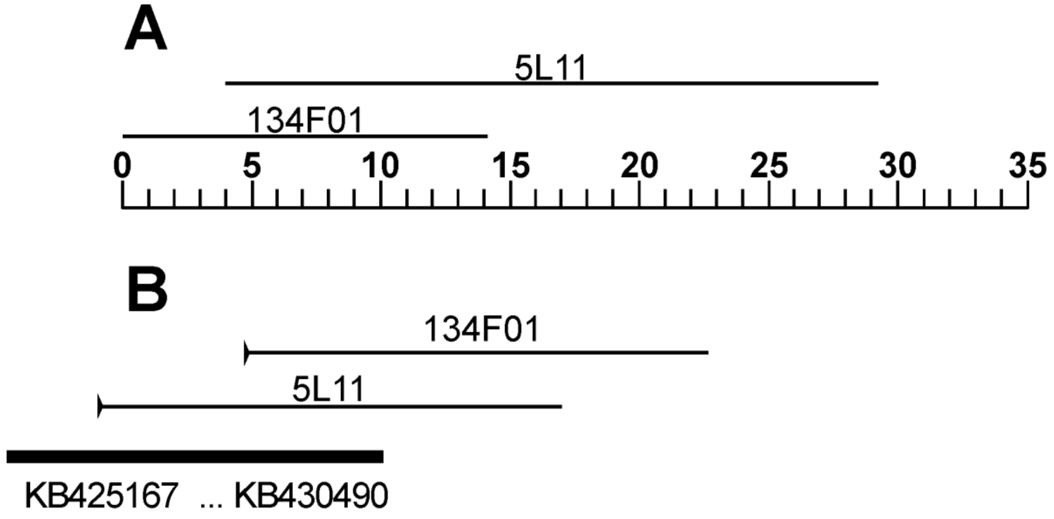

Supplementary Figure 3. Structure of BAC contigs constructed using *Hind*III and *Eco*130I fingerprint data. The scale is expressed in consensus band (CB) units. A - contig structure derived from restriction enzyme fingerprinting, B - contig structure derived from mapping of BESs to scaffold sequences.

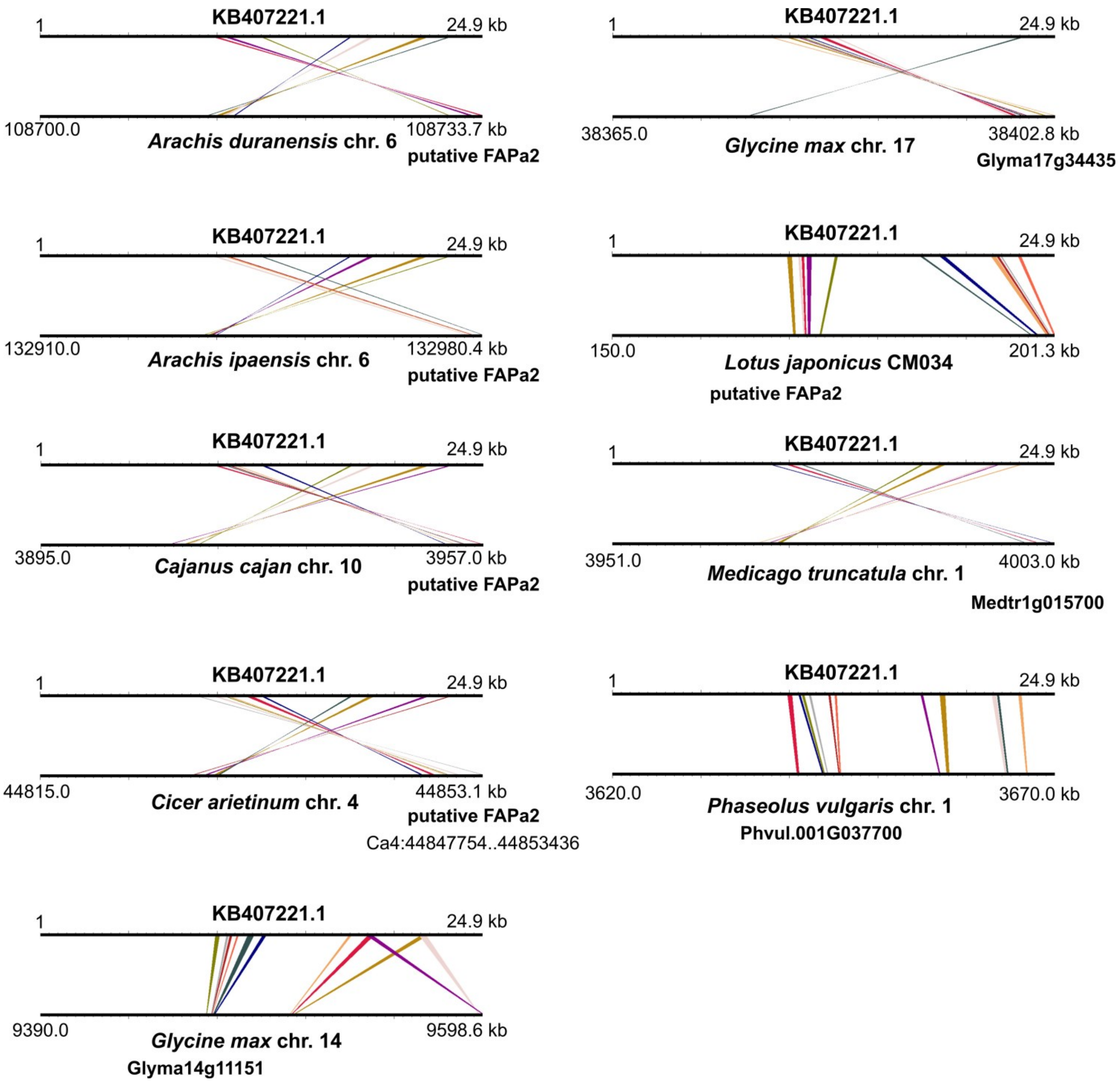
KB407221.1
1
24.9 kb
108700.0
108733.7 kb
Arachis duranensis chr. 6
putative FAPa2
KB407221.1
1
24.9 kb
38365.0
38402.8 kb
Glycine max chr. 17
Glyma17g34435
KB407221.1
1
24.9 kb
132910.0
132980.4 kb
Arachis ipaensis chr. 6
putative FAPa2
KB407221.1
1
24.9 kb
150.0
201.3 kb
Lotus japonicus CM034
putative FAPa2
KB407221.1
1
24.9 kb
3895.0
3957.0 kb
Cajanus cajan chr. 10
putative FAPa2
KB407221.1
1
24.9 kb
3951.0
4003.0 kb
Medicago truncatula chr. 1
Medtr1g015700
KB407221.1
1
24.9 kb
44815.0
44853.1 kb
Cicer arietinum chr. 4
putative FAPa2
Ca4:44847754..44853436
KB407221.1
1
24.9 kb
3620.0
3670.0 kb
Phaseolus vulgaris chr. 1
Phvul.001G037700
KB407221.1
1
24.9 kb
9390.0
9598.6 kb
Glycine max chr. 14
Glyma14g11151

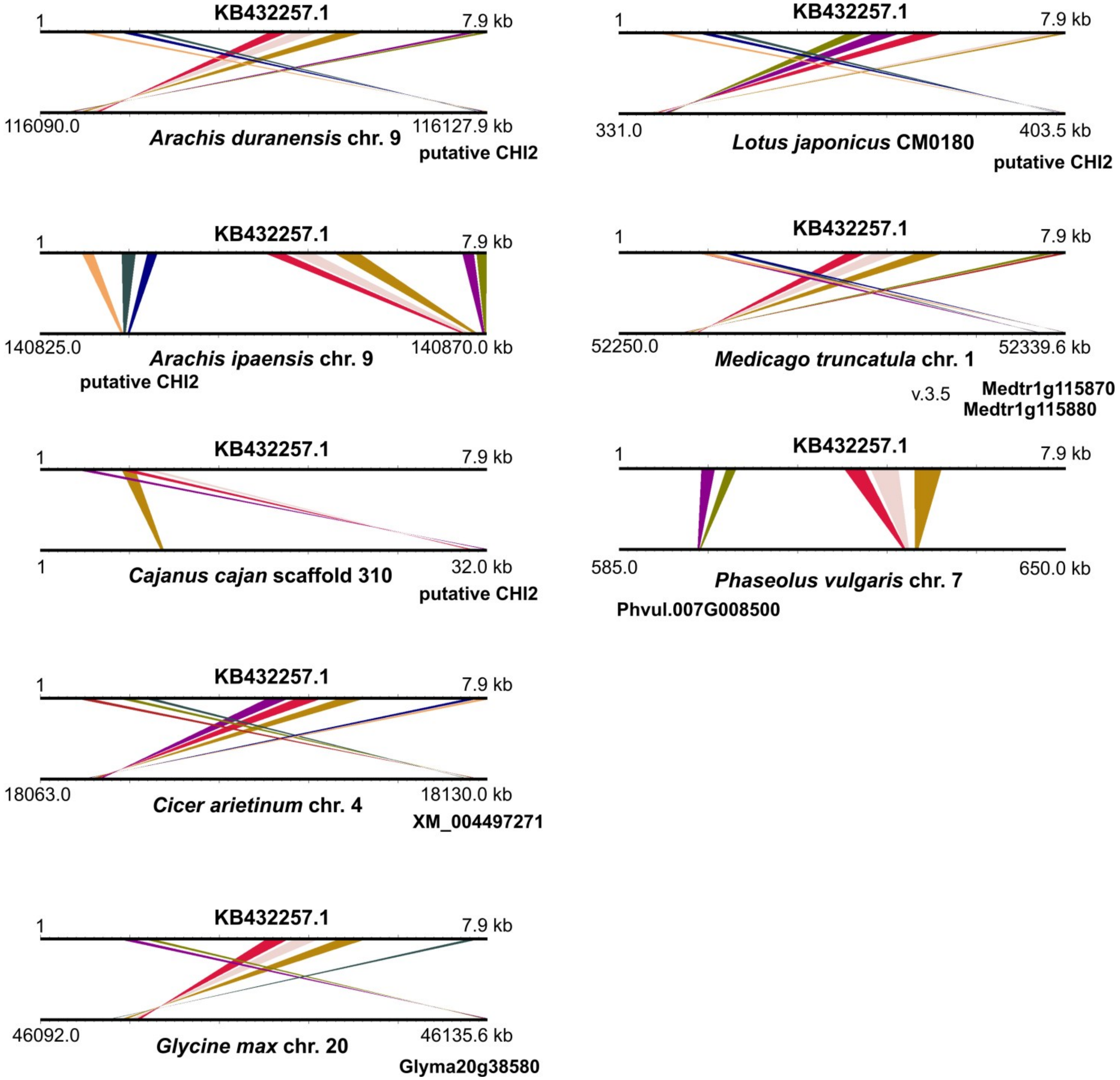

KB432257.1
1
7.9 kb
116090.0
116127.9 kb
Arachis duranensis chr. 9
putative CHI2
KB432257.1
1
7.9 kb
331.0
403.5 kb
Lotus japonicus CM0180
putative CHI2
KB432257.1
1
7.9 kb
140825.0
140870.0 kb
Arachis ipaensis chr. 9
putative CHI2
KB432257.1
1
7.9 kb
52250.0
52339.6 kb
Medicago truncatula chr. 1
v.3.5
Medtr1g115870
Medtr1g115880
KB432257.1
1
7.9 kb
1
32.0 kb
Cajanus cajan scaffold 310
putative CHI2
KB432257.1
1
7.9 kb
585.0
650.0 kb
Phaseolus vulgaris chr. 7
Phvul.007G008500
KB432257.1
1
7.9 kb
18063.0
18130.0 kb
Cicer arietinum chr. 4
XM_004497271
KB432257.1
1
7.9 kb
46092.0
46135.6 kb
Glycine max chr. 20
Glyma20g38580

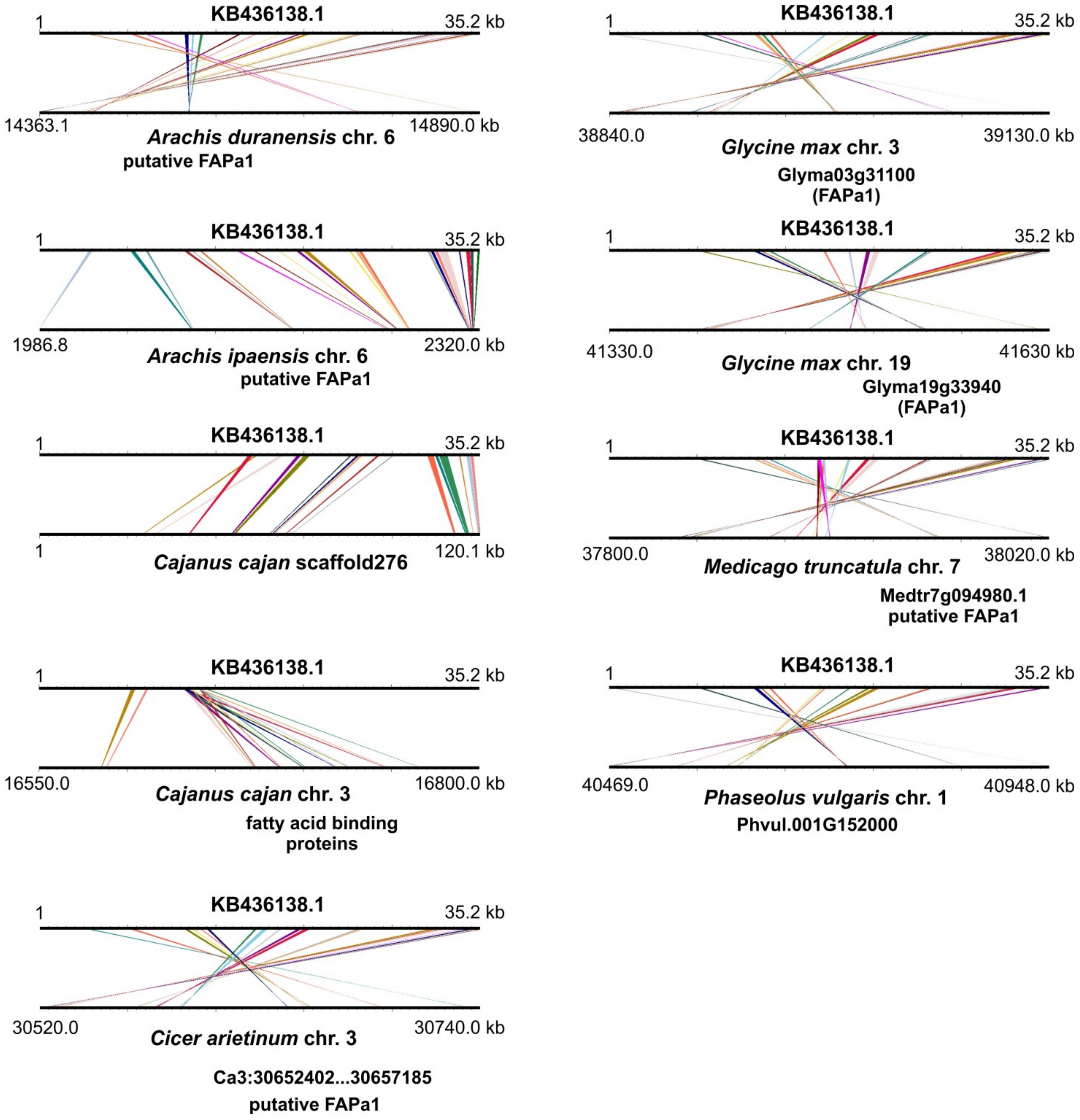
KB436138.1
1
35.2 kb
14363.1
14890.0 kb
Arachis duranensis chr. 6
putative FAPa1
KB436138.1
1
35.2 kb
38840.0
39130.0 kb
Glycine max chr. 3
Glyma03g31100
(FAPa1)
KB436138.1
1
35.2 kb
1986.8
2320.0 kb
Arachis ipaensis chr. 6
putative FAPa1
KB436138.1
1
35.2 kb
41330.0
41630 kb
Glycine max chr. 19
Glyma19g33940
(FAPa1)
KB436138.1
1
35.2 kb
1
120.1 kb
Cajanus cajan scaffold276
KB436138.1
1
35.2 kb
37800.0
38020.0 kb
Medicago truncatula chr. 7
Medtr7g094980.1
putative FAPa1
KB436138.1
1
35.2 kb
16550.0
16800.0 kb
Cajanus cajan chr. 3
fatty acid binding
proteins
KB436138.1
1
35.2 kb
40469.0
40948.0 kb
Phaseolus vulgaris chr. 1
Phvul.001G152000
KB436138.1
1
35.2 kb
30520.0
30740.0 kb
Cicer arietinum chr. 3
Ca3:30652402...30657185
putative FAPa1

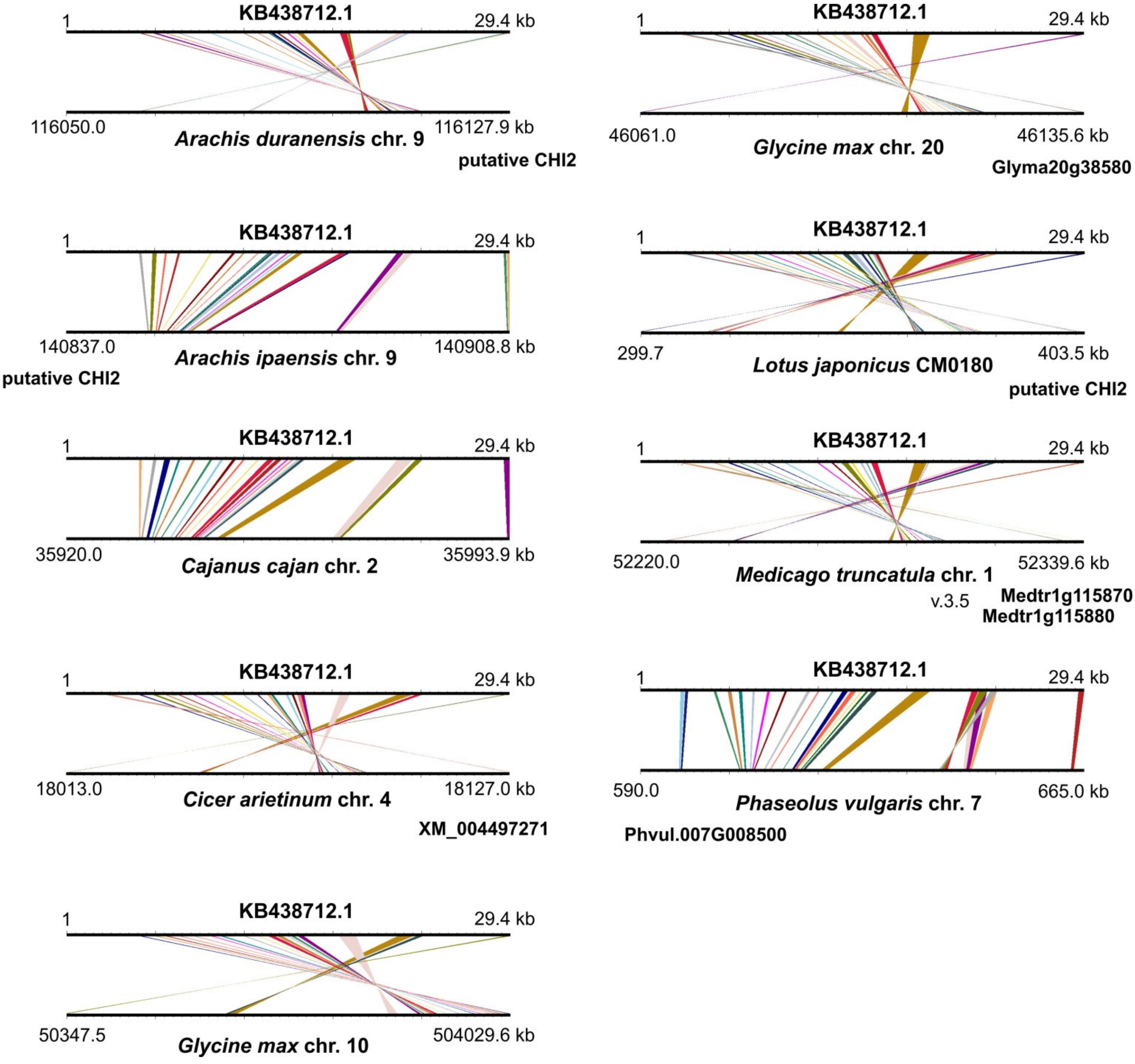

Supplementary Figure 4. Microsyntenic links of four *L. angustifolius* scaffolds carrying CHI-fold protein family genes. Microsyntenic links between the scaffolds and genomic sequences of eight legume species (*Arachis duranensis*, *Arachis ipaensis*, *Cajanus Cajan*, *Cicer arietinum*, *Glycine max*, *Lotus japonicus*, *Medicago truncatula*, *Phaseolus vulgaris*) are shown on Genome Synteny Viewer diagrams (Revanna et al., 2011), below the gray bar. Each diagram comprises two horizontal lines. The upper line shows the sequence of *L. angustifolius* scaffold. The lower line shows the corresponding region of a model legume genome. Chromosome localizations (nt) are given. Annotated CHI-fold genes are presented as gene locus names (if annotation was already assigned in the genome sequence) or locus positions with putative annotation based on sequence similarity to reference CHI-fold proteins. Arrows indicate that CHI-fold gene was identified outside the syntenic region presented on the graph.

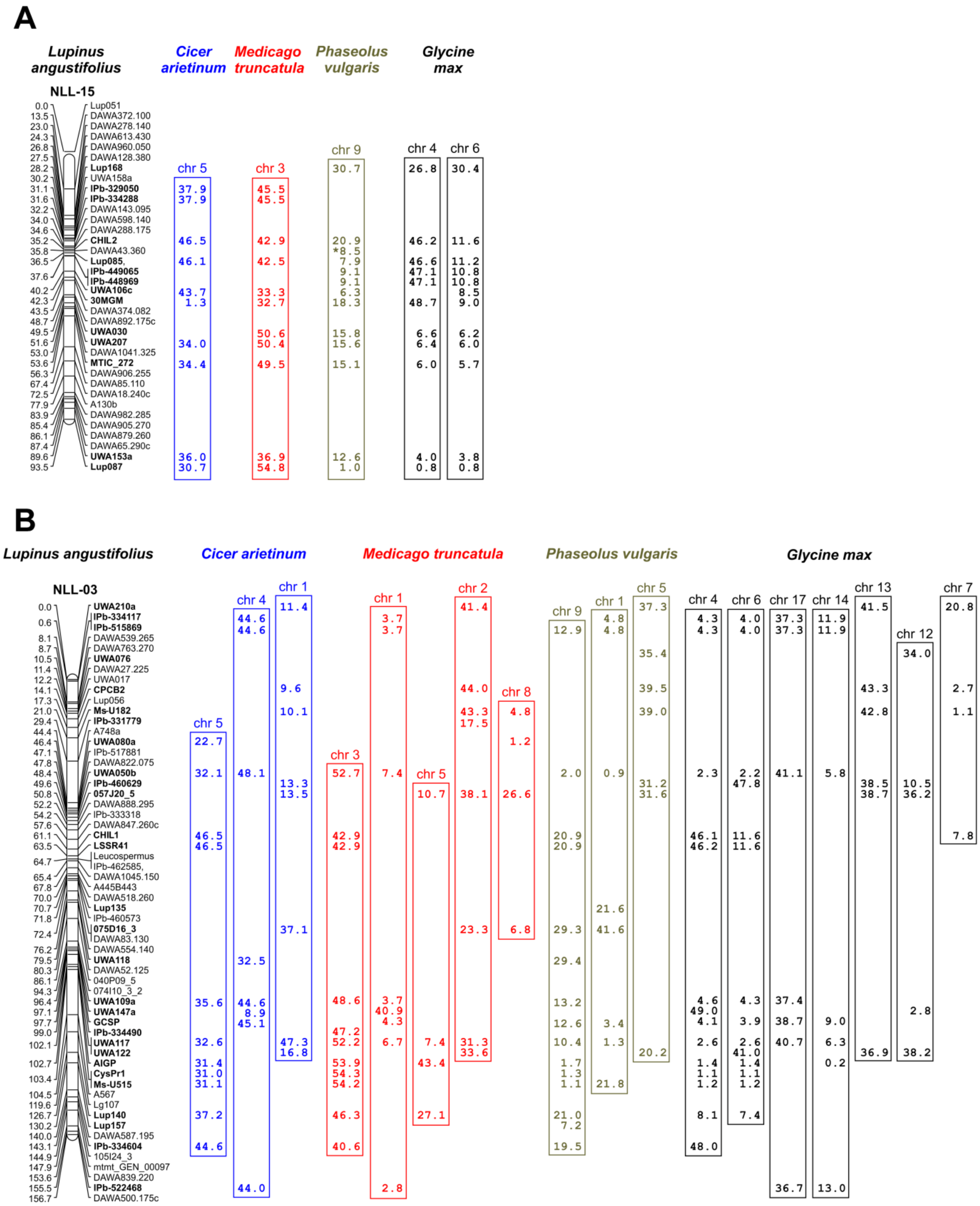

Supplementary Figure 5. Macrosyntenic links identified between the chromosome sequences from four reference legume species and the sequences of *L. angustifolius* molecular markers from linkage groups NLL-15 (A) and NLL-03 (B). Positions of syntenic loci in legume chromosomes are given in megabases, whereas marker positions on the *L. angustifolius* linkage groups are in centimorgans. The borders of the bars do not indicate chromosome lengths, but are determined by the two syntenic regions mapped most distantly on the respective *L. angustifolius* linkage group. Asterisk (*) indicates the second syntenic locus mapped to CHIL2 marker.
